# Synthesis and biological evaluation of thiosemicarbazone-based antibody–drug conjugates†

**DOI:** 10.1039/d5md00154d

**Published:** 2025-06-26

**Authors:** Nandan Sheernaly, Irene Shajan, Axel Steinbrueck, Bauke Albada, Nils Metzler-Nolte

**Affiliations:** a Faculty of Chemistry and Biochemistry, Inorganic Chemistry I – Bioinorganic Chemistry, Ruhr-University Bochum Universitaetsstrasse 150 44801 Bochum Germany nils.metzler-nolte@ruhr-uni-bochum.de; b Laboratory of Organic Chemistry, Wageningen University & Research Stippeneng 4 Wageningen 6708 WE The Netherlands

## Abstract

Metal chelators belonging to the di-pyridyl-thiosemicarbazone (DpTs) class have shown great promise as adjuvant therapeutics for treating cancer, with DpC and Dp44mT emerging as the lead candidates. Despite their efficacy, these molecules also induce various undesirable side effects due to insufficient cancer cell targeting, highlighting the need to improve their selectivity. Here, we present a first generation of DpT–antibody conjugates. To this end, we developed a facile synthesis to functionalize DpTs strategically with click-able azido linkers. Moreover, selective side-chain modification of the clinical antibody trastuzumab (Tras) with a complementary bis-alkyne moiety is described. Using this new chemistry, we conjugated four different azido DpTs to trastuzumab *via* a combination of oxidation-controlled quinone (SPOCQ) and strain-promoted alkyne–azide click (SPAAC) chemistry. We evaluated the antiproliferative activity of the resulting novel antibody–drug conjugates (ADCs) against MCF-7 and SK-BR-3 cell lines. Linker positioning on the DpT scaffold significantly influences the cytotoxicity of the conjugates. For instance, conjugating Tras at the *ortho* position on the Dp44mT scaffold is more efficacious than conjugating at the *para* position with IC_50_ values of 25.7 ± 5.5 nM and 103.5 ± 2.0 nM, respectively, against MCF-7 cells. Furthermore, we observe intriguing cell line-dependent activity of the ADCs with increased selectivity towards MCF-7 cells, providing novel insights into the cytotoxic activity of DpTs and their antibody conjugates.

## Abstract 2

2-Dipyridyl-3-thiosemicarbazones were conjugated to trastuzumab *via ortho*-quinone cycloaddition and azide–alkyne click chemistry. The novel ADCs showed intriguing cell-line dependent activity, with the payload overriding antibody selectivity.

## Graphical abstract

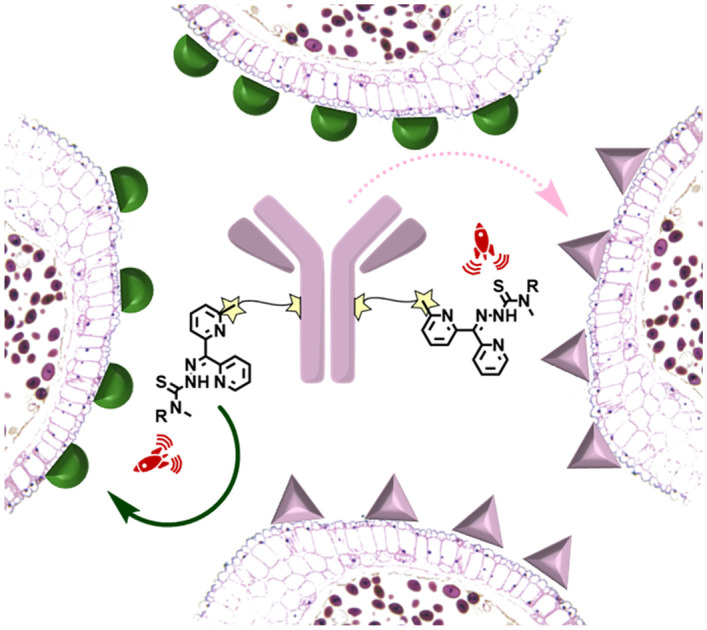

## 1. Introduction

**Fig. 1:**
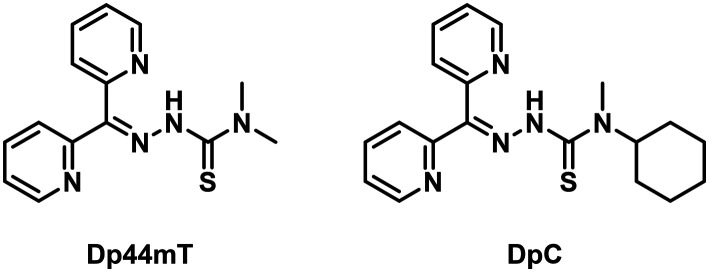
Examples of thiosemicarbazones established as promising anticancer agents.^13,17^

Thiosemicarbazones have been increasingly recognized for their potential as effective anticancer agents.^1^ Specifically, those belonging to the di-2-pyridyl-thiosemicarbazone (DpT) class have shown great promise.^2^ The hallmark of DpTs is their NNS binding motif that facilitates coordination to metal ions such as Cu(ii) and Fe(ii).^3^ Metal-ion coordination affects various cellular targets, ultimately impacting the viability of cells by, for instance, inhibition of ribonucleotide reductase,^4^ upregulation of *N-myc downstream regulated gene 1* (NDRG1),^5,6^ downregulation of cyclin D1 leading to cell cycle arrest,^7^ as well as others.^2,3,8^ Among the many DpTs studied over the years, di-2-pyridylketone-4,4-dimethyl-3-thiosemicarbazone (Dp44mT) and di-2-pyridylketone-4-cyclohexyl-4-methyl-3-thiosemicarbazone (DpC) (Fig. 1) emerged as the most prominent candidates by exhibiting excellent selective antiproliferative activity both *in vitro* and *in vivo* against a wide variety of cancer types.^2,9^ For example, these two compounds substantially suppress tumor growth and metastasis in murine xenograft models of pancreatic cancer and osteosarcoma.^10,11^ However, Dp44mT displays side effects like cardiac fibrosis and methemoglobin formation at higher doses.^2,12,13^ DpC, on the other hand, has demonstrated superior *in vivo* tolerability and anticancer activity compared to Dp44mT while minimizing systemic toxicity.^14–16^ Consequently, DpC entered phase 1 clinical trials in 2016 (NCT02688101), which concluded in 2019. Despite mitigating many of the drawbacks of Dp44mT, DpC still required treatments in precise dosing and caused pulmonary inflammation at higher doses.^16^ Therefore, the need to improve the selectivity and the therapeutic index of these molecules persists as their full potential remains untapped.

A particularly effective approach to address selectivity limitations is the conjugation of drug molecules to cell-targeting antibodies. Over the years, several antibody–drug conjugates (ADCs) that combine the targeting ability of monoclonal antibodies (mAbs) with potent cytotoxic effects of drug payloads have been developed.^18^ Ideally, a mAb's targeting ability allows precise delivery of the conjugated drug to the tumor site, thereby reducing systemic side effects and broadening the therapeutic window of the drug.^19,20^ One of the first ADCs approved by the FDA (trastuzumab emtansine (T-DM1)) was based on trastuzumab (Tras), a mAb that targets and binds the HER2 receptors in cancer cells. The binding is known to inhibit signaling pathways promoted by the HER2 receptor, hampering cell proliferation and survival.^21,22^ While Tras itself has been immensely successful in treating HER2-positive breast cancer, the use of T-DM1 has helped to combat unwanted resistance development and reduce the serious side effects of unconjugated emtansine, such as cardiac toxicity.^23^ The advent of T-DM1 proved to be pivotal in the development of ADCs as it pioneered the subsequent approval of several ADCs for different cancers.^19,24,25^

Trastuzumab inhibits downstream pathways like mitogen-activated protein kinase/extracellular signal-regulated kinase (MAPK/ERK) and phosphatidylinositol-3 kinase (PI3K)/AKT, making cancer cells susceptible to cell death-inducing agents like the DpTs.^26,27^*A priori*, we hypothesized that conjugating DpTs to Tras could display synergistic effects, considering that the DpTs upon iron chelation are also known to affect similar cellular pathways with respect to Tras.^5,28–30^ Here, we employed strain-promoted oxidation-controlled cyclooctyne–1,2-quinone (SPOCQ) cycloaddition technology and strain-promoted azide–alkyne click reaction (SPAAC) for the antibody modification and conjugation of DpTs to Tras.^31,32^ Further, we explored the effects of conjugating the mAb to different positions of the DpT structural motif on the antiproliferative activity of the synthesized ADCs.

## 2. Results and discussion

### Synthesis of the azido functionalized DpTs

**Fig. 2:**
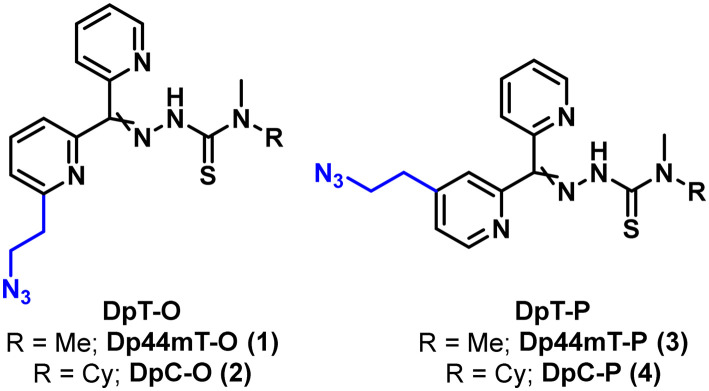
DpTs functionalized at positions 4 (DpT-O) and 6 (DpT-P) with an azidoethyl moiety.

Our first focus was to equip Dp44mT and DpC with an azido functionality to enable bioconjugation to Tras by means of the azide–alkyne cycloaddition reaction. To preserve the backbone of the DpTs, we decided to functionalize one of the pyridyl rings with the desired azido functionality (Fig. 2) instead of employing a previously reported method of derivatizing thiosemicarbazones at the thiocarbonyl group *via* a transamidation reaction.^33^

**Scheme 1:**
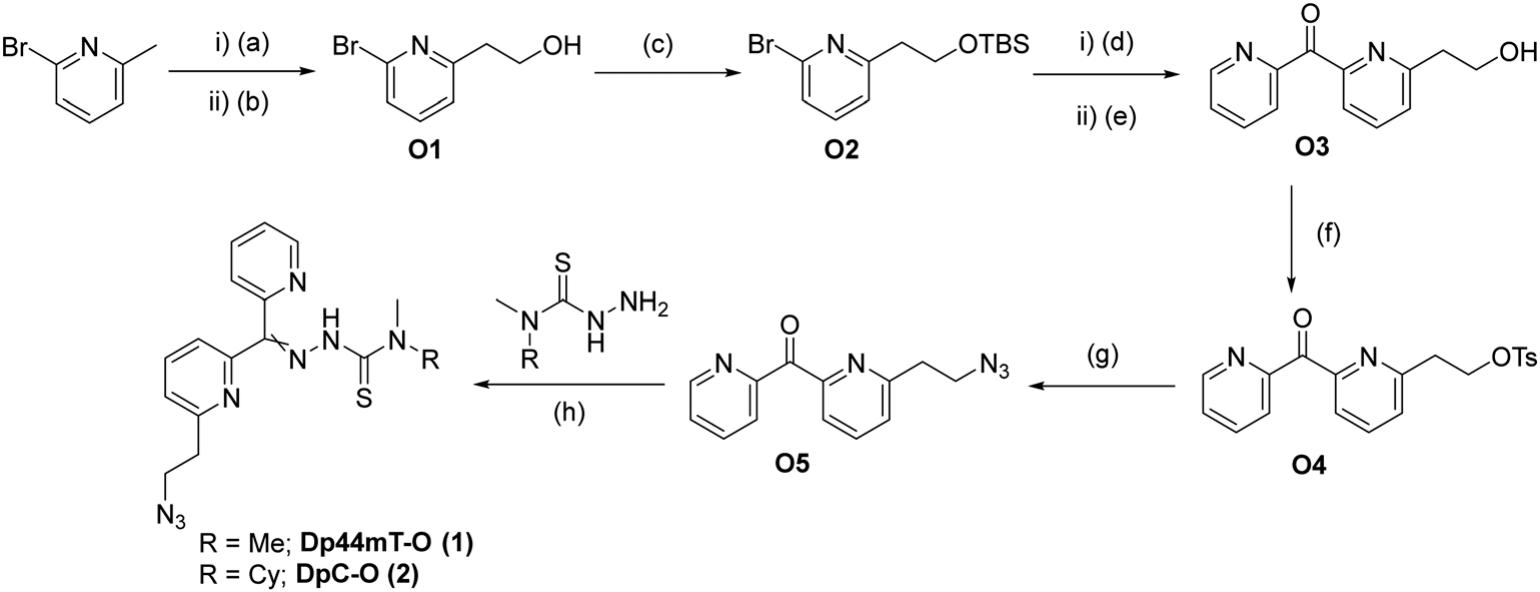
Synthetic route leading to DpC-O and Dp44mT-O, (a) LDA, DMF, THF, −78 °C, 30 min; (b) acetic acid, MeOH, NaBH_4_, rt, 16 h, 62%; (c) TBSCl, imidazole, DMF, rt, 16 h, 88%; (d) *n*-BuLi, 2-cyanopyridine, Et_2_O, −78 °C, 30 min; (e) 1 M HCl, 50 °C, 3 h, 42%; (f) TsCl, Et_3_N, 3 h, 83%; (g) NaN_3_, DMF, 80 °C, 16 h, 98%; (h) AcOH (few drops), EtOH, reflux, 1 h, 92%.

Considering that the DpTs utilize Cu(ii) and Fe(ii) chelation as one of their primary modes of action,^3^ we envisioned that positioning of the linker is crucial to retain biological activity. Therefore, we decided to place the azido linker at two different positions to compare the effects of the linker positioning on the activities of the modified toxins and resulting ADCs. We developed a versatile synthetic route that allows us to procure both isomers *via* similar approaches, as illustrated for the *ortho* derivatives in Scheme 1 below. Synthesis of the representative DpT-O commenced with the formation of 2-pyridylethanol O1 by treating 2-bromo-6-methylpyridine with DMF, followed by reduction of the resulting aldehyde using NaBH_4_. After protecting the alcohol with TBS, O2 was reacted with 2-cyanopyridine to yield ketone O3. During the acidic work-up, the alcohol was conveniently deprotected *via* acid hydrolysis, and O3 was then subjected to functional group interconversion to give rise to the azide O5 over two steps. Finally, the latter was condensed with the respective thiosemicarbazide to obtain the DpT-Os (1 and 2) in good yields (*cf.* ESI†). The corresponding *para*-substituted derivatives DpT-Ps (3 and 4) were similarly acquired using 2-bromo-4-methylpyridine as the starting material in moderate yields (*cf.* ESI†).

The final compounds 1–4 were each isolated as an inseparable mixture of *E*/*Z* isomers in approximately 1 : 1 ratio; ^1^H NMR spectra of all synthesized DpTs exhibited twice the number of expected signals (*e.g.*, Fig. S25†). Of note, for compounds 1 and 2, the isomers could be distinguished and identified using NOESY and COSY experiments.

### Antiproliferative activity of DpT-azides

**Fig. 3:**
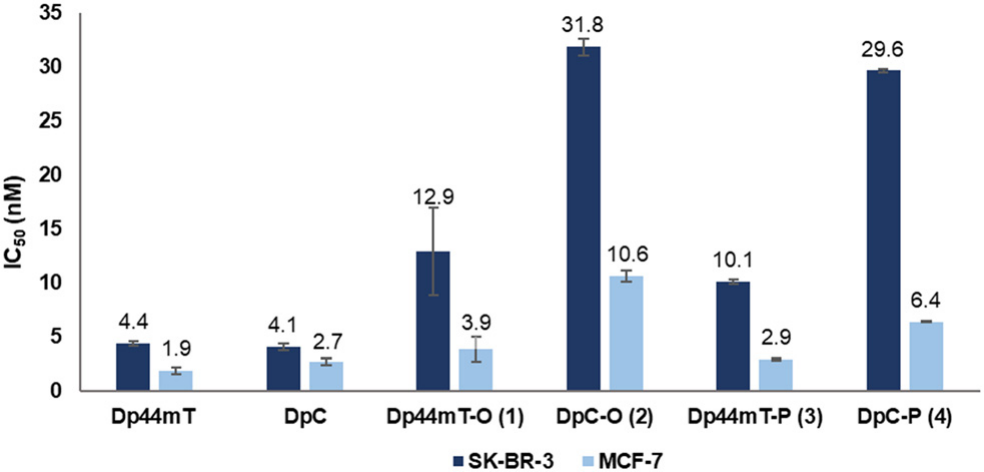
*In vitro* activity of the DpT azides as determined by the MTT assay displayed as IC_50_ (nM) values. SK-BR-3 and MCF-7 cell lines were incubated with the respective compound for 120 h using Dp44mT and DpC as positive controls and 0.5% DMSO as negative control. The values are presented as the mean of three independent experiments ± standard deviation given as error bars. The individual dose–response curves are in the ESI† (Fig. S67 and S68).

Having acquired DpT azides 1–4, we investigated the antiproliferative activities of the synthesized azides 1–4 with Dp44mT and DpC as controls in the MCF-7 and the SK-BR-3 cell lines (Fig. 3), using the standard 3-(4,5-dimethylthiazol-2-yl)-2,5-diphenyltetrazolium bromide (MTT) assay. The breast cancer cell line SK-BR-3 was selected as *in vitro* model due to its well-established overexpression of the HER2 receptor, which would be the target for Tras; MCF-7 breast cancer cells with documented low HER2 expression were used as control.^34–37^ The expected HER2 expression was confirmed by Western blot (see Fig. S72†).^38^ To further validate the difference in HER2 expression between the cell lines, their response towards lapatinib, an FDA-approved drug used to treat HER2-positive breast cancer, was assessed (Fig. S64†).^39^ Lapatinib demonstrated about two orders of magnitude higher activity against SK-BR-3 cells (IC_50_ = 83.7 ± 4.1 nM) than MCF-7 cells (IC_50_ = 6.7 ± 0.4 μM) following a 72 h incubation, indicating that the former overexpressed the HER2 antigen unlike the latter.

For the MTT assay with the DpT azides, the previously reported incubation period of 72 h, established for the parent DpTs, was insufficient to achieve a 100% decrease in cell viability (Fig. S65 and S66†).^40^ Notably, approximately 50% of the SK-BR-3 cells remained viable even at the highest tested concentration, compared to 30% viability in MCF-7 cells. Consequently, we chose a prolonged incubation time of 120 h, which led to an adequate decrease in cell viability in both cell lines (Fig. S67 and S68†).

As illustrated in Fig. 3, both unmodified parent DpTs (*i.e.*, Dp44mT and DpC) were found to be similarly active in the low nanomolar range against both cell lines, with slightly better activity observed against MCF-7 cells. However, the four azide-functionalized DpTs displayed notable differences in cytotoxicity: a 5-to-7-fold decrease in activity was observed in SK-BR-3 compared to MCF-7 for each compound. The cytotoxicity of the Dp44mT-derived compounds 1 and 3 appeared mostly retained against MCF-7 cells, with a slight decrease in activity against SK-BR-3 cells. In contrast, DpC azides 2 and 4 showed notably higher IC_50_ values in both cell lines, especially in SK-BR-3, where the decrease in potency was most pronounced. Interestingly, the position of the linker on the DpT scaffold had no discernible effect on the potency, as all the derivatives showed IC_50_ values comparable to their regioisomers. In general, despite functionalization, all azide-bearing compounds continued to suppress cellular viability in the nanomolar range upon functionalization, and the cytotoxicity was more prominent in MCF-7 cells than in SK-BR-3 cells.

### Metal binding studies

Despite the good activity exhibited by the azides, we were intrigued by the anomalous behavior of the DpC azides in SK-BR-3 cells. As mentioned in the introduction, DpTs exert anticancer activity through multiple pathways involving iron and copper cation chelation.^2^ We hypothesized that the presence of the azide linker and the mixture of *E*/Z isomers might affect the chelating ability of the molecules. Hence, we decided to compare the binding constants of the azides with Fe(ii) and Cu(ii) ions to those of the parent DpTs.

**Fig. 4:**
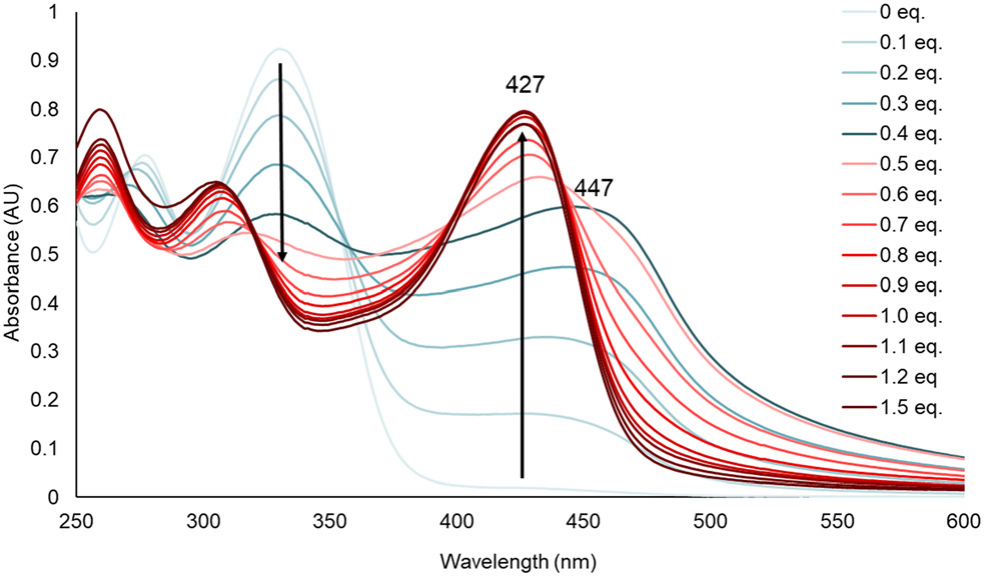
UV/vis absorption profile acquired by titrating CuSO_4_ to 50 μM 4 in 75 mM Tris-HCl buffer at pH 7.4. Absorption maximum shifts from 447 nm at 0.4 equiv. to 427 nm at 1.0 equiv.

We employed UV/vis spectroscopy to monitor the absorption changes upon titrating FeCl_2_ or CuSO_4_ to the respective DpTs. It has been demonstrated that DpTs form 2 : 1 complexes with Fe(ii), which is also consistent with our spectroscopic data, as we observed saturation of absorption after titrating 0.5 equiv. of Fe(ii) (Fig. S53†).^41,42^ The DpT–Cu(ii) complexes, on the other hand, have been isolated in both 1 : 1 and 2 : 1 binding modes using different reaction conditions.^43^ Richardson *et al.* have inferred from EPR data that the Cu(ii) complex prefers the 1 : 1 binding mode in solution. In line with these observations, our titrations with Cu(ii) showed a saturation of absorption after titrating 1.0 equiv. of Cu(ii), indicating a 1 : 1 stoichiometry (Fig. S51†). However, the UV/vis spectra for 3 and 4 initially showed an absorption maximum at 447 nm (Fig. 4 and S51†) at 0.4 equiv. of metal, which shifted to 427 nm as the titration progressed. This shift may indicate that the 2 : 1 complex eventually dissociates into a 1 : 1 complex, suggesting that the binding constant for the latter is higher than that of the former.

Further, the absorption data at *λ*_max_ centered between 400 and 450 nm of the respective charge transfer bands were plotted against the titrated metal concentrations, and the curves were fitted using 1 : 1 and 2 : 1 binding models in Bindfit,^39^ allowing us to determine the respective association constants (Table 1). Interestingly, the log *β*_2_ values for the DpC–Fe(ii) complexes were nearly 3-fold lower than that of the Dp44mT–Fe(ii), suggesting that steric hindrance from the cyclohexyl ring in DpCs might be negatively affecting the binding when forming a 2 : 1 complex. On the other hand, the log *K*_a_ values for all the 1 : 1 Cu(ii) complexes were similar.

**Table 1:** Binding constants are shown as log *β*_2_ for Fe(ii) due to 2 : 1 ligand-to-metal binding and log *K*_a_ for Cu(ii) due to 1 : 1 binding. The binding constants were calculated by monitoring changes to the UV/vis absorption profiles upon titrating the appropriate metal salt to a 50 μM solution of the respective DpT in 75 mM Tris-HCl at pH 7.4. The web app Bindfit was used for curve fitting and acquiring the binding constants^44,45^

	Dp44mT	DpC	1	2	3	4
Fe(ii)L_2_	35.7 ± 0.5	10.9 ± 0.1	37.6 ± 0.1	11.2 ± 0.3	38.7 ± 0.2	11.7 ± 0.3
Cu(ii)L	5.8 ± 0.1	5.4 ± 0.2	6.4 ± 0.3	5.1 ± 0.4	5.8 ± 0.3	5.5 ± 0.7

Clearly, positioning of the linker did not influence metal binding as the derivatives exhibited binding comparable to their corresponding regioisomers. Furthermore, the azide-functionalized DpTs showed binding constants comparable to the unmodified DpTs, indicating that azide-functionalization and the presence of a diastereomeric mixture do not impair chelation.

### Synthesis of ADCs

**Scheme 2:**
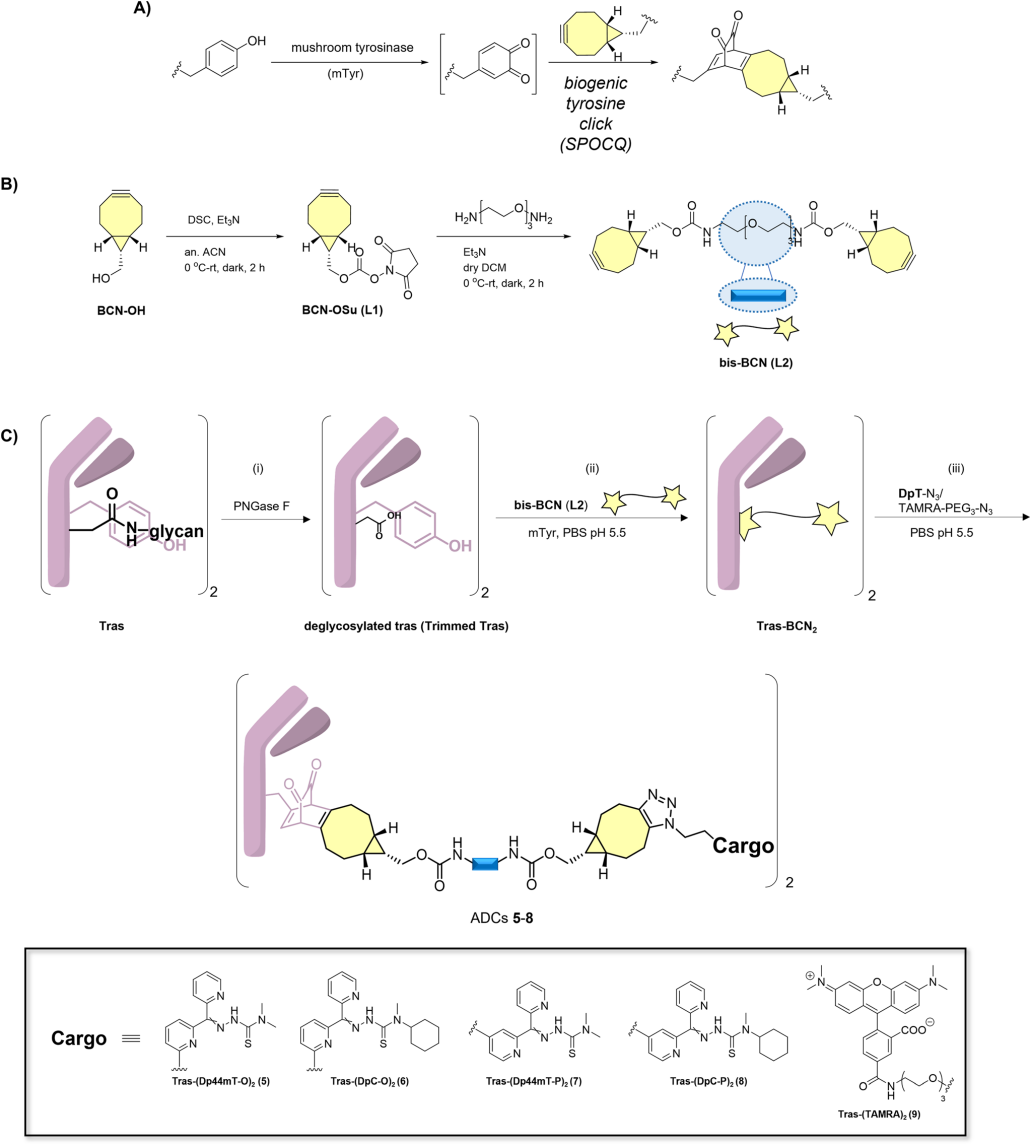
Generation of ADCs 5–8 and TAMRA conjugate 9 from the off-the-shelf antibody trastuzumab. A) A typical enzyme-induced SPOCQ reaction between BCN and *o*-quinone was generated upon oxidation using mushroom tyrosinase (mTyr). B) Synthesis of bis-BCN (**L2**). C) Three-step preparation of ADCs: i) enzymatic deglycosylation of the mAb using PNGase F; ii) SPOCQ reaction on deglycosylated mAb; iii) SPAAC with azide-functionalized DpTs and TAMRA-PEG_3_-azide. See ESI† for full experimental details and characterizing data.

Previous work revealed that removal of the native glycan at Asn297 of the C_H_2 domain of an IgG1 mAb such as trastuzumab exposes two proximal tyrosine residues (Y296 and Y300) that can be used for strain-promoted oxidation-controlled cycloalkyne-quinone (SPOCQ) reaction (Scheme 2A).^32^ Here, we tailor this approach for conjugation to azide-functionalized payloads, *i.e.*, DpTs (1–4). For this, we first attach a bis-bicyclo[6.1.0]non-4-yne (bis-BCN) linker to the antibody, which can then be clicked by means of SPAAC to azide-functionalized toxins. As such, we eliminate the need to synthesize the BCN-variant of each payload, expediting our synthesis.

The synthesis began with an OSu-activation of BCN-OH to form **L1**, after which it was exposed to PEG_3_-bisamine, resulting in the formation of the desired bis-BCN linker **L2** (Scheme 2B) (see ESI† for detailed synthesis schemes). After deglycosylation of Asn297 of Tras using the enzyme peptide-N-glycosidase F (PNGase F) (Scheme 2C), an exposed proximal tyrosine residue in the Fc region becomes available for SPOCQ conjugation. Specifically, mushroom tyrosinase was applied to oxidize a tyrosine into a reactive *o*-quinone, which, in the presence of a strained alkyne, *i.e.*, **L2** at 4 °C for 16 h, led to an inverse electron-demand Diels–Alder cycloaddition. Using **L2**, each heavy chain was equipped with a free BCN handle. Having successfully attached the click handles, Tras-BCN_2_ was treated with one of the different azide-functionalized payloads (1–4) at 4 °C for 16 h, affording the various target ADCs (5–8) at a drug-to-antibody ratio (DAR) of 2. Similarly, TAMRA-PEG_3_-azide was used to conjugate TAMRA, a rhodamine-based fluorophore, to Tras, yielding 9. RP-HPLC-MS and SDS-PAGE confirmed the formation of the desired products (Scheme 2C, Fig. S54–S58†). Using the same method, a cetuximab (Cet) conjugate of Dp44mT-O 1 was synthesized for use as a negative control. (Fig. S59†).

### Uptake studies

To ascertain that functionalizing at the tyrosine residues does not hamper Tras' binding to the HER2 antigen and subsequent intracellular uptake, we synthesized the Tras–(TAMRA)_2_ conjugate 9 (*λ*_exc_/*λ*_em_: 550/575 nm).

**Fig. 5:**
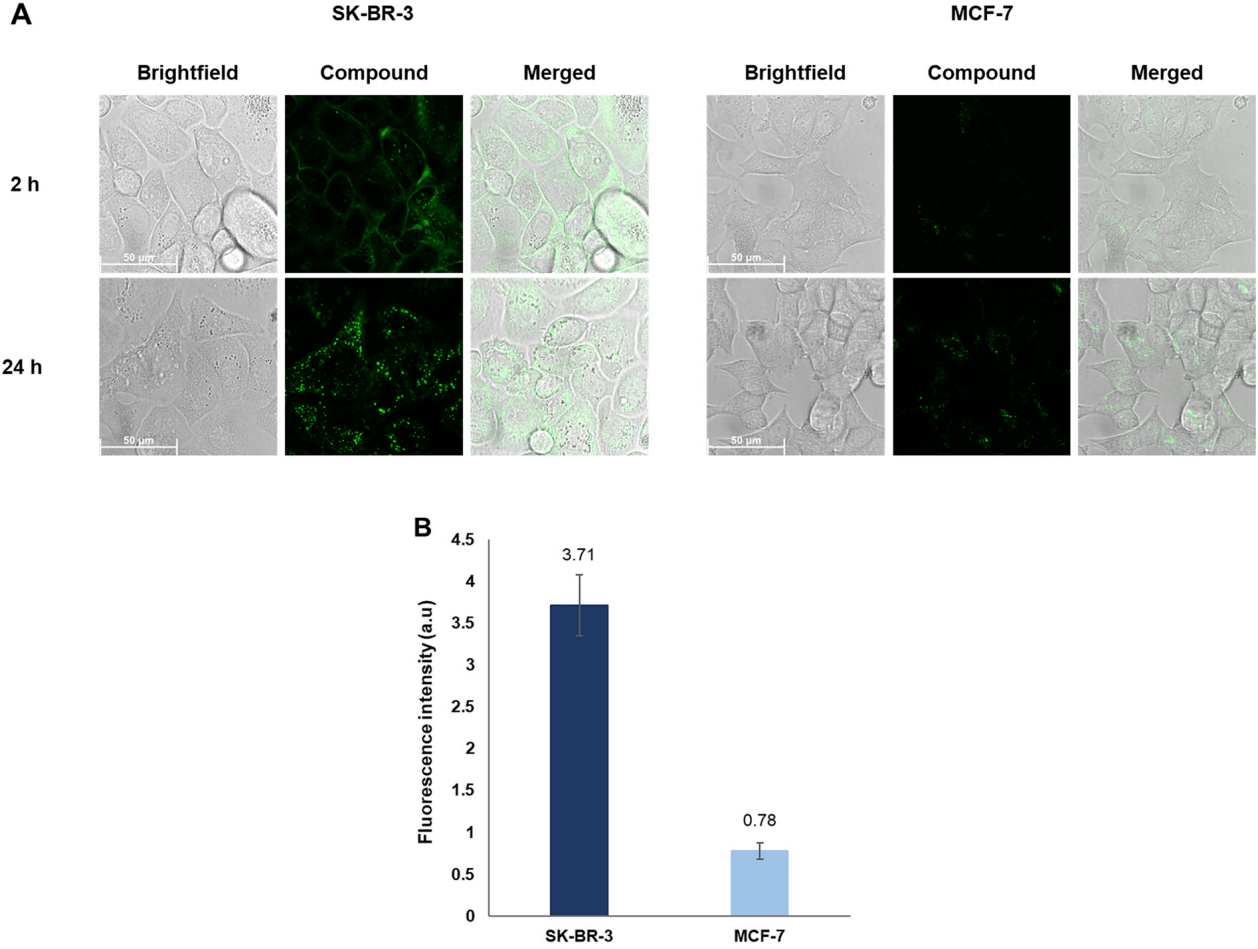
(A) Live-cell images acquired after incubating SK-BR-3 and MCF-7 cells with the Tras–(TAMRA)_2_ conjugate 9 after 2 h and 24 h respectively. Images with untreated control cells can be found in the ESI† (Fig. S73). (B) Fluorescence intensities (brightness) of the images taken after 24 h quantified using ImageJ. The values are presented as the mean of two independent experiments ± standard deviation given as error bars. See ESI† for further experimental details.

SK-BR-3 and MCF-7 cells were incubated with the fluorophore conjugate 9 (2 μM) for 2 h and 24 h, respectively, and observed under a confocal microscope (Fig. 5). The two time points were selected to investigate changes in localization patterns in the respective cell lines over time. In SK-BR-3 cells, after 2 h, the conjugate predominantly localized to the cell membrane, likely through binding to the HER2 receptor, with a few intracellular specks also visible. However, after 24 h, the majority of the fluorescence appeared distributed as spots in the cytoplasm, indicating efficient cellular uptake. This behavior is in line with the mechanism of action of Tras and its ADCs, which exert their anticancer activity in a stepwise manner, initially by binding to the extracellular domain of the HER2 receptor, followed by internalization of the payload *via* endocytosis.^26,46^ On the other hand, with the MCF-7 cells, while the conjugate did not exhibit binding to the cell membrane after 2 h, minimal uptake was observed. After 24 h, the conjugate eventually showed an intracellular localization pattern similar to the SK-BR-3 cells, suggesting internalization. However, a quantitative comparison of the fluorescence intensities between the images acquired after 24 h revealed that the uptake was approximately 5-fold lower in MCF-7 cells than in SK-BR-3 cells. These results corroborate the low levels of HER2 expression previously observed in MCF-7 cells, thereby confirming the selectivity of Tras towards the SK-BR-3 cells. Furthermore, functionalizing Tras at the tyrosine residues using SPOCQ and SPAAC neither had an impact on the mAb's HER2 binding ability nor its ability to get internalized.

### Antiproliferative activity of ADCs

**Fig. 6:**
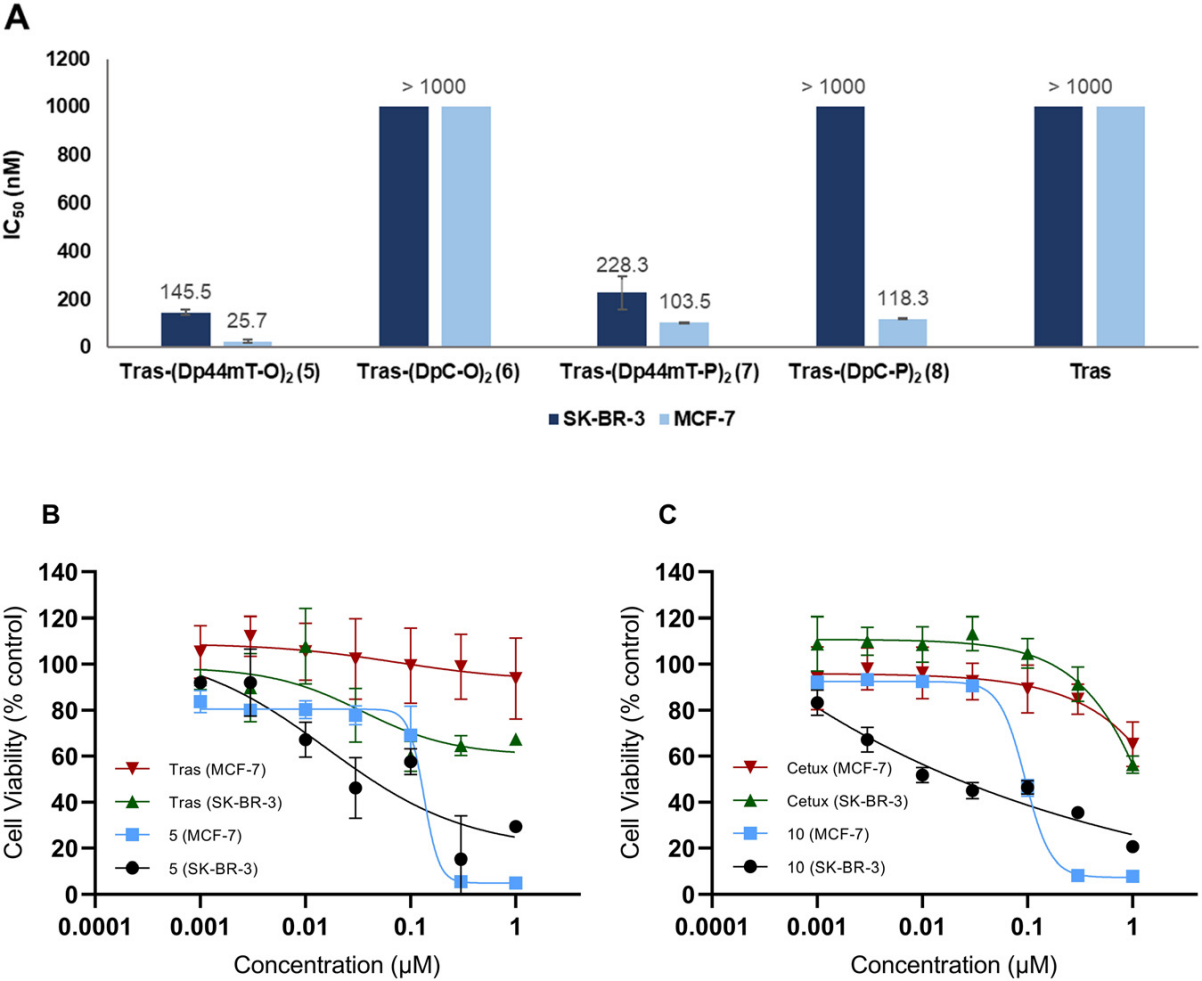
(A) *In vitro* activity of the trastuzumab-based ADCs displayed as IC_50_ (nM) values. SK-BR-3 and MCF-7 cell lines were incubated with compounds 5–8 for 120 h using Tras, trimmed Tras, Dp44mT, and DpC as controls (only Tras included here; see ESI† for the IC_50_ values of remaining controls). The individual dose–response curves can be found in the ESI† (Fig. S69 and S70). (B) Dose–response curve obtained after incubating 5 with SK-BR-3 and MCF-7 cells for 2 hours, followed by swapping with fresh media and incubating for a further 118 h. Tras and Dp44mT (Fig. S71†) were used as controls. (C) Dose–response curves of Cet–Dp44mT-O conjugate 10 against SK-BR-3 and MCF-7 cells. Compounds were incubated for 120 h using cetuximab (Cet) and Dp44mT as controls. Each data point presents the mean of three independent experiments ± standard deviation.

We then tested the ADCs on SK-BR-3 and MCF7 cell lines to assess their antiproliferative activities (Fig. 6A). Tras and its deglycosylated variant (trimmed Tras) were used as controls. From the dose–response curves, the controls were found to be cytotoxic (antibody-dependent cellular cytotoxicity (ADCC)) to the SK-BR-3 cells, even at the lowest concentration (Fig. S69†). However, they were innocuous with the MCF-7 cells, confirming that Tras continued to be selective for HER2-positive cells after deglycosylation.

The ADCs correspondingly displayed trastuzumab-dependent baseline activity at low concentrations in SK-BR-3 cells, which was absent in MCF-7 cells (Fig. S69 and S70†). This supports the notion that the antibody remained conjugated to the payload for the incubation time. Interestingly, MCF-7 cells were generally more sensitive to the ADCs at higher concentrations, consistent with the trend observed for the unconjugated azides (Fig. 3). Specifically, we observed a 6-fold and 2-fold difference in cytotoxicity between the two cell lines for conjugates 5 and 7, respectively (Fig. 6A). While the DpC-based ADC 6 was largely non-cytotoxic to both cell lines, the linker positioning played a crucial role in MCF-7 cells, as 8 reduced cell viability by 8-fold. On the other hand, *ortho* positioning of the linker was more favorable for the Dp44mT-based ADCs as both cell lines were relatively less susceptible to conjugate 7 compared to 5. Overall, these findings suggest that the conjugated DpT payload exerts a greater influence on the *in vitro* activity, overriding the *a priori* expected selectivity offered by trastuzumab. Furthermore, while linker position did not affect the cytotoxicity of the azides, ADCs displayed significant potency differences depending on the linker position.

Intrigued by the findings, we picked ADC Tras-(Dp44mT-O)_2_5, which displayed the highest potency in both cell lines, to perform further experiments. The uptake studies with 9 had indicated that Tras conjugates in SK-BR-3 cells typically bind to the cell membrane within 2 h and then become internalized. In view of this, we hypothesized that the sustained presence of the conjugates during the entire incubation period might be negatively affecting trastuzumab's selectivity for the cell line. To test this, we incubated compound 5 with both cell lines for 2 h, swapped for fresh media, and continued the incubation for an additional 118 hours (Fig. 6B). We presumed that since negligible uptake had been observed after 2 h of incubation of 9 in MCF-7 cells, the activity would be relatively lower. Interestingly, this adjustment did not alter the cytotoxicity profile, as 5 remained more toxic to MCF-7 and failed to minimize the viability of SK-BR-3 cells even at the highest concentrations, underscoring that the observed activity originates from the toxicity of the payload. In accordance with these results, the control Dp44mT's activity also showed a significant reduction in SK-BR-3 cells (Fig. S71†). Additionally, ADCC was observed for Tras at higher concentrations against SK-BR-3 cells, a pattern that was also evident for 5, implying that Tras-dependent activity persisted.

**Fig. 7:**
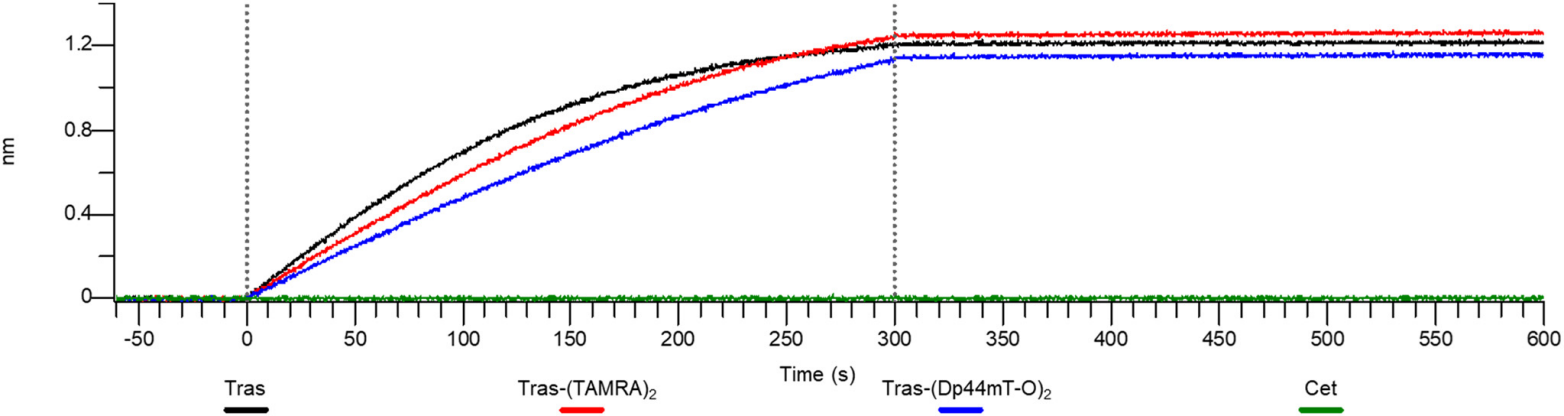
Biolayer interferometry confirms binding of the modified antibodies to the target antigen HER2. Further experimental details can be found in the ESI.†

At this point, one might ask whether modification of the antibody has affected HER2 binding affinity or whether binding is still specific. So, to confirm whether the HER2 binding ability of Tras had been retained after conjugation to DpTs, we performed affinity studies of 5 with the HER2 protein using biolayer interferometry (Fig. 7).^47,48^ Tras and 9 were included as positive controls, and cetuximab (Cet), a mAb that binds the HER1 receptor, was included as the negative control.^49^

The binding of ADC 5 to HER2 protein was observed within the 0–300 s timeframe, closely resembling the binding profiles of the controls Tras and 9. Notably, subsequent washings with PBS did not lead to dissociation, indicating that the constructs retained their binding affinity to HER2. Meanwhile, Cet did not appear to have any affinity for the protein. These results suggest that conjugating DpTs did not alter the HER2 binding ability of the Tras, further supporting the notion that the cell line-specific cytotoxicity of Dp44mT overrides the targeting of Tras.

To further ascertain Dp44mT's prominent role in the activity of the ADCs, we conjugated 1 to Cet. As illustrated in Fig. 6C, the unconjugated control antibody Cet proved non-cytotoxic to both cell lines. Consistent with previous findings, cetuximab conjugate 10 demonstrated a greater cytotoxic effect against MCF-7 cells than SK-BR-3 cells, where it failed to achieve a 100% decrease in cell viability even at the highest concentrations. Notably, we did not observe the Tras-like ADCC against SK-BR-3 cells with Cet, further validating the selectivity of Tras towards this cell line.

## 3. Conclusion

We herein report a facile synthetic route to introduce an azide handle at the *ortho* and *para* positions of one of the pyridyl rings of the thiosemicarbazones Dp44mT and DpC and their subsequent incorporation into antibody–drug conjugates (ADCs) based on trastuzumab. All four azide derivatives demonstrated promising *in vitro* activity against SK-BR-3 and MCF-7 cell lines, with the Dp44mT-based molecules showing slightly greater potency in MCF-7 cells. After installation of a bis-BCN linker on the monoclonal antibody by means of SPOCQ cycloaddition to trastuzumab, azide-functionalized cytotoxic payloads were attached *via* SPAAC, and the antiproliferative activity of the resulting ADCs was assessed in the same two cell lines. Surprisingly, while the DpC-based ADCs were non-cytotoxic to SK-BR-3 cells, the Dp44mT–trastuzumab conjugates exhibited superior activity against MCF-7 cells compared to SK-BR-3 cells. Notably, Tras–Dp44mT-O 5 displayed the highest potency against both cell lines with IC_50_ values of 25.7 ± 5.5 nM and 145.5 ± 11.9 nM against MCF-7 and SK-BR-3, respectively. Unlike the FDA-approved T-DM1 (ref. 22) and our previous trastuzumab–MMAE conjugate,^32^ where the ADCs show a Tras-based selectivity and therefore specifically affected the viability of SK-BR-3 cells, the activity of our ADCs was heavily dependent on the conjugated Dp44mT's intrinsic potency, which superseded the targeting mechanism of trastuzumab. This was further corroborated by conjugating Dp44mT-O to cetuximab, which also demonstrated similar potent nanomolar activity against MCF-7 cells (IC_50_ = 93.7 ± 7.5 nM). Future efforts will focus on investigating the mechanism underlying the superior role of the DpT payload with respect to the antibody in the bioactivity of the conjugates. Considering the potent activity of the DpTs, we are currently investigating alternative methods for delivering them, aiming to enhance targeting and selectivity.

## Author contributions

N. S. conceptualization, investigation, methodology (synthesis of DpTs and characterization, cell tests), formal analysis, visualization, writing – original draft. I. S. investigation, methodology (developed the method and prepared the ADCs), formal analysis, visualization, writing – original draft. A. S. conceptualization, investigation (cell tests), formal analysis, visualization, writing – review & editing. B. A. supervision, funding acquisition, resources, writing – review & editing. N. M. N. supervision, funding acquisition, resources, writing – review & editing.

## Conflicts of interest

B. A. is an inventor on a patent describing the antibody modification chemistry used herein. The other authors declare no conflict of interest.

## Supplementary Material

MD-016-D5MD00154D-s001

## Data Availability

The authors confirm that all data are available as ESI.† Furthermore, additional data and original files are available from the authors upon reasonable request.
