# Supplementary material for: Synthesis and biological evaluation of thiosemicarbazone-based antibody–drug conjugates
Source: RSC Med Chem. 2025 Jun 26;16(8):3512–21. doi: 10.1039/d5md00154d (PMC12198877; doi:10.1039/d5md00154d)
Supplement: MD-016-D5MD00154D-s001 [file MD-016-D5MD00154D-s001.pdf]

Supplementary Information (SI) for RSC Medicinal Chemistry.
This journal is © The Royal Society of Chemistry 2025

Supporting information

MD-RES-02-2025-000154

Synthesis and Biological Evaluation of Thiosemicarbazone-based Antibody-Drug Conjugates

Nandan Sheernaly,[a, †] Irene Shajan,[b, †] Axel Steinbrueck,[a] Bauke Albada,[b] and Nils Metzler-Nolte[*,a]

[a] Faculty of Chemistry and Biochemistry, Inorganic Chemistry I – Bioinorganic Chemistry, Ruhr-University Bochum, Universitaetsstrasse 150, 44801 Bochum, Germany.

[b] Laboratory of Organic Chemistry, Wageningen University & Research, Stippeneng 4, 6708 WE, Wageningen, The Netherlands.

Email address:

nils.metzler-nolte@ruhr-uni-bochum.de

†: These authors contributed equally

Contents

1. List of abbreviations 2
2. General remarks 4
3. Synthesis Procedures 6
4. Spectroscopic information 16
5. Metal binding profiles 42
6. Antibody modifications and bioconjugation 45
7. Cell studies 53
8. References 59

1. List of abbreviations

an.	anhydrous
aq.	aqueous
AcOH	acetic acid
ADC	antibody-drug conjugate
BCN	bis-bicyclo[6.1.0]non-4-yne
calcd	calculated
Cet	cetuximab
COSY	correlation spectroscopy
conc.	concentrated
Cy	cyclohexyl
DAR	drug-to-antibody ratio
DCM	dichloromethane
DMF	*N,N*-dimethylformamide
DMSO	dimethylsulfoxide
Dp44mT	di-2-pyridylketone-4,4-dimethyl-3-thiosemicarbazone
DpC	di-2-pyridylketone-4-cyclohexyl-4-methyl-3-thiosemicarbazone
DpT	di-2-pyridyl-thiosemicarbazone
eq.	equivalent
ESI	electronspray ionisation
Et_2O	diethyl ether
EtOAc	ethyl acetate
EtOH	ethanol
Fc	fragment crystallizable
HRMS	high-resolution mass spectrometry
IC_{50}	half maximal inhibitory concentration
MCF-7	Michigan Cancer Foundation - 7
LC-MS	(high performance) liquid chromatography coupled mass spectrometry

LDA	lithium diisopropylamide
m/z	mass-to-charge ratio
Me	methyl
MeCN	acetonitrile
MeOH	methanol
MTT	3-(4,5-dimethylthiazol-2-yl)-2,5-diphenyltetrazolium bromide
n-BuLi	*n*-butyllithium
NMR	nuclear magnetic resonance spectroscopy
NOESY	nuclear overhauser effect spectroscopy
o/n	overnight
PBS	phosphate buffered saline
PEG	polyethylene glycol
RP-HPLC	reversed-phase high-performance liquid chromatography
rt	room temperature
sat.	saturated
SDS-PAGE	sodium dodecyl-sulfate polyacrylamide gel electrophoresis
TEA	triethylamine
TFA	trifluoroacetic acid
THF	tetrahydrofuran
TLC	thin layered chromatography
TMS	tetramethylsilane
Tras	trastuzumab
UV	ultra-violet
δ	chemical shift

2. General remarks

Moisture sensitive reactions

All moisture-sensitive reactions were carried out in a heated glass apparatus and under a protective argon atmosphere. To ensure a constant protective atmosphere, conventional Schlenk techniques were employed: the apparatuses were sealed with rubber septa, and rubber balloons filled with argon were placed. Solvents and liquid reagents were added through a septum via argon-flushed disposable syringes and cannulas, while solids were introduced into the reaction under the counterflow of argon.

Solvents and Reagents

Reagents have been sourced from reputable suppliers at the highest commercially available quality and used without further purification unless specified otherwise. 2-bromo-6-methylpyridine, 4-bromo-6-methylpyridine, di(pyridin-2-yl)methanone, and lapatinib were purchased from BLD-Pharm. *Endo*-bicyclo[6.1.0]non-4-yn-9-ol (BCN-OH) was procured from Synaffix B.V. (Oss, the Netherlands). Mushroom tyrosinase was purchased from Sigma-Aldrich. Trastuzumab (Herzuma) and cetuximab (Erbitux) were obtained from the pharmacy. PNGase F was obtained from New England Biolabs (NEB). FabRICATOR® was purchased from Genovis AB, Sweden. TAMRA-PEG$_3$-azide was purchased from Broadpharm®. Dry solvents (THF, DCM) were taken from a solvent drying system from MBraun (SPS-800) and stored over molecular sieves (3 Å) under argon. DMF was dried by storing it over molecular sieves (3 Å). Solvents for column chromatography and moisture-insensitive reactions (MeOH, DCM, EtOAc, heptane, isopropanol) were sourced of analytical grade and used without further purification.

Thin film and column chromatography

The reaction progress was controlled by thin-film chromatography (TLC) on silica gel-coated aluminum plates (Merck, silica gel 60, F254). As TLC staining reagents, potassium permanganate solution (1.0 g $KMnO_4$, 5 g K_2CO_3, 2 mL 5% (w/v%) aqueous NaOH solution, 150 mL H_2O) was used. For column chromatographic purification of the reaction product, glass columns packed with silica gel (Merck, 60, particle size 0.043 – 0.063 mm) were used as a stationary phase. As the mobile phase, eluent mixtures of solvents (heptane, acetone, DCM, MeOH, EtOAc) were used; the specific compositions are indicated in the individual protocols.

NMR spectroscopy

The NMR spectroscopic examinations of the synthesized substances with respect to their ^{1}H and ^{13}C nuclei were carried out on the Bruker devices drx-400 or neo-400 (400 MHz) or AV-400 or aviii-300 (300 MHz). Chloroform ($CDCl_3$), DMSO (DMSO-d$_6$), methanol (MeOD-d$_4$), and D_2O from Deutero GmbH were used as NMR solvents. The recorded spectra were calibrated relative to the residual solvent reference peak in addition to TMS being employed as the internal standard– ($CDCl_3$: δ (^{1}H) = 7.26 ppm and δ (^{13}C) = 77.16 ppm; DMSO-d$_6$: δ (^{1}H) = 2.50 ppm and δ (^{13}C) = 39.52 ppm; MeOD-d$_4$: δ (^{1}H) = 3.31 ppm and δ (^{13}C) = 49.00 ppm; D_2O: δ (^{1}H) = 4.79 ppm). The program MestreNova 14.0 was used to process the spectra. The following abbreviations were used to describe signal multiplicity: s = singlet, d = doublet, t = triplet, q = quartet, qt = quintet, st = sextet, m = multiplet, and appropriate combinations thereof. The chemical shifts are expressed in parts per million (ppm), and the absolute values of coupling constants *J* are given in Hertz (Hz).

Mass spectrometry (HRMS)

High-resolution mass spectra of the prepared small molecules (m/z < 2000) were recorded on a Vion IMS QToF instrument (Waters) with an ESI in positive sensitivity mode. Masses in a range of 50–2000 *m*/*z* were detected with 0.2 s per scan and leucine enkephalin being injected as a reference mass every minute. The used parameters are as follows: capillary voltage: 0.8 kV, sample cone voltage: 40 V, source offset voltage: 80 V, cone gas flow: 50 L/h, desolvation gas flow: 1000 L/h, source temperature: 120°C, desolvation temperature: 550°C, collision gas: N_2, collision low energy: 6 V, and collision high-energy ramp: 28–60 V.

High-resolution mass spectrometry analysis of ADC derivatives was performed with a Q-Exactive Focus Mass Spectrometer (Thermo Fisher) equipped with an ESI in positive mode. RP-HPLC analysis was performed on an Agilent 1290 series instrument. Deconvoluted spectra were obtained using UniDec software.[1]

General bioconjugation procedures

NAb™ Protein A Plus Spin Columns (1 mL or 0.2 mL) from Thermo Fisher Scientific were used for protein A purification of the samples. Amicon® Ultra Centrifugal Filters, 50 kDa MWCO from Millipore were used for spin filtration. SDS-PAGE gels were scanned in BIO-RAD ChemDoc XRS+ Imager. Protein concentration was determined using NanoDrop 2000c (Thermo Scientific) spectrophotometer.

General procedure for reducing SDS-PAGE, Coomassie staining, and fluorescence detection

12% acrylamide gels were prepared according to BIO-RAD bulletin 6201 protocol. 5 µg of the antibody solution was diluted in 5 µL PBS (pH 7.4). 5 µL of 2× sample buffer was added to the sample and heated to 95 °C for 5 minutes. After loading the samples, the gel was run using a BIO-RAD Mini-PROTEAN Tetra Vertical Electrophoresis Cell at 120 volts until completion. The gel was stained using a 1 g/L Coomassie Brilliant Blue R-250 in 5:4:1 (v/v/v) methanol:water:acetic acid solution (30 minutes soak). The gel was subsequently destained using 5:4:1 (v/v/v) methanol:water:acetic acid for 30 minutes, after which it was further destained overnight using demineralized water.

General procedure for analytical RP-HPLC-MS

Prior to RP-HPLC analysis, 10 µg of IgG sample in PBS 7.4 (total volume of 30 µL) was incubated with 26.6 units (0.4 µL) of FabRICATOR at 37 °C for 2 h. RP-HPLC analysis was performed on an Agilent 1290 series instrument. The sample (20 µL) was injected with 0.6 mL/min onto MAbPac RP 3.0 × 100 mm, 4 µm (Thermo Scientific) with a column temperature of 80 °C. A linear gradient was applied in 15 minutes from 25% to 40% acetonitrile with 0.1% FA and water with 0.1% FA.

BLI studies

The measurement was done on an OCTET RED96E instrument. Octet® High Precision Streptavidin (SAX) Biosensor was purchased from Sartorius. Biotinylated HER2 (Her2/ERBB2 Protein, Human, Recombinant (His & AVI Tag), Biotinylated, (catalog number: 10004-H27H-B) was purchased from Sino Biological.

3. Synthesis Procedures

i) Synthesis of DpTs

Synthesis of O1 and P1[2]

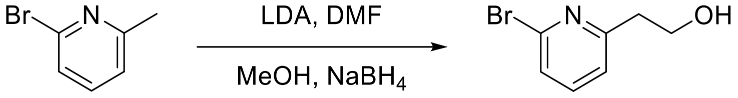

R1 **O1**

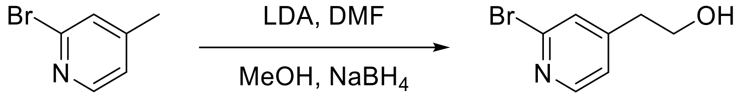

R2 **P1** *

A solution of diisopropylamine (1.5 eq.) in dry THF (1 mL per mmol substrate) was cooled to -40°C, and *n*-BuLi (1.6 M solution in hexanes) (1.6 eq.) was added dropwise. The reaction was then warmed to 0°C, stirred for 30 min, and cooled to -78 °C. Subsequently, a solution of the appropriate bromomethylpyridine (1.0 eq) in dry THF (0.33 mL/mmol) was added dropwise to the prepared LDA solution. The resulting reaction mixture was stirred at -78°C for 30 min. Afterward, a solution of dimethyl formamide (1.05 eq.) in THF (0.17 mL/mmol) was added dropwise, and the reaction was stirred at -78 °C for an additional 30 min. Finally, acetic acid (1.1 eq.), methanol (1 mL per mmol substrate), and sodium borohydride (0.95 eq.) were added, and the reaction was allowed to warm up to rt and stirred overnight. The reaction was then quenched with sat. aq. $NaHCO_3$ solution and the aqueous phase was extracted with EtOAc (4 x 50 mL). The combined organic phases were washed with water and brine, dried over an. Na_2SO_4, filtered and concentrated under reduced pressure. The crude residue was later purified via silica gel column chromatography (Hexane:EtOAc (9:1 → 1:1) v/v) to obtain the desired product as a yellow oil.

2-(6-bromopyridin-2-yl)ethan-1-ol (O1): Yield: 62 % (11.00 g from 15.00 g of **R1**)

^{1}H NMR (400 MHz, $CDCl_3$): δ (ppm): 7.48 (t, *J* = 7.7 Hz, 1H), 7.35 (dd, *J* = 7.9, 0.8 Hz, 1H), 7.14 (dd, *J* = 7.5, 0.9 Hz, 1H), 4.02 (q, *J* = 5.8 Hz, 2H), 3.01 (t, *J* = 5.7 Hz, 2H), 2.95 (t, *J* = 6.0 Hz, 1H).

^{13}C NMR (101 MHz, $CDCl_3$): δ (ppm): 161.8, 141.6, 138.9, 126.0, 122.5, 61.7, 39.5.

2-(2-bromopyridin-4-yl)ethan-1-ol (P1): Yield: 62% (7.91 g from 10.85 g of **R2**)

^{1}H NMR (400 MHz, $CDCl_3$): δ (ppm): 8.25 (d, *J* = 5.0 Hz, 1H), 7.39 (d, *J* = 1.5 Hz, 1H), 7.14 (dd, *J* = 5.0, 1.5 Hz, 1H), 3.91 (q, *J* = 5.6 Hz, 2H), 2.84 (t, *J* = 6.3 Hz, 2H), 1.76 – 1.68 (m, 1H).

^{13}C NMR (101 MHz, $CDCl_3$): δ (ppm): 151.5, 150.1, 142.6, 128.8, 123.7, 62.3, 38.1.

Synthesis of O2 and P2[2]

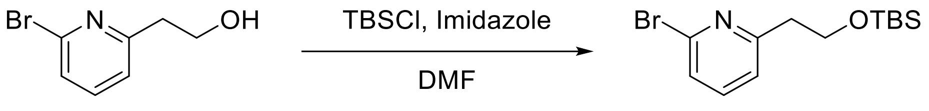

O1 **O2**

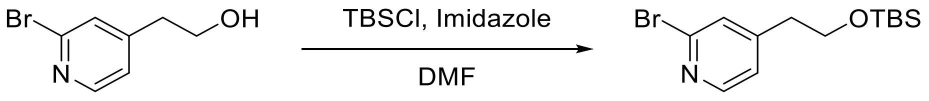

P1 **P2**

After dissolving the respective starting materials (**O1**/**P1**) (1.00 eq.) in DMF (1 mL per mmol substrate), imidazole (5.00 eq.) and *tert*-butyldimethylsilylchloride (1.02 eq.) were added. The resulting reaction mixture was stirred overnight at room temperature. Subsequently, the reaction was diluted with water, and the aqueous phase was extracted with EtOAc (3 x 50 mL). The combined organic layer was washed with water and brine, dried over an. Na_2SO_4, filtered and concentrated under reduced pressure. The crude residue was later purified via silica gel column chromatography (hexane:EtOAc (9:1 v/v)) to obtain the desired product as a pale yellow oil, which later solidified into a pale yellow substance.

2-bromo-6-(2-((tert-butyldimethylsilyl)oxy)ethyl)pyridine (O2): Yield: 88% (15.30 g from 11.00 g of **O1**)

^{1}H NMR (400 MHz, $CDCl_3$): δ (ppm): 7.43 (t, *J* = 7.7 Hz, 1H), 7.31 (dd, *J* = 7.9, 1.0 Hz, 1H), 7.16 (dd, *J* = 7.5, 1.0 Hz, 1H), 3.96 (t, *J* = 6.2 Hz, 2H), 2.95 (t, *J* = 6.2 Hz, 2H), 0.82 (s, 9H), -0.05 (s, 6H).

^{13}C NMR (101 MHz, $CDCl_3$): δ (ppm): 161.6, 141.6, 138.4, 125.7, 123.1, 62.6, 41.4, 26.0, 18.4, -5.4.

2-bromo-4-(2-((*tert*-butyldimethylsilyl)oxy)ethyl)pyridine (P2): Yield: 83% (10.30 g from 7.89 g of **P1**)

^{1}H NMR (400 MHz, $CDCl_3$): δ (ppm): 8.25 (d, *J* = 5.0 Hz, 1H), 7.37 (s, 1H), 7.11 (d, *J* = 5.0 Hz, 1H), 3.82 (td, *J* = 6.3, 1.3 Hz, 2H), 2.77 (t, *J* = 6.2 Hz, 2H), 0.86 (s, 9H), -0.02 (m, 6H).

^{13}C NMR (101 MHz, $CDCl_3$): δ (ppm): 152.0, 149.8, 142.3, 129.0, 123.9, 62.8, 38.4, 26.0, 18.4, -5.4.

Synthesis of O3 and P3[3]

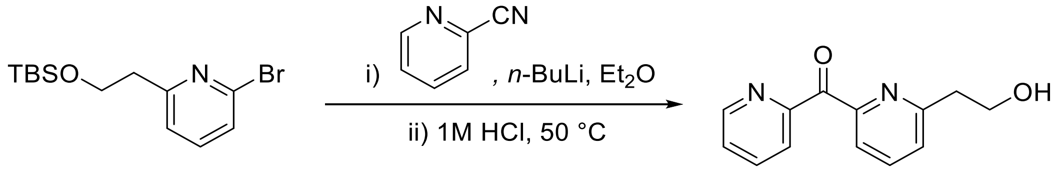

O2 **O3**

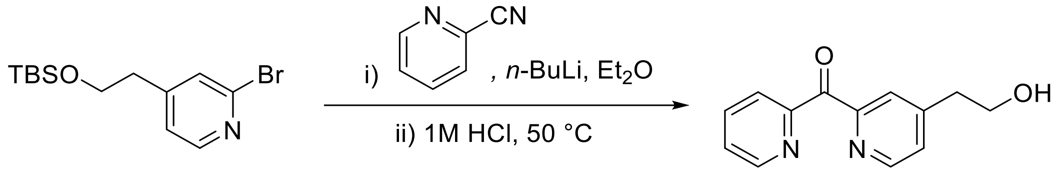

P2 **P3**

The respective starting material (**O2**/**P2**) (1.00 eq.) was dissolved in dry diethyl ether (2 mL per mmol of substrate) and cooled to -78°C before adding *n*-BuLi (1.6 M solution in hexanes) (1.2 eq.) dropwise. The reaction mixture was warmed to -50 °C and stirred for 30 minutes. Subsequently, the reaction was cooled back to -78 °C, and a solution of 2-cyanopyridine (1.1 eq.) in dry diethyl ether (1.25 mL per mmol substrate) was added dropwise. The resulting mixture was stirred for 30 min at -78 °C. The reaction was then poured over ice, after which the mixture attained an orange coloration. The organic phase was extracted thrice with 1M HCl, and the combined aqueous solutions were stirred at 50°C for one hour. Afterward, the aqueous mixture was neutralized with Na_2CO_3 and extracted thrice with EtOAc. The combined organic phases were washed with water and brine, dried over an. Na_2SO_4, filtered and concentrated under reduced pressure. The crude residue was later purified via silica gel column chromatography (hexane:acetone (9:1) → (1:1) v/v) to obtain the desired product as a brown-colored oil.

(6-(2-hydroxyethyl)pyridin-2-yl)(pyridin-2-yl)methanone (O3): Yield: 42% (4.65 g from 15.30 g of **O2**)

^{1}H NMR (400 MHz, $CDCl_3$): δ (ppm): 8.69 – 8.63 (m, 1H), 8.02 (dt, *J* = 7.9, 1.1 Hz, 1H), 7.93 (d, *J* = 7.7 Hz, 1H), 7.84 (td, *J* = 7.8, 1.7 Hz, 1H), 7.77 (t, *J* = 7.8 Hz, 1H), 7.42 (ddd, *J* = 7.7, 4.8, 1.2 Hz, 1H), 7.35 – 7.26 (m, 2H), 4.51 (s, 1H), 3.94 (d, *J* = 6.0 Hz, 2H), 3.01 (t, *J* = 5.3 Hz, 2H).

^{13}C NMR (101 MHz, $CDCl_3$): δ (ppm): 192.0, 159.8, 154.5, 153.0, 148.9, 137.4, 136.9, 126.5, 126.3, 124.9, 122.6, 77.3, 77.0, 76.7, 60.9, 38.3.

(4-(2-hydroxyethyl)pyridin-2-yl)(pyridin-2-yl)methanone (P3): Yield: 20% (0.70 g from 4.80 g of **P2**)

^{1}H NMR (400 MHz, $CDCl_3$): δ (ppm): δ 8.73 (d, *J* = 4.7 Hz, 1H), 8.63 (d, *J* = 5.0 Hz, 1H), 8.08 (d, *J* = 7.8 Hz, 1H), 7.95 (d, *J* = 1.6 Hz, 1H), 7.89 (td, *J* = 7.7, 1.8 Hz, 1H), 7.52 – 7.44 (m, 1H), 7.37 (dd, *J* = 4.9, 1.8 Hz, 1H), 3.92 (t, *J* = 6.4 Hz, 2H), 2.95 (t, *J* = 6.4 Hz, 2H), 2.16 (s, 1H).

^{13}C NMR (101 MHz, $CDCl_3$): δ (ppm): 193.7, 154.9, 154.8, 149.7, 149.6, 149.6, 137.3, 127.7, 126.9, 126.2, 125.7, 77.8, 77.5, 77.2, 62.7, 38.9, 31.4.

Synthesis of O4 and P4

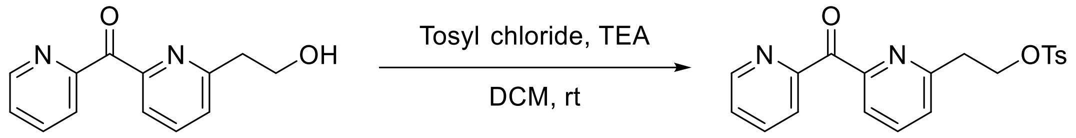

O3 **O4**

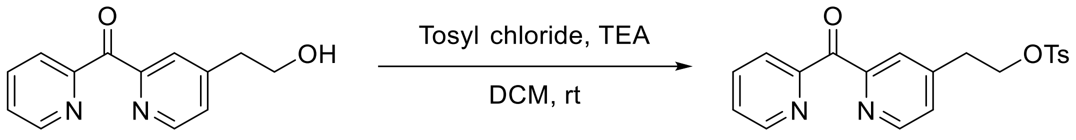

P3 **P4**

O3/P3 (1.0 eq.) was dissolved in DCM (2 mL per mmol substrate). After cooling the solution to 0°C, TEA (2.5 eq.) and tosyl chloride (1.2 eq.) were added. The reaction was then warmed to room temperature and stirred for 3 h. Subsequently, the reaction was quenched with water, and the aqueous phase was extracted twice using DCM. The combined organic phase was washed with water and brine, dried over an. Na_2SO_4, filtered and concentrated under reduced pressure. The crude residue was later purified via silica gel column chromatography (Hexane:EtOAc (9:1) → (1:9) v/v) to obtain the desired product as a pale yellow oil.

2-(6-picolinoylpyridin-2-yl)ethyl 4-methylbenzenesulfonate (O4): Yield: 83% (6.51 g from 4.65 g of **O3**)

^{1}H NMR (400 MHz, $CDCl_3$): δ (ppm): 8.70 (dt, *J* = 4.9, 1.2 Hz, 1H), 8.06 – 7.99 (m, 1H), 7.89 (dd, *J* = 7.8, 1.0 Hz, 1H), 7.85 (td, *J* = 7.7, 1.8 Hz, 1H), 7.75 (t, *J* = 7.7 Hz, 1H), 7.66 – 7.58 (m, 2H), 7.45 (ddd, *J* = 7.6, 4.7, 1.3 Hz, 1H), 7.34 – 7.28 (m, 1H), 7.22 (d, *J* = 8.0 Hz, 2H), 4.40 (t, *J* = 6.4 Hz, 2H), 3.16 (t, *J* = 6.4 Hz, 2H), 2.37 (s, 3H).

^{13}C NMR (101 MHz, $CDCl_3$): δ (ppm): 192.6, 156.4, 154.0, 149.3, 144.8, 137.1, 136.6, 132.9, 129.9, 127.8, 126.6, 126.4, 125.7, 123.5, 69.2, 37.2, 21.6.

2-(2-picolinoylpyridin-4-yl)ethyl 4-methylbenzenesulfonate (P4): Yield: 59% (0.64 g from 0.65 g of **P3**)

^{1}H NMR (400 MHz, $CDCl_3$): δ (ppm): 8.75 (d, *J* = 4.8 Hz, 1H), 8.60 (d, *J* = 5.0 Hz, 1H), 8.09 (d, *J* = 7.8 Hz, 1H), 7.88 (td, *J* = 7.7, 1.7 Hz, 1H), 7.83 (d, *J* = 1.8 Hz, 1H), 7.71 (d, *J* = 8.1 Hz, 2H), 7.47 (ddd, *J* = 6.2, 5.0, 1.3 Hz, 1H), 7.29 (d, *J* = 8.2 Hz, 2H), 4.33 (t, *J* = 6.5 Hz, 2H), 3.07 (t, *J* = 6.5 Hz, 2H), 2.42 (s, 3H).

^{13}C NMR (101 MHz, $CDCl_3$): δ (ppm): 193.0, 154.8, 154.4, 149.5, 149.4, 146.7, 145.2, 136.9, 132.7, 130.1, 128.0, 126.9, 126.6, 125.6, 125.4, 68.8, 34.8, 21.8.

Synthesis of O5 and P5

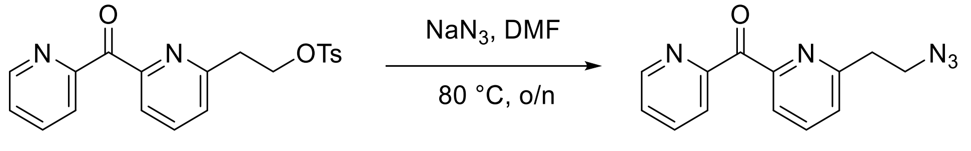

O4 **O5**

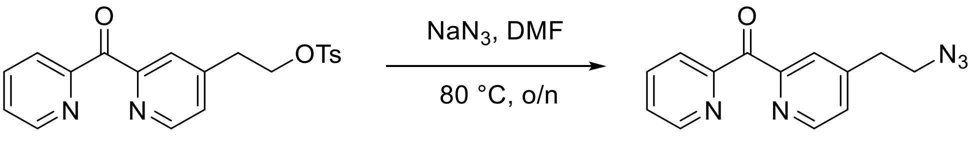

P4 **P5**

O4/P4 (1.00 eq.) was dissolved in DMF (1 mL per mmol substrate), and NaN_3 (5.0 eq.) was added portion-wise. The reaction was then stirred at 80 °C overnight. Subsequently, the reaction was diluted with water, and the resulting aqueous phase was extracted with EtOAc (3x). The combined organic phases were washed with water and brine, dried over an. Na_2SO_4, filtered and concentrated under reduced pressure. The product, obtained as a brown solid, was used for the next reaction without further purification.

(**6-(2-azidoethyl)pyridin-2-yl)(pyridin-2-yl)methanone (O5):** Yield: 98% (4.15 g from 6.46 g of **O4**)

^{1}H NMR (400 MHz, $CDCl_3$): δ (ppm): 8.74 (ddd, *J* = 4.9, 1.9, 1.0 Hz, 1H), 8.09 (dt, *J* = 7.8, 1.1 Hz, 1H), 7.95 (d, *J* = 7.7 Hz, 1H), 7.89 (td, *J* = 7.7, 1.7 Hz, 1H), 7.82 (t, *J* = 7.7 Hz, 1H), 7.48 (ddd, *J* = 7.6, 4.7, 1.2 Hz, 1H), 7.39 (d, *J* = 7.7 Hz, 1H), 3.70 (t, *J* = 6.9 Hz, 2H), 3.12 (t, *J* = 6.9 Hz, 2H).

^{13}C NMR (101 MHz, $CDCl_3$): δ (ppm): 192.8, 157.6, 154.2, 154.0, 149.0, 136.9, 136.4, 126.1, 126.0, 125.2, 123.2, 50.1, 37.0.

(4-(2-azidoethyl)pyridin-2-yl)(pyridin-2-yl)methanone (P5): Yield: 90% (0.36 g from 0.61 g of **P4**)

^{1}H NMR (400 MHz, $CDCl_3$): δ (ppm): 8.76 (dt, *J* = 4.8, 1.3 Hz, 1H), 8.68 (d, *J* = 4.9 Hz, 1H), 8.09 (dt, *J* = 7.9, 1.1 Hz, 1H), 7.97 (d, *J* = 1.7 Hz, 1H), 7.90 (td, *J* = 7.8, 1.7 Hz, 1H), 7.49 (ddd, *J* = 7.7, 4.7, 1.3 Hz, 1H), 7.36 (dd, *J* = 5.0, 1.7 Hz, 1H), 3.63 (t, *J* = 7.0 Hz, 2H), 2.99 (t, *J* = 7.0 Hz, 2H).

^{13}C NMR (101 MHz, $CDCl_3$): δ (ppm): 193.2, 154.9, 154.5, 149.5, 148.3, 136.9, 126.9, 126.6, 125.5, 51.2, 34.8.

Synthesis of M2 and C2[4]

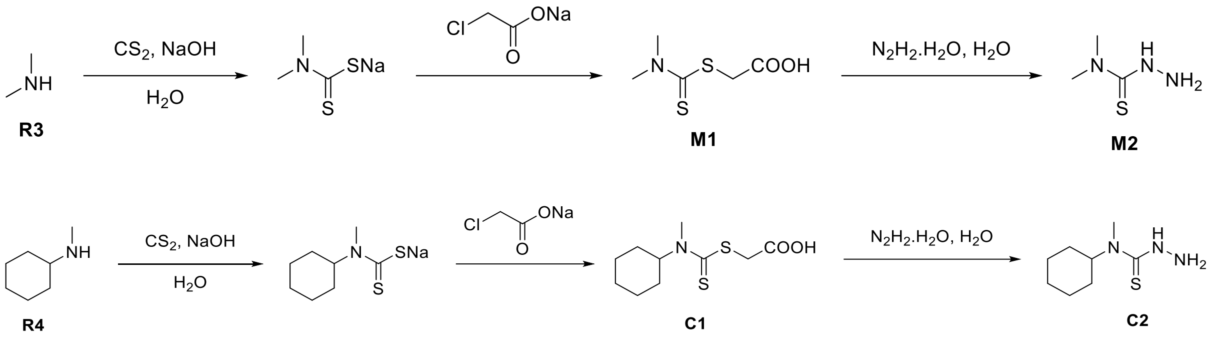

The secondary amine as its hydrochloride salt (**R3/R4**) (1.00 eq.) was dissolved in 0.5 M sodium hydroxide solution (2 mL per mmol substrate) before adding carbon disulfide (1.00 eq.) dropwise. The resulting mixture was stirred vigorously at room temperature for 5 h. The reaction was then transferred to a separation funnel and washed thrice with EtOAc. Subsequently, the aqueous phase was separated, sodium chloroformate (1.03 eq.) was added, and the reaction was stirred overnight at rt. Next, the reaction was acidified with aqueous conc. HCl to pH 1, the resulting white precipitate was filtered, washed with copious amounts of water, and dried under reduced pressure. The crude product was used for the next reaction without further purification.

The carboxylic acid (**M1/C1**) was dissolved in water (0.1 mL per mmol substrate) and hydrazine hydrate (55% in water) (0.3 mL per mmol substrate). The reaction flask was equipped with a reflux condenser and heated with a heat gun until the hydrazine was fuming. The reaction was then allowed to cool down to observe immediate precipitation. The process was repeated 5 times. The colorless precipitate was filtered off, washed several times with ice-cold water, and dried under reduced pressure. The product was used for the next reaction without further purification.

***N,N*-dimethylhydrazinecarbothioamide (M2):** Overall yield: 59% (6.48 g from 15 g **R3**)

^{1}H NMR (400 MHz, DMSO-d$_6$): δ (ppm): 8.63 (br-s, 1H), 4.59 (br-s, 2H), 3.10 (s, 6H).

^{13}C NMR (101 MHz, DMSO-d6): δ (ppm): 182.8, 40.1.

***N*-cyclohexyl-*N*-methylhydrazinecarbothioamide (C2)**: Overall Yield: 55% (2.03 g from 5 g of **R4**)

^{1}H NMR (400 MHz, CDCl$_3$): δ (ppm): 7.26 (s, 1H), 4.82 (d, *J* = 12.2 Hz, 1H), 4.37 (s, 2H), 2.86 (d, *J* = 1.3 Hz, 3H), 1.85 – 1.65 (m, 4H), 1.63 – 1.49 (m, 1H), 1.32 (q, *J* = 9.7, 8.2 Hz, 4H), 1.01 (qt, *J* = 12.4, 3.7 Hz, 1H).

^{13}C NMR (101 MHz, CDCl$_3$): δ (ppm): 184.4, 59.8, 31.2, 30.0, 25.5.

General procedure for the synthesis of Dipyridyl thiosemicarbazones[4]

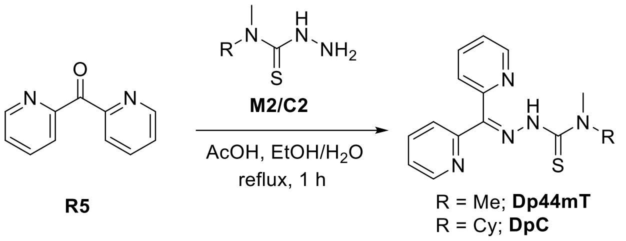

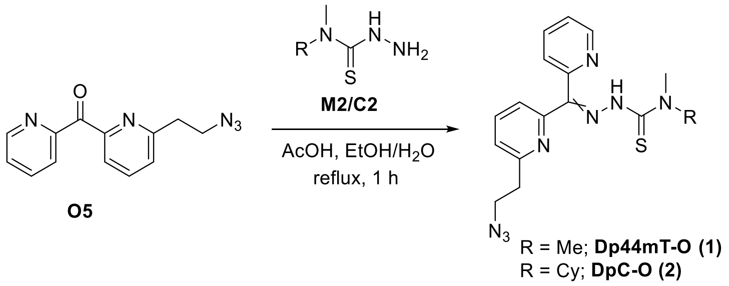

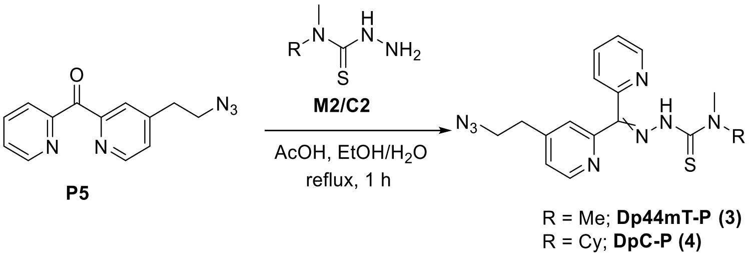

R5/O5/P5 (1.00 eq.) was dissolved in a 1:1 mixture of water (1.5 mL per mmol substrate) and ethanol (1.5 mL per mmol substrate), and **M2/C2** (1.00 eq.) was added. After adding 5 drops of acetic acid, the reaction was refluxed for one hour, while an intense yellow coloration formed over time. The reaction was allowed to cool down to room temperature and diluted with EtOAc and water. The aqueous and organic phases were separated, and the aqueous phase was extracted twice with EtOAc. The combined organic phases were washed with water and brine, dried over an. Na_2SO_4, filtered and concentrated under reduced pressure. The crude residue was later purified via silica gel column chromatography (DCM:MeOH (9.5:0.5)) to obtain the desired product as a yellow solid.

2-(di(pyridin-2-yl)methylene)-*N*,*N*-dimethylhydrazine-1-carbothioamide (Dp44mT): Yield: 70% (1.08 g from 1.00 g of **R5**)

^{1}H NMR (300 MHz, $CDCl_3$): δ (ppm): 15.13 (s, 1H), 8.69 (ddd, *J* = 5.0, 1.8, 0.9 Hz, 1H), 8.57 (ddd, *J* = 4.9, 1.8, 0.9 Hz, 1H), 8.12 (dt, *J* = 7.9, 1.1 Hz, 1H), 7.86 – 7.77 (m, 2H), 7.72 (dt, *J* = 8.2, 1.1 Hz, 1H), 7.37 (ddd, *J* = 7.5, 5.0, 1.3 Hz, 1H), 7.30 (ddd, *J* = 7.6, 4.9, 1.2 Hz, 1H), 3.47 (s, 6H).

***N*-cyclohexyl-2-(di(pyridin-2-yl)methylene)-*N*-methylhydrazine-1-carbothioamide (DpC):** Yield: 25% (0.29 g from 0.62 g of **R5**)

^{1}H NMR (300 MHz, $CDCl_3$): δ (ppm): 14.98 (s, 1H), 8.69 (ddd, *J* = 4.9, 1.8, 0.9 Hz, 1H), 8.57 (ddd, *J* = 4.8, 1.8, 0.9 Hz, 1H), 8.16 (dt, *J* = 8.0, 1.1 Hz, 1H), 7.82 (tt, *J* = 7.7, 2.2 Hz, 2H), 7.73 (dt, *J* = 8.2, 1.2 Hz, 1H), 7.37 (ddd, *J* = 7.5, 4.9, 1.3 Hz, 1H), 7.30 (ddd, *J* = 7.5, 4.9, 1.2 Hz, 1H), 3.25 (s, 3H), 1.93 – 1.83 (m, 4H), 1.72 (d, *J* = 13.2 Hz, 1H), 1.60 – 1.35 (m, 4H), 1.20 – 1.02 (m, 1H).

2-((6-(2-azidoethyl)pyridin-2-yl)(pyridin-2-yl)methylene)-*N,N*-dimethylhydrazine-1-carbothioamide (Dp44mT-O): Yield: 95% (1.33 g from 1.00 g of **O5**); Mixture of *E*/*Z* isomers in the ratio **5:4**; **HRMS**: *m*/*z* found for $C_{16}H_{19}N_8S$ $[M+H]^+$: 355.1456 (calcd m/z: 355.1453)

^{1}H NMR (400 MHz, CDCl$_3$): Major diastereomer (*E*): δ (ppm): 15.20 (s, 1H), 8.69 (d, *J* = 4.7 Hz, 1H), 7.93 (d, *J* = 7.8 Hz, 1H), 7.84 – 7.78 (m, 1H), 7.75 (td, *J* = 7.9, 3.6 Hz, 1H), 7.67 (d, *J* = 8.3 Hz, 1H), , 7.37 (ddd, *J* = 7.6, 4.9, 1.2 Hz, 1H), 7.20 (d, *J* = 7.7 Hz, 1H), 3.67 (t, *J* = 6.8 Hz, 2H), 3.47 (s, 6H), 3.03 (t, *J* = 6.8 Hz, 2H). Minor diastereomer (*Z*): δ (ppm): 14.38 (s, 1H), 8.57 (d, *J* = 4.7 Hz, 1H), 7.97 (d, *J* = 7.9 Hz, 1H), 7.84 – 7.78 (m, 1H), 7.75 (td, *J* = 7.9, 3.6 Hz, 1H), 7.46 (d, *J* = 8.0 Hz, 1H), 7.31 (ddd, J = 7.6, 4.9, 1.2 Hz, 1H), 7.28 (d, *J* = 7.8 Hz, 1H), 3.85 (t, *J* = 7.0 Hz, 2H), 3.14 (t, *J* = 7.0 Hz, 2H).

^{13}C NMR (101 MHz, CDCl$_3$): Mixture of *E*/*Z* isomers: δ (ppm): 181.7, 181.3, 156.5, 156.5, 156.2, 152.2, 151.7, 148.4, 147.1, 143.0, 142.1, 137.8, 137.7, 137.1, 127.1, 125.2, 124.2, 123.9, 123.4, 123.2, 122.9, 50.6, 50.6, 43.0, 41.9, 37.6, 37.1.

2-((6-(2-azidoethyl)pyridin-2-yl)(pyridin-2-yl)methylene)-*N*-cyclohexyl-*N*-methylhydrazine-1-carbothioamide (DpC-O): Yield: 87% (0.29 g from 0.20 g of **O5**); Mixture of *E*/*Z* isomers in the ratio **3:2**; **HRMS**: *m*/*z* found for $C_{21}H_{27}N_8S$ $[M+H]^+$: 423.2079 (calcd m/z: 423.2079)

^{1}H NMR (400 MHz, CDCl$_3$): Major diastereomer (*E*): δ (ppm): 15.06 (s, 1H), 8.69 (d, *J* = 4.8 Hz, 1H), 8.57 (m, 1H), 7.98 (d, *J* = 7.8 Hz, 1H), 7.82 (m, 1H), 7.76 (t, *J* = 7.8 Hz, 1H), 7.69 (d, *J* = 8.2 Hz, 1H), 7.37 (ddd, *J* = 7.5, 4.9, 1.2 Hz, 1H), 7.20 (dd, *J* = 7.7 Hz, 1H), 3.67 (t, *J* = 6.8 Hz, 2H), 3.23 (s, 3H), 3.04 (t, *J* = 6.8 Hz, 2H), 1.66 (m, 11 H). Minor diastereomer (*Z*): δ (ppm): 14.23 (s, 1H), 8.57 (d, *J* = 4.8 Hz, 1H), 7.98 (d, *J* = 7.8 Hz, 1H), 7.82 (m, 1H), 7.76 (t, *J* = 7.8 Hz, 1H), 7.47 (d, *J* = 8.0 Hz, 1H), 7.33 – 7.29 (m, 1H), 7.27 (d, *J* = 7.7 Hz, 1H), 3.87 (t, *J* = 7.0 Hz, 2H), 3.25 (s, 3H), 3.15 (t, *J* = 7.0 Hz, 2H), 1.66 (m, 11 H).

^{13}C NMR (101 MHz, CDCl$_3$): Mixture of *E*/*Z* isomers: δ (ppm): 181.4, 180.9, 156.5, 156.3, 156.2, 152.1, 151.6, 148.2, 147.0, 142.9, 141.8, 137.6, 137.6, 137.0, 136.9, 126.9, 125.0, 124.1, 123.8, 123.7, 123.3, 123.0, 122.8, 61.3, 60.1, 50.6, 50.5, 37.5, 37.0, 34.5, 33.7, 30.0, 29.8, 25.6, 25.5.

2-((4-(2-azidoethyl)pyridin-2-yl)(pyridin-2-yl)methylene)-*N,N*-dimethylhydrazine-1-carbothioamide (Dp44mT-P): Yield: 72% (0.17 g from 0.17 g of **P5**); **HRMS**: *m*/*z* found for $C_{16}H_{19}N_8S$ $[M+H]^+$: 355.1456 (calcd m/z: 355.1453)

^{1}H NMR (400 MHz, CDCl$_3$): Mixture of *E*/*Z* isomers: δ (ppm): 15.17 (s, 1H), 15.08 (s, 1H), 8.72 – 8.66 (m, 1H), 8.62 (d, *J* = 5.1 Hz, 1H), 8.58 (dt, *J* = 4.7, 1.6 Hz, 1H), 8.51 (d, *J* = 5.0 Hz, 1H), 8.14 (dd, *J* = 8.0, 1.2 Hz, 1H), 7.98 (d, *J* = 1.7 Hz, 1H), 7.83 (tt, *J* = 7.8, 2.0 Hz, 2H), 7.71 (dt, *J* = 8.2, 1.2 Hz, 1H), 7.59 (d, *J* = 1.7 Hz, 1H), 7.37 (ddd, *J* = 7.5, 5.0, 1.2 Hz, 1H), 7.34 – 7.29 (m, 1H), 7.25 (d, *J* = 1.6 Hz, 1H), 7.19 (dd, *J* = 5.0, 1.7 Hz, 1H), 3.64 (t, *J* = 7.0 Hz, 2H), 3.55 (t, *J* = 6.9 Hz, 2H), 2.99 (t, *J* = 7.0 Hz, 2H), 2.90 (t, *J* = 6.9 Hz, 2H).

^{13}C NMR (101 MHz, CDCl$_3$): Mixture of *E*/*Z* isomers: δ (ppm): 181.3, 156.7, 156.4, 152.2, 152.1, 148.7, 148.5, 148.4, 148.2, 147.3, 147.1, 143.1, 143.0, 137.4, 127.3, 127.1, 125.0, 124.9, 124.3, 124.0, 123.9, 123.5, 51.3, 51.0, 41.8, 34.9, 34.9.

2-((4-(2-azidoethyl)pyridin-2-yl)(pyridin-2-yl)methylene)-*N*-cyclohexyl-*N*-methylhydrazine-1-carbothioamide (DpC-P): Yield: 45% (0.13 g from 0.18 g of **P5**); **HRMS**: *m*/*z* found for $C_{21}H_{27}N_8S$ $[M+H]^+$: 423.2079 (calcd m/z: 423.2079)

^{1}H NMR (400 MHz, CDCl$_3$): Mixture of *E*/*Z* isomers: δ (ppm): 15.02 (s, 1H), 14.93 (s, 1H), 8.67 (dd, *J* = 5.0, 1.8 Hz, 1H), 8.60 (d, *J* = 5.1 Hz, 1H), 8.58 – 8.54 (m, 1H), 8.49 (d, *J* = 5.0 Hz, 1H), 8.15 (d, *J* = 7.9 Hz, 1H), 7.99 (s, 1H), 7.81 (ddt, *J* = 9.9, 5.0, 2.4 Hz, 2H), 7.69 (d, *J* = 8.2 Hz, 1H), 7.57 (s, 1H), 7.36 (dd, *J* = 7.6, 4.8 Hz, 1H), 7.30 (dd, *J* = 7.6, 4.8 Hz, 1H), 7.24 (dd, *J* = 5.1, 1.7 Hz, 1H), 7.18 (dd, *J* = 5.1, 1.8 Hz, 1H), 5.05 (s, 2H), 3.62 (t, *J* = 7.0 Hz, 2H), 3.53 (t, *J* = 6.9 Hz, 2H), 3.23 (d, *J* = 3.7 Hz, 5H), 2.97

(t, J = 7.0 Hz, 2H), 2.88 (t, J = 6.9 Hz, 2H), 2.19 – 1.62 (m, 11H), 1.46 (hept, J = 11.2, 9.8 Hz, 7H), 1.28 – 1.05 (m, 3H).

^{13}C NMR (101 MHz, $CDCl_3$): Mixture of *E*/*Z* isomers: δ (ppm): 180.9, 156.6, 152.1, 152.0, 148.6, 148.4, 148.2, 148.0, 147.2, 147.0, 137.3, 127.1, 126.9, 124.9, 124.8, 124.2, 123.8, 123.8, 123.3, 60.1, 51.2, 51.0, 34.8, 30.0, 25.7, 25.5.

ii) Synthesis of Linker

Synthesis of bis-BCN(PEG$_3$):

Synthesis of BCN-OSu (L1)

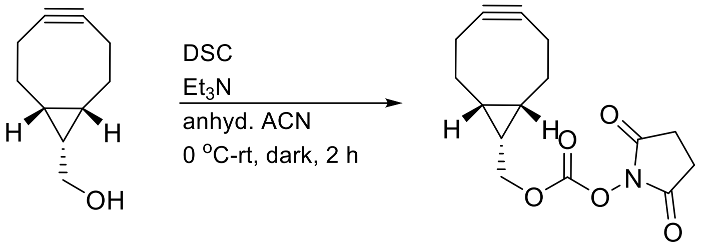

BCN-OH **BCN-OSu (L1)**

To a solution of BCN-OH (0.10 g, 0.67 mmol, 1.0 eq.) in anhydrous MeCN (4.0 mL) on ice disuccinimidyl carbonate (0.26 g, 1.01 mmol, 1.5 eq.) and distilled triethylamine (0.29 mL, 2.08 mmol, 3.0 eq.) were added. After the mixture was stirred for 1 h at room temperature under nitrogen and in the dark, it was concentrated *in vacuo* at 33 °C. The residue was purified by silica gel column chromatography (20 → 30% EtOAc/heptane) to afford the product as a white solid (0.16 g, 80% yield).

^{1}H NMR (400 MHz, $CDCl_3$): δ 4.38 (d, 2H, J = 8 Hz), 2.77 (s, 4H), 2.29-2.13 (6H, m), 1.54-1.39 (3H, m), 1.04-0.94 (2H, m).

^{13}C NMR (101 MHz, $CDCl_3$): δ 168.7, 151.6, 98.7, 70.3, 29.0, 25.5, 21.5, 20.7, 17.2.

Synthesis of Bis-BCN(PEG$_3$) (L2)

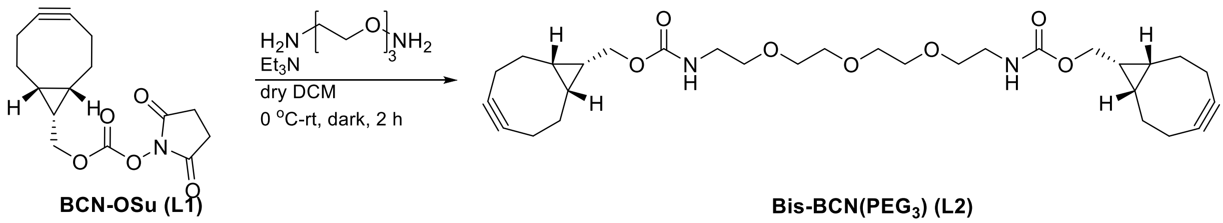

BCN-OSu (L1) **Bis-BCN(PEG$_3$) (L2)**

To a solution of 1,11-Diamino-3,6,9-trioxaundecane (15.00 mg, 0.078 mmol, 1.0 eq.) in 2.5 mL dry DCM at 0 °C, triethylamine (25.00 µL, 0.18 mmol, 2.3 eq.) followed by BCN-OSu (49.60 mg, 0.17 mmol, 2.1 eq.) were added. After stirring at room temperature for 1 h under an argon atmosphere in the dark, the reaction was quenched with 10 mL saturated NH_4Cl and extracted with DCM (3x10 mL). The combined organic layers were washed with saturated $NaHCO_3$ (10 mL), dried over Na_2SO_4, and evaporated *in vacuo* at 33 °C. The residue was then purified via

silica gel chromatography (50/50 v/v% EtOAc/heptane) to afford the product as a colorless oil (77.00 mg, 91% yield).

^{1}H NMR (400 MHz, $CDCl_3$): δ (ppm) = 5.23 (br s, 2H), 4.16 (d, *J* = 8 Hz, 4H), 3.63 (br s, 8H), 3.58-3.55 (t, *J* = 8 Hz, 4H), 3.39-3.35 (m, 4H), 2.33-2.18 (m, 12H), 1.66-1.53 (m, 5H), 1.40-1.31 (m, 2H), 0.98-0.89 (m, 4H).

^{13}C NMR (101 MHz, $CDCl_3$): δ (ppm) = 156.8, 98.8, 70.5, 70.3, 70.1, 62.7, 40.8, 29.1, 21.4, 20.1, 17.8.

HR-MS: *m*/*z* found for $C_{30}H_{45}N_2O_7$ $[M+H]^+$: 545.3219 (calcd m/z: 545.3227)

4. Spectroscopic information

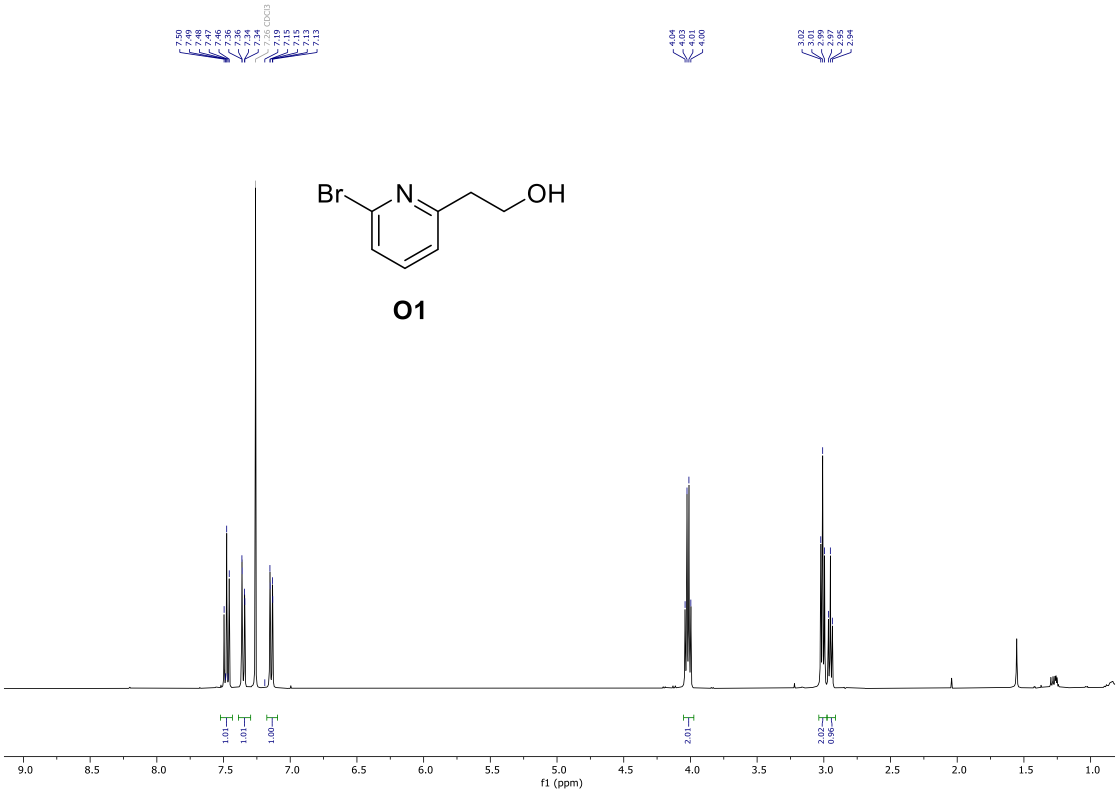

Figure S1: ^{1}H NMR Spectrum of **O1**.

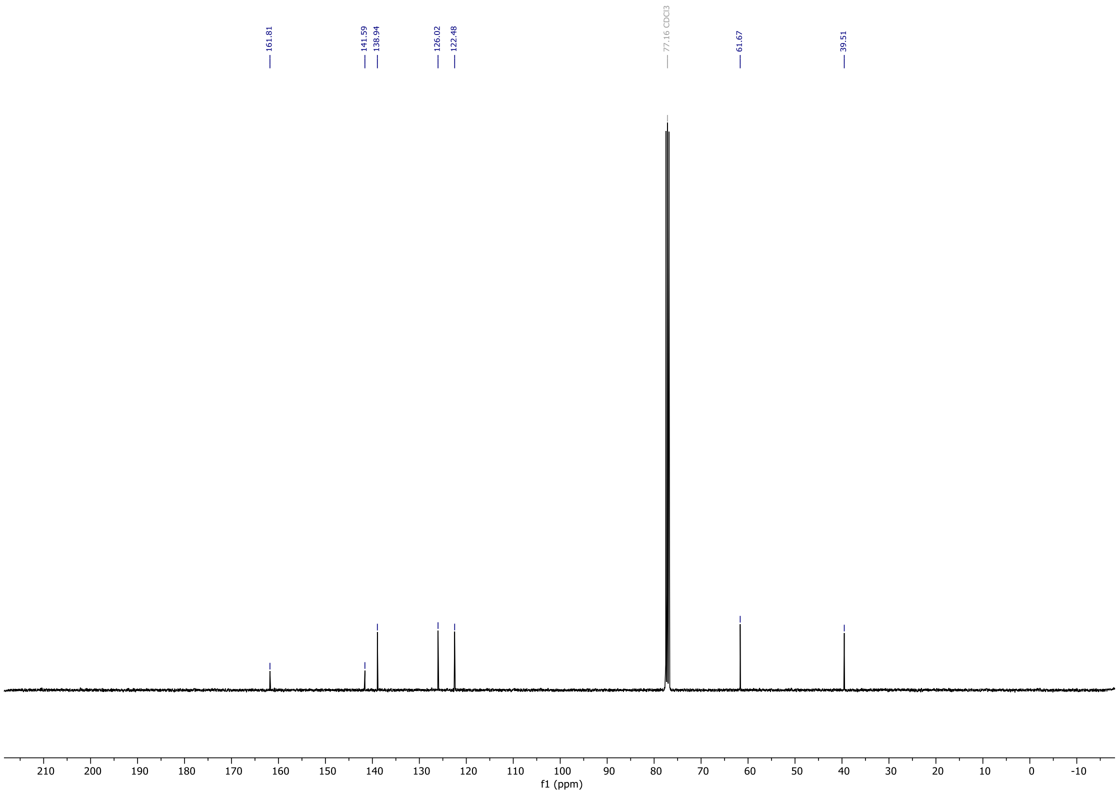

Figure S2: ^{13}C NMR Spectrum of **O1**.

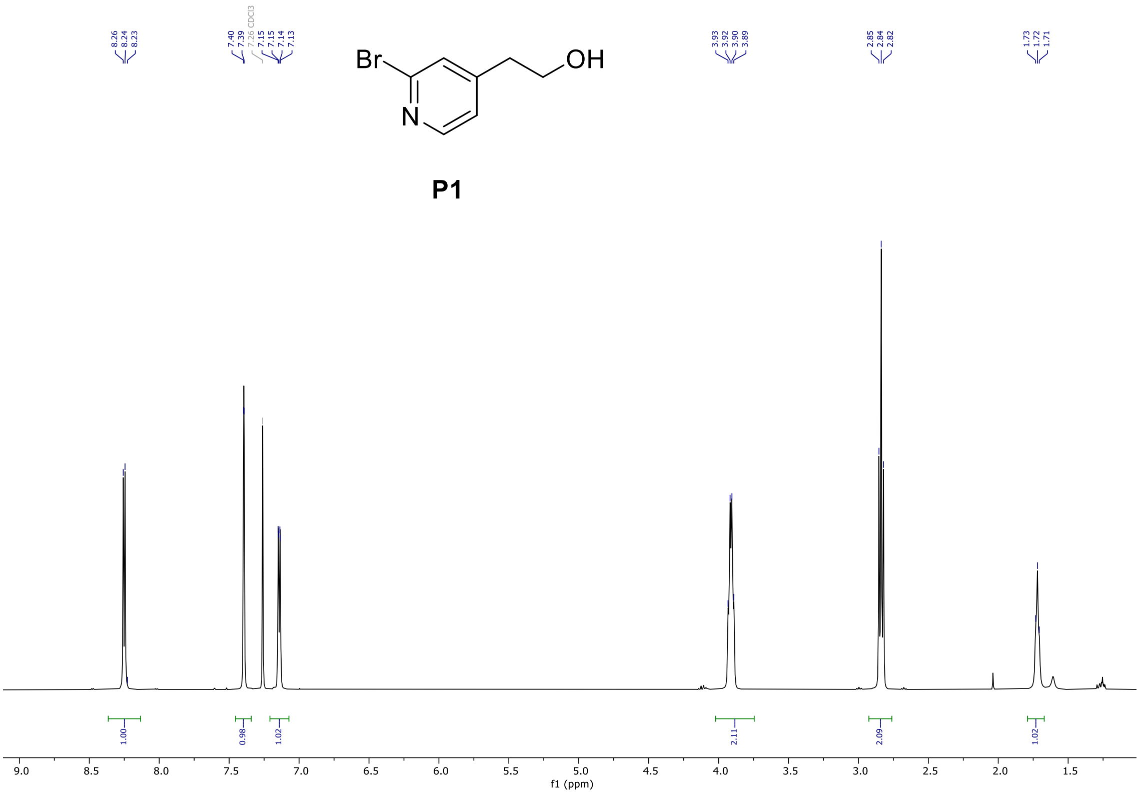

Figure S3: ^{1}H NMR Spectrum of **P1**.

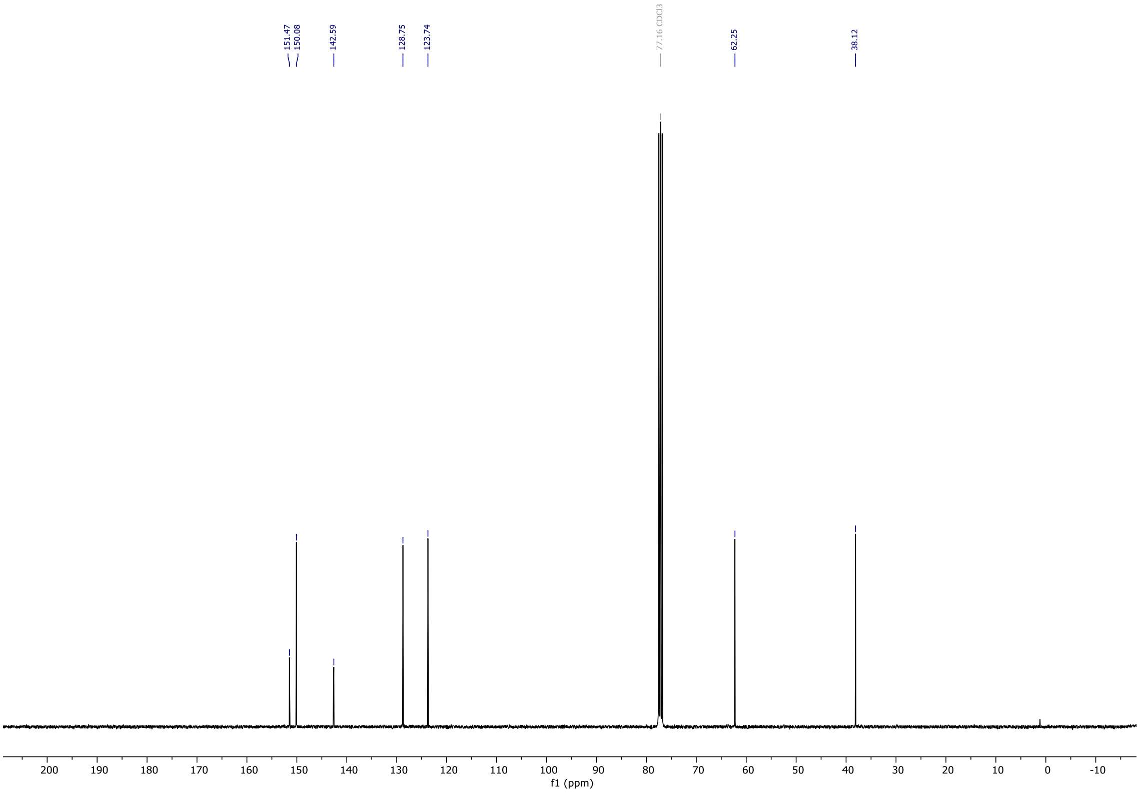

Figure S4: ^{13}C NMR Spectrum of **P1**.

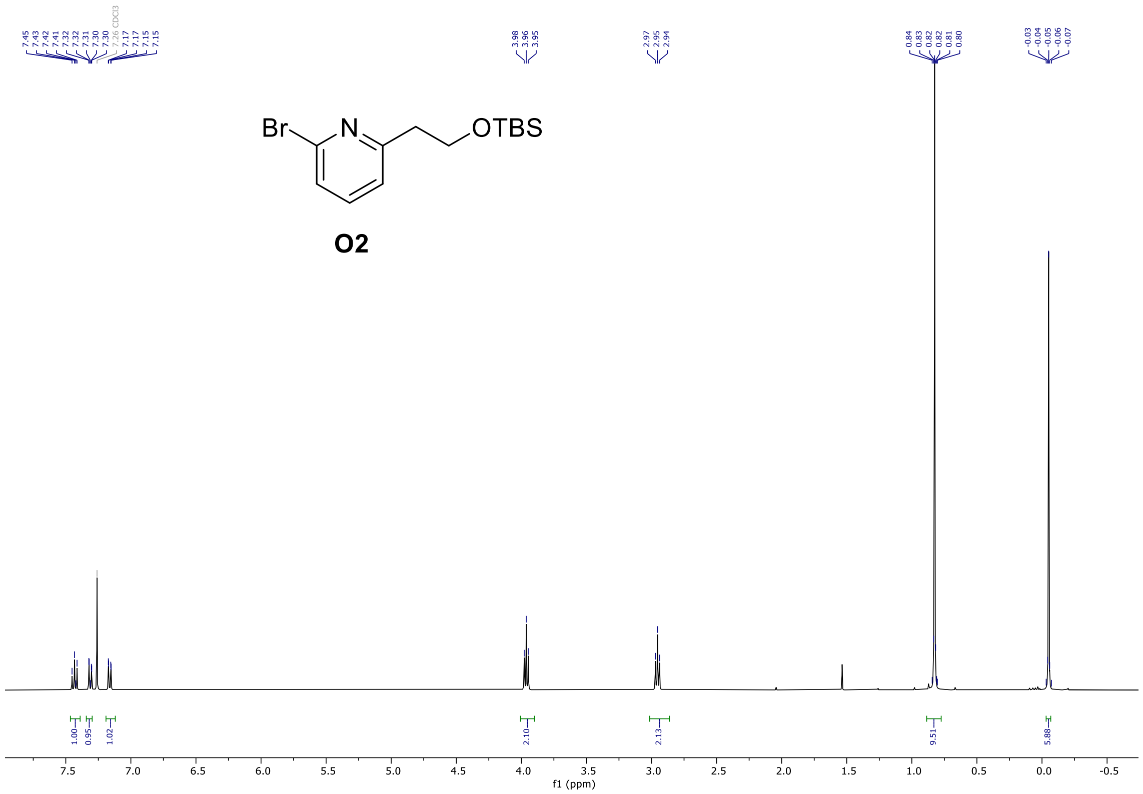

Figure S5: ^{1}H NMR Spectrum of **O2.**

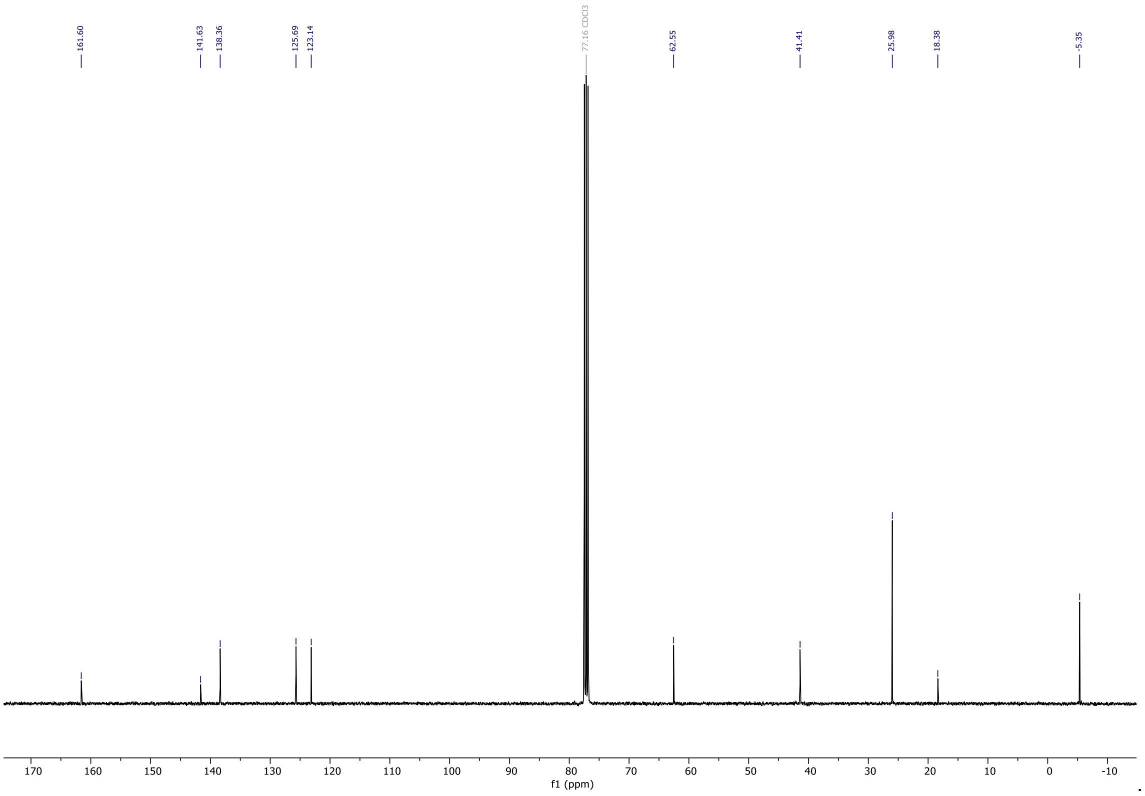

Figure S6: ^{13}C NMR Spectrum of **O2.**

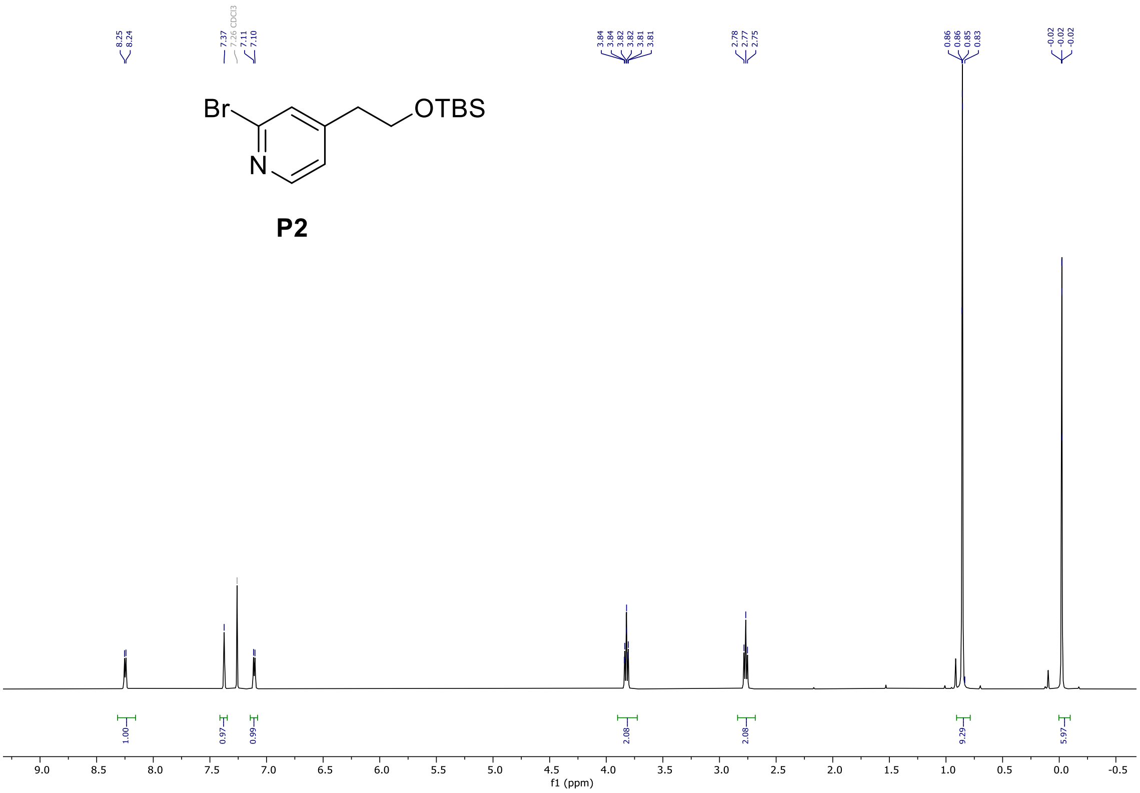

Figure S7: ^{1}H NMR Spectrum of **P2.**

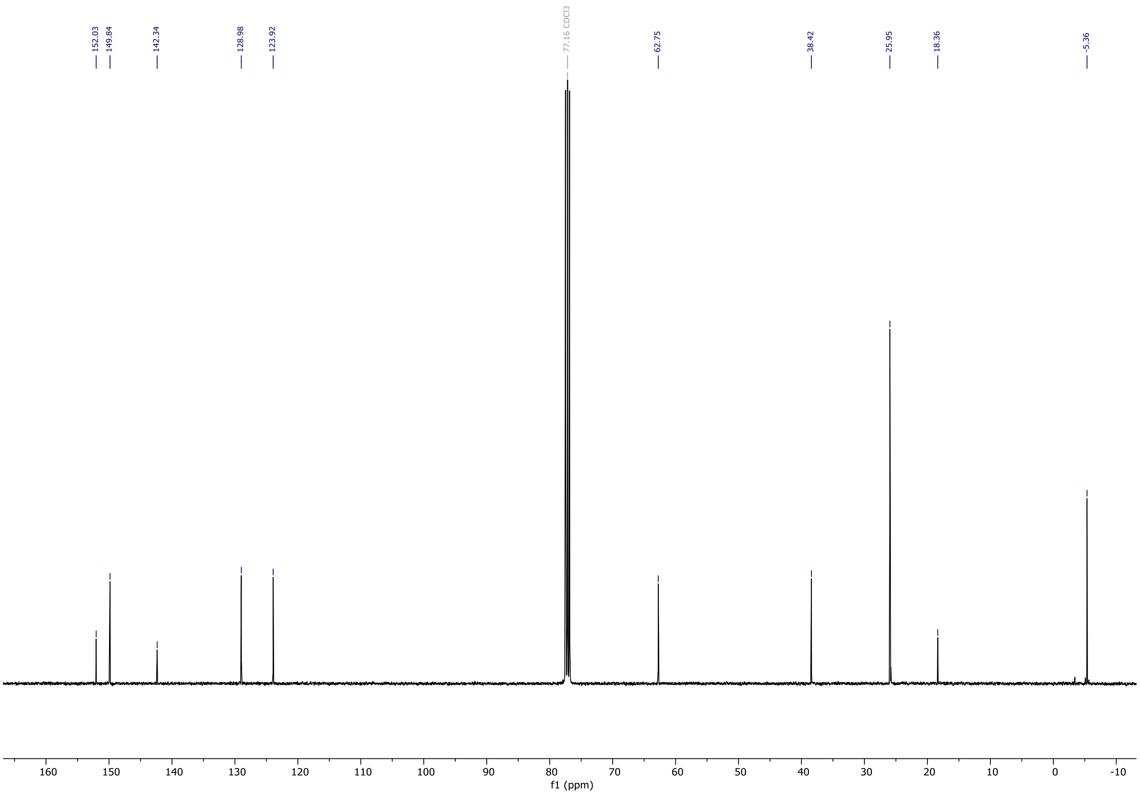

Figure S8: ^{13}C NMR Spectrum of **P2.**

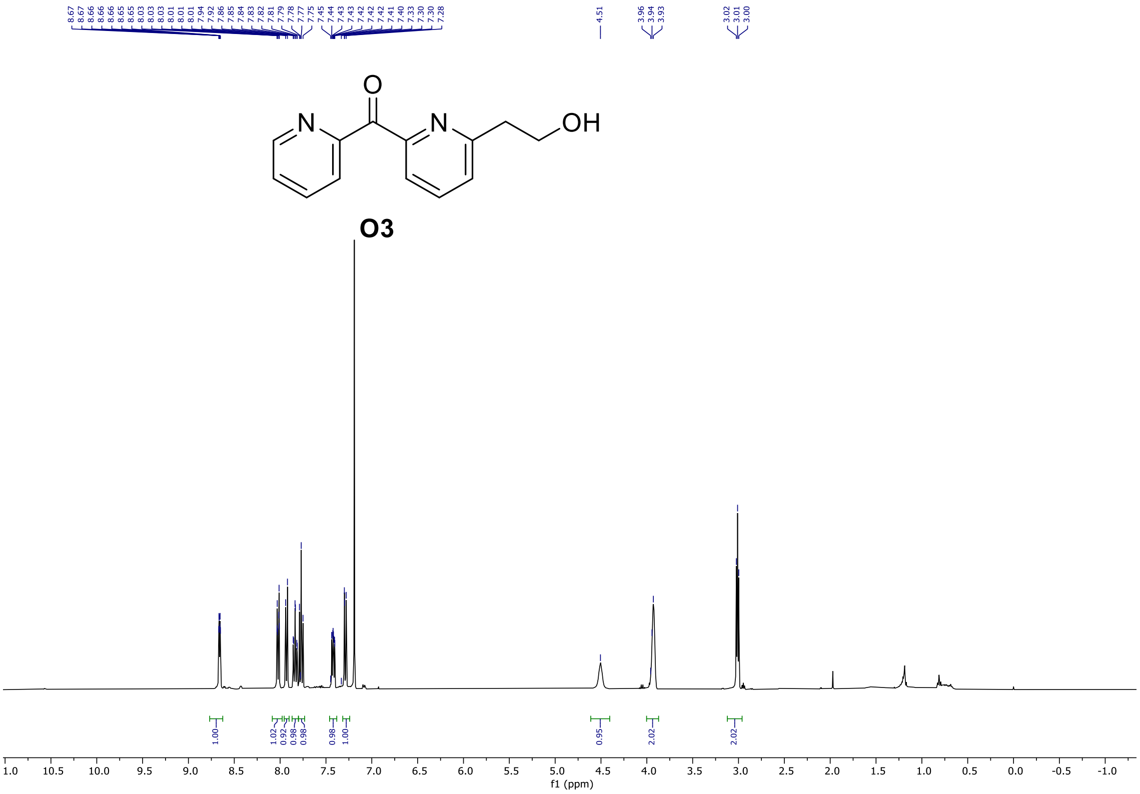

Figure S9: ^{1}H NMR Spectrum of **O3.**

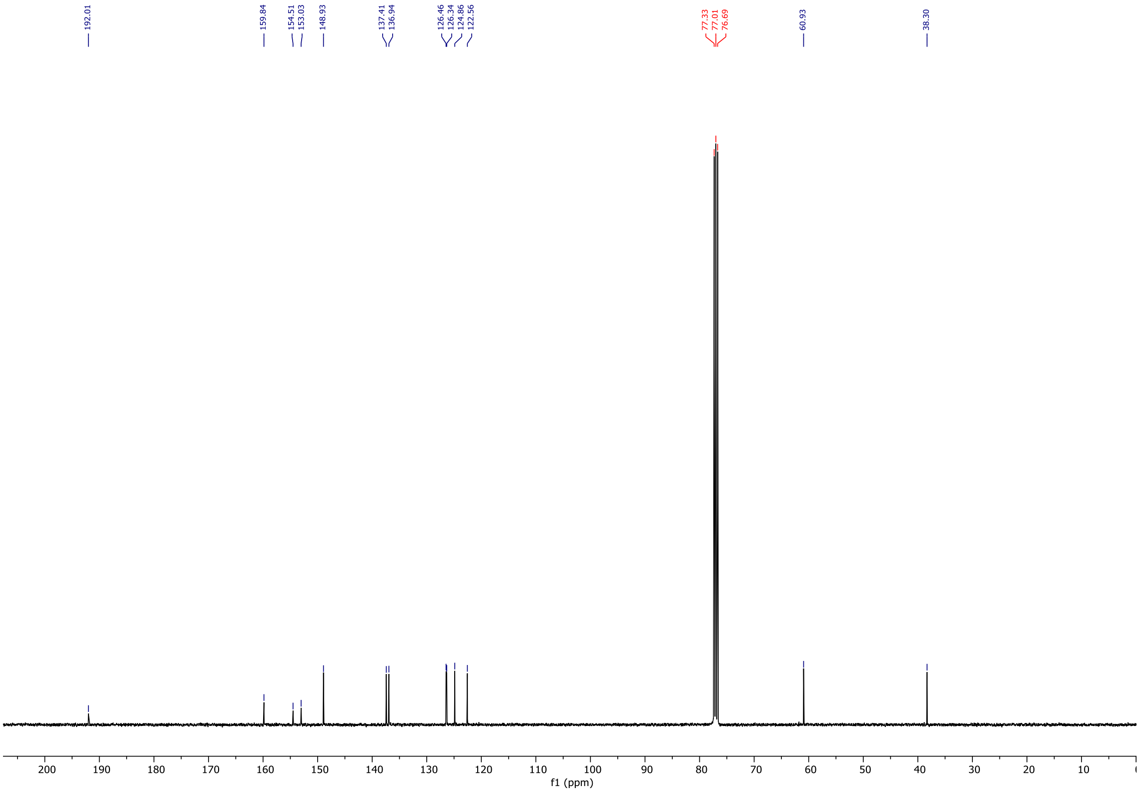

Figure S10: ^{13}C NMR Spectrum of **O3.**

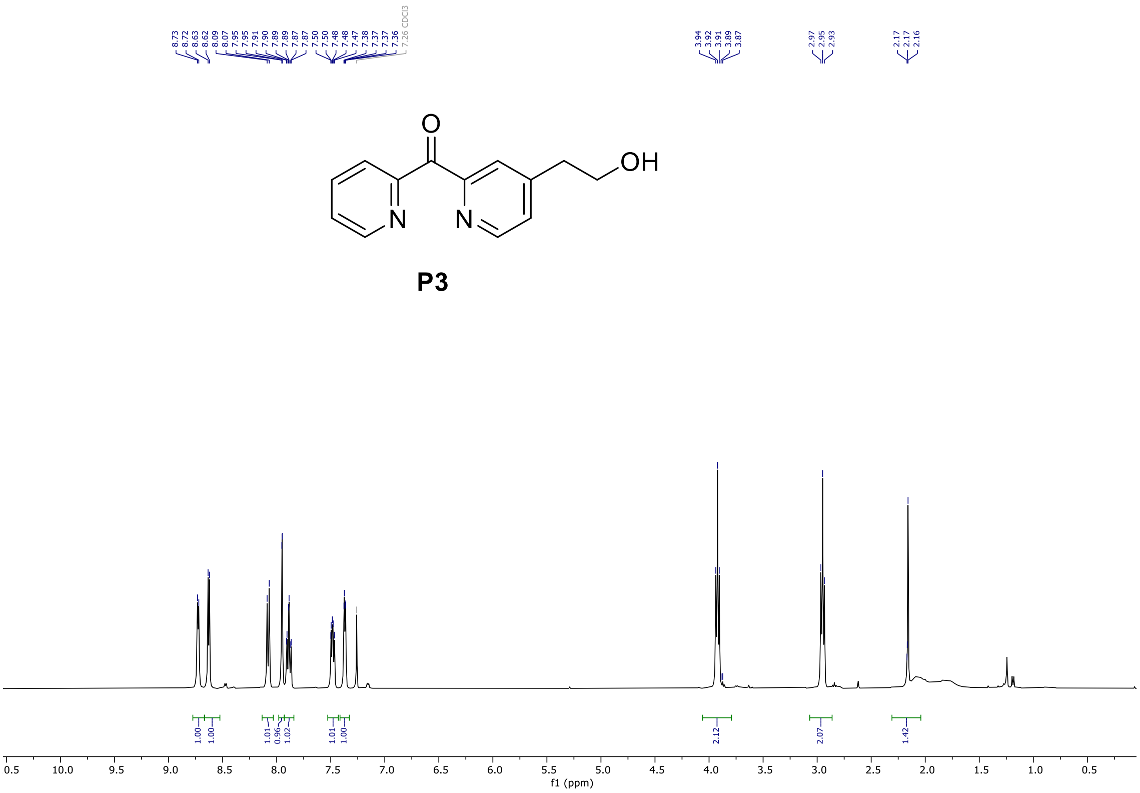

Figure S11: ^{1}H NMR Spectrum of **P3.**

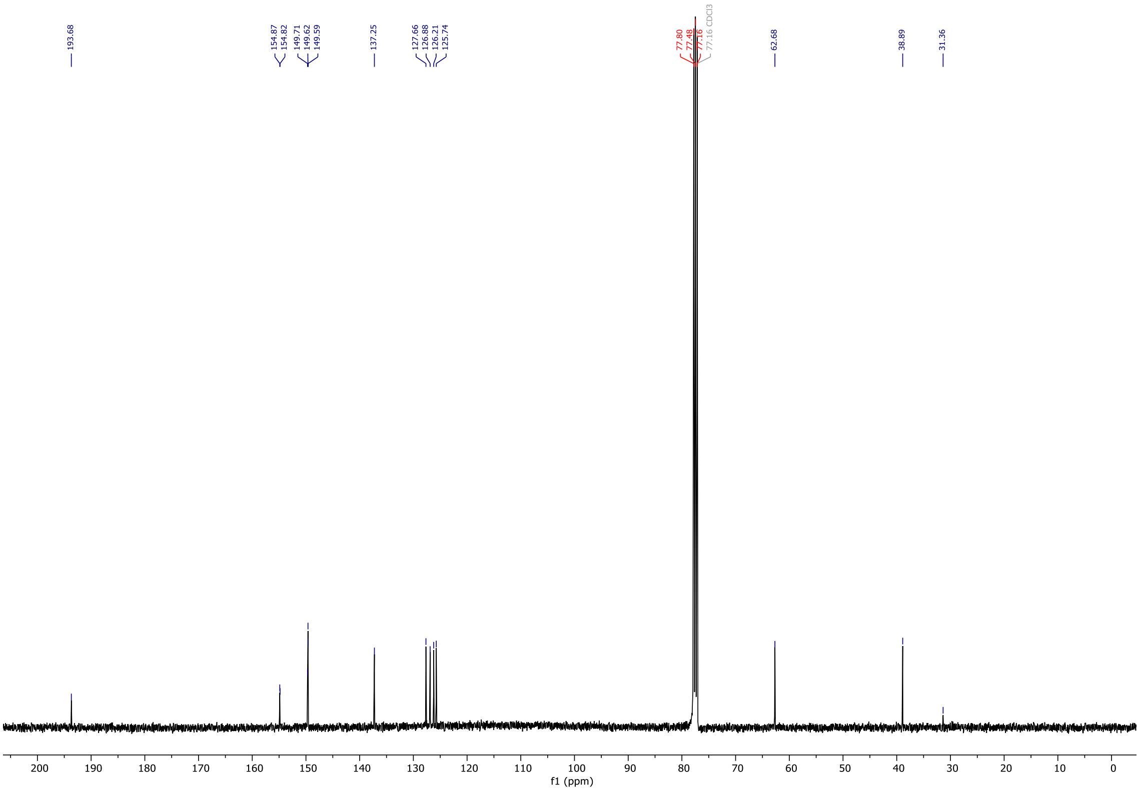

Figure S12: ^{13}C NMR Spectrum of **P3.**

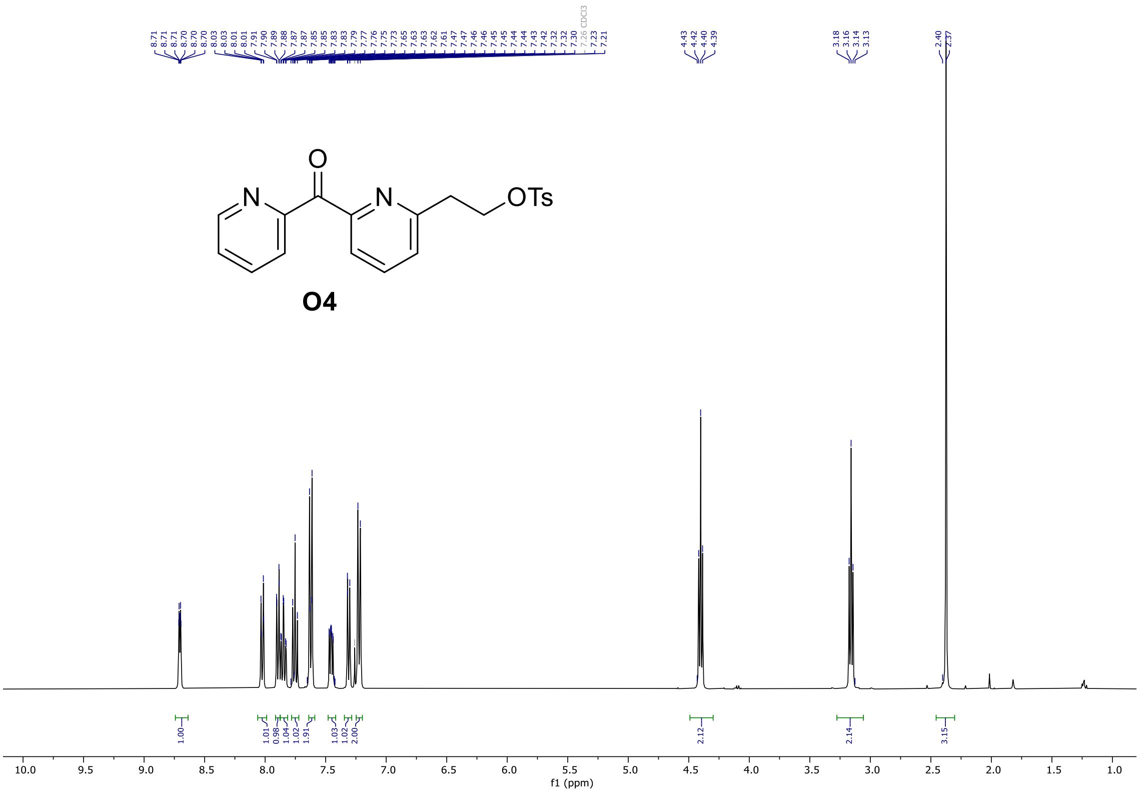

Figure S13: ^{1}H NMR Spectrum of **O4.**

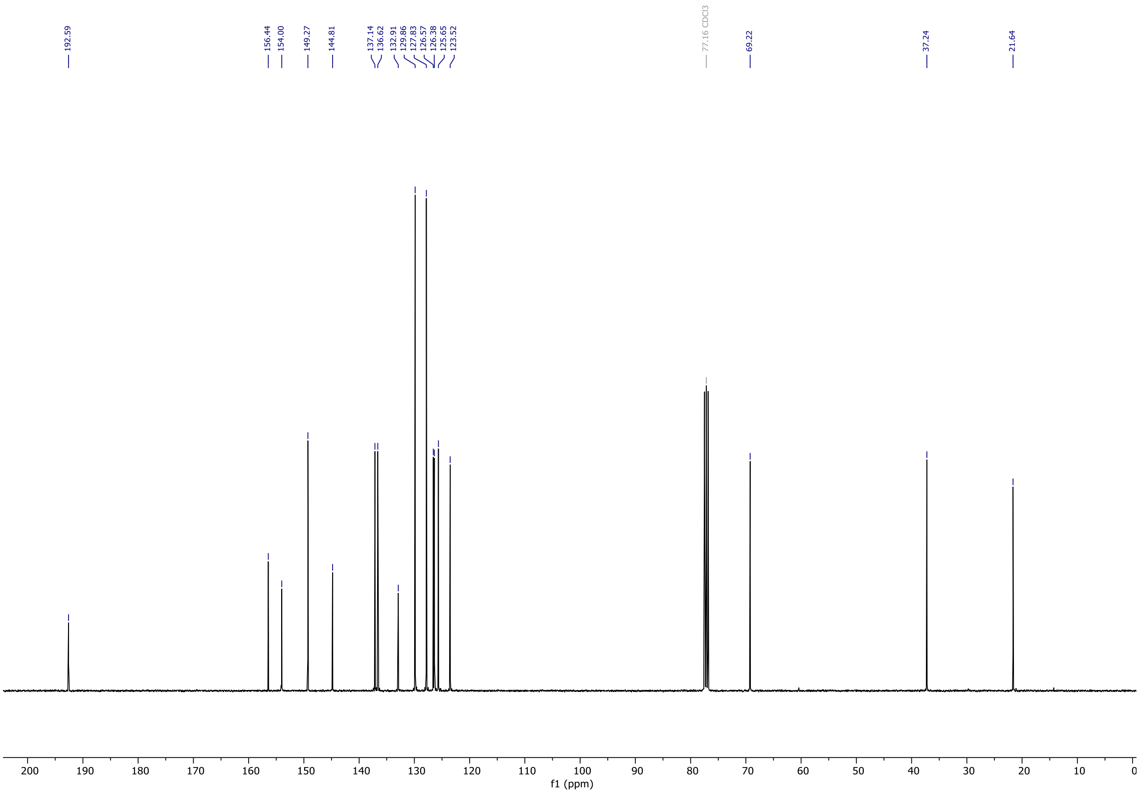

Figure S14: ^{13}C NMR Spectrum of **O4.**

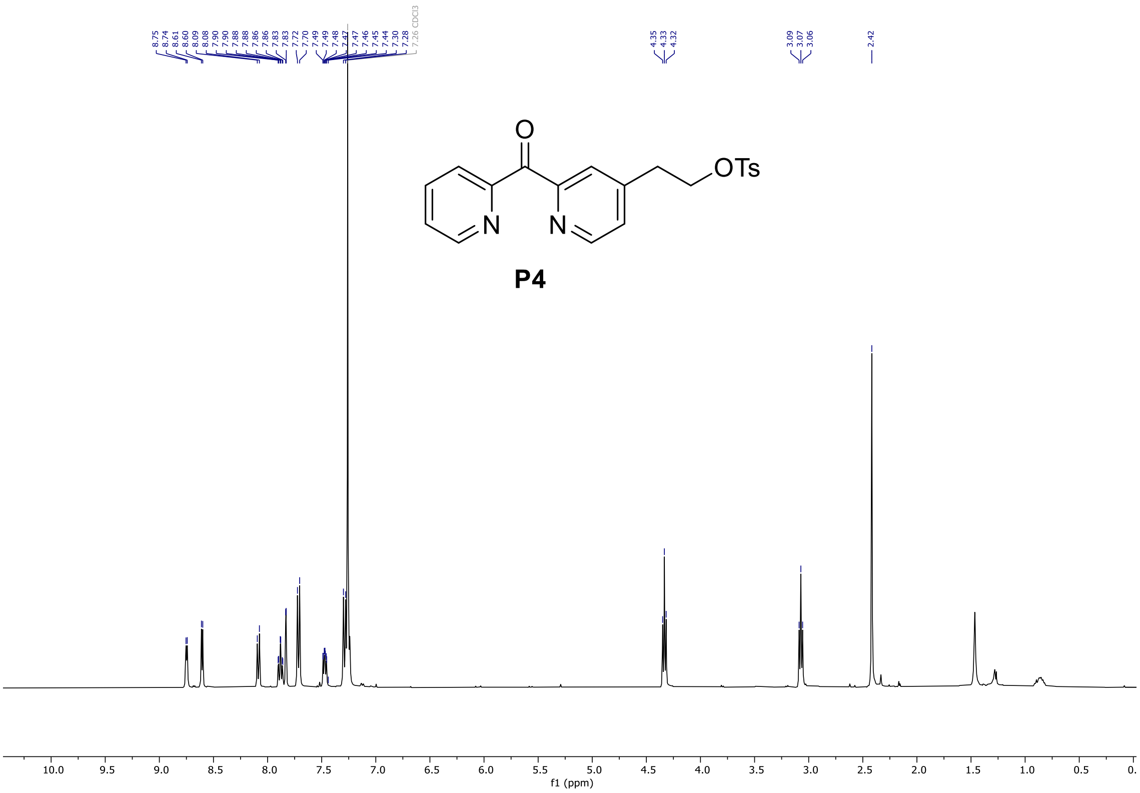

Figure S15: ^{1}H NMR Spectrum of **P4.**

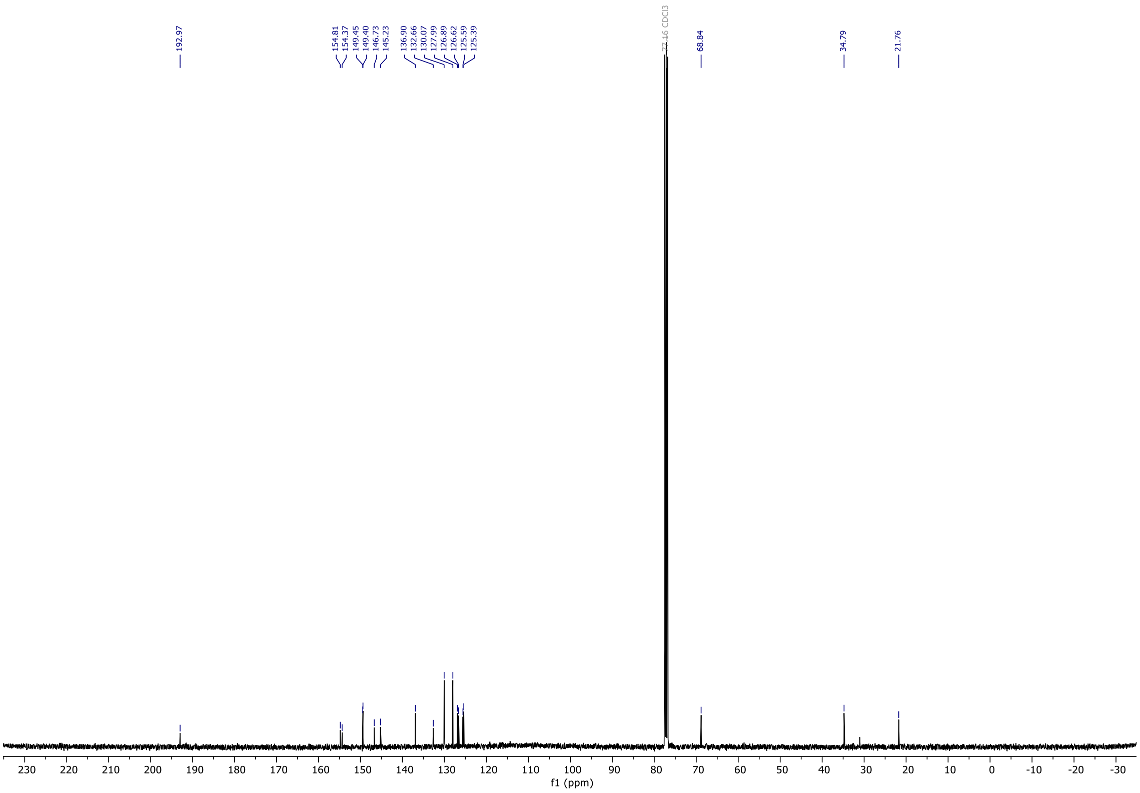

Figure S16: ^{13}C NMR Spectrum of **P4.**

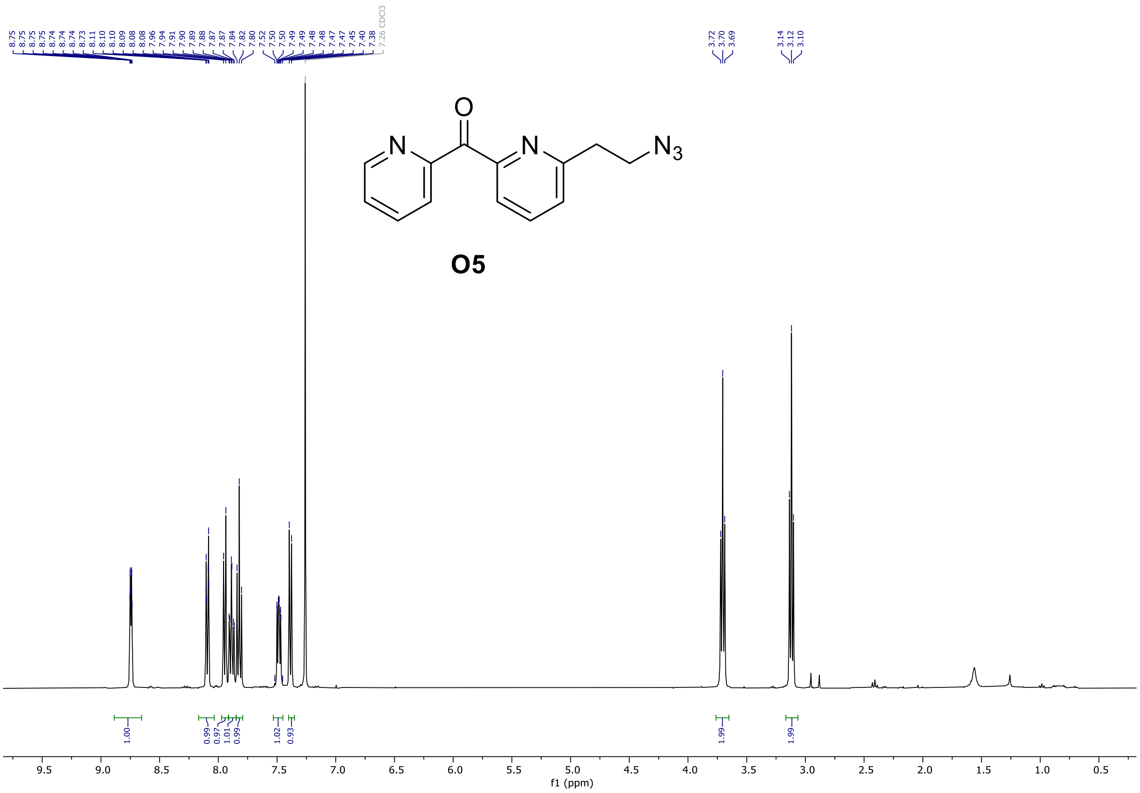

Figure S17: ^{1}H NMR Spectrum of **O5.**

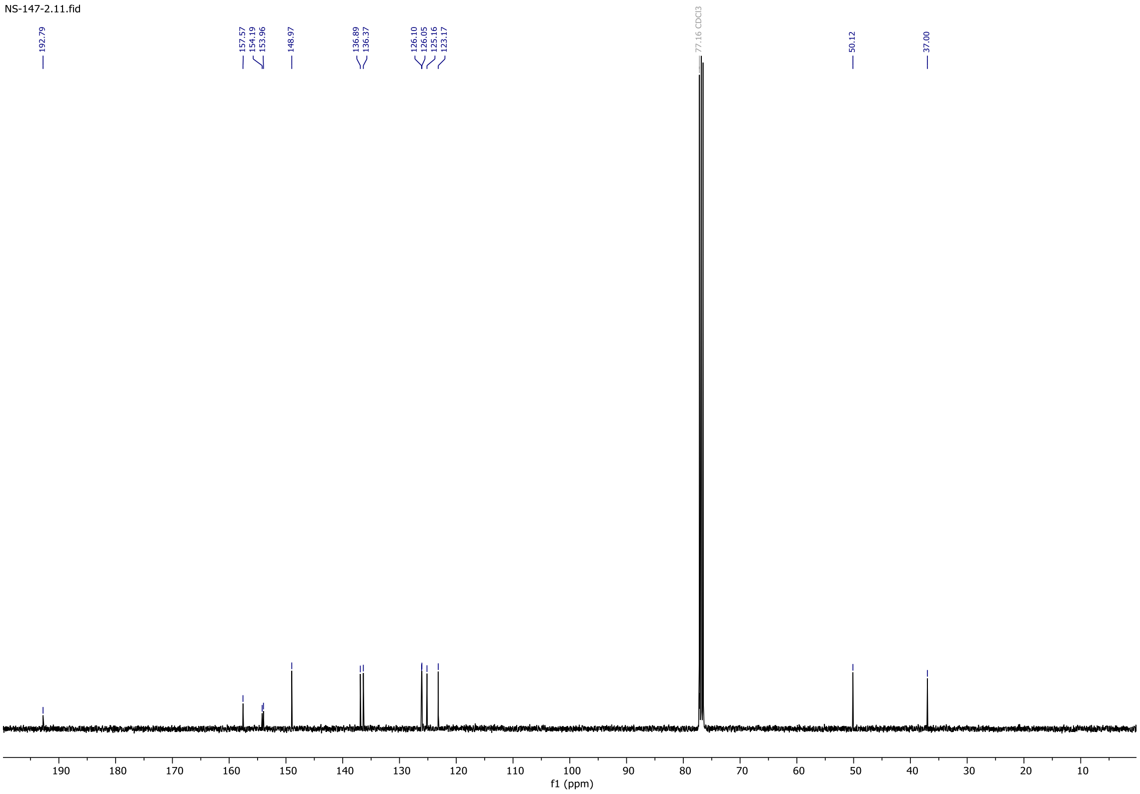

Figure S16: ^{13}C NMR Spectrum of **O5.**

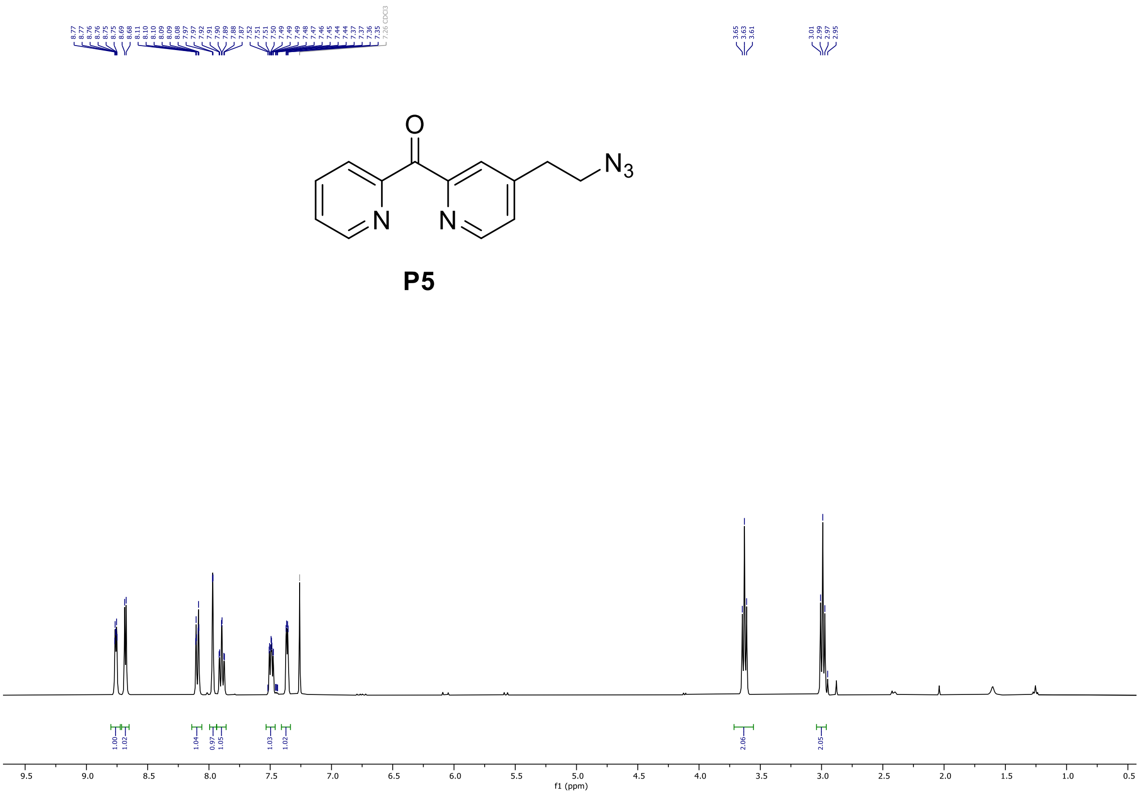

Figure S17: ^{1}H NMR Spectrum of **P5.**

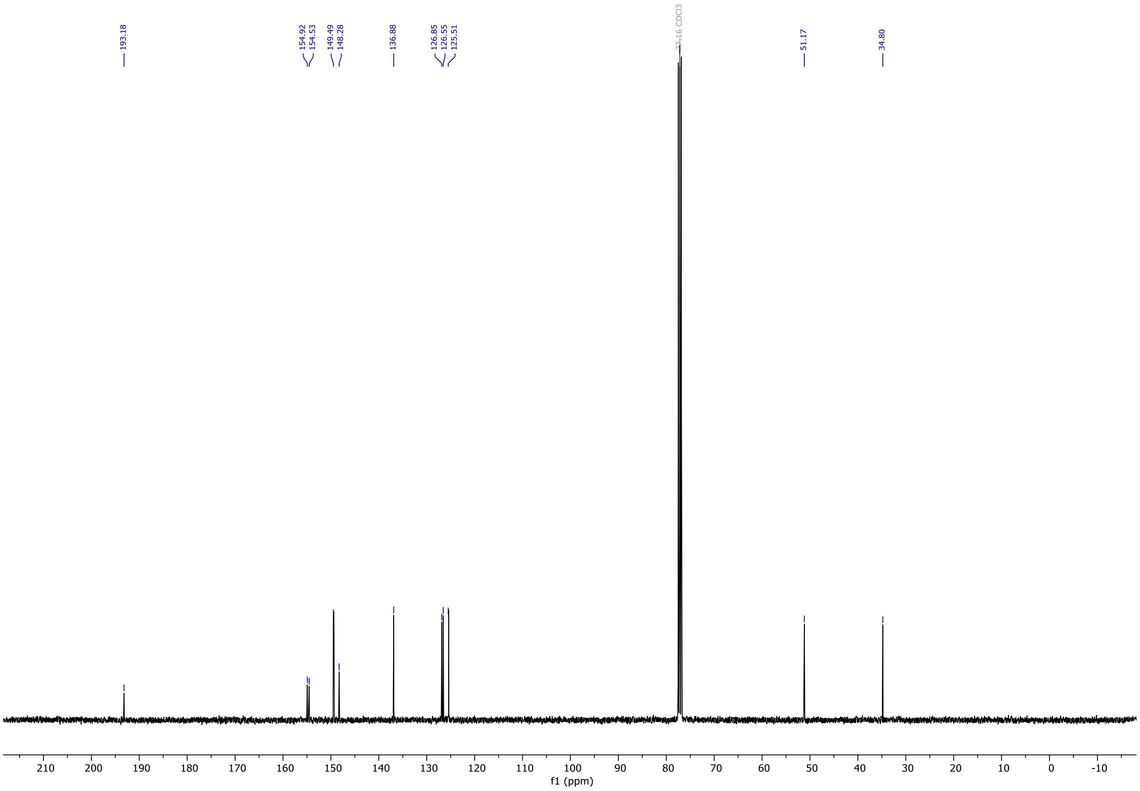

Figure S18: ^{13}C NMR Spectrum of **P5.**

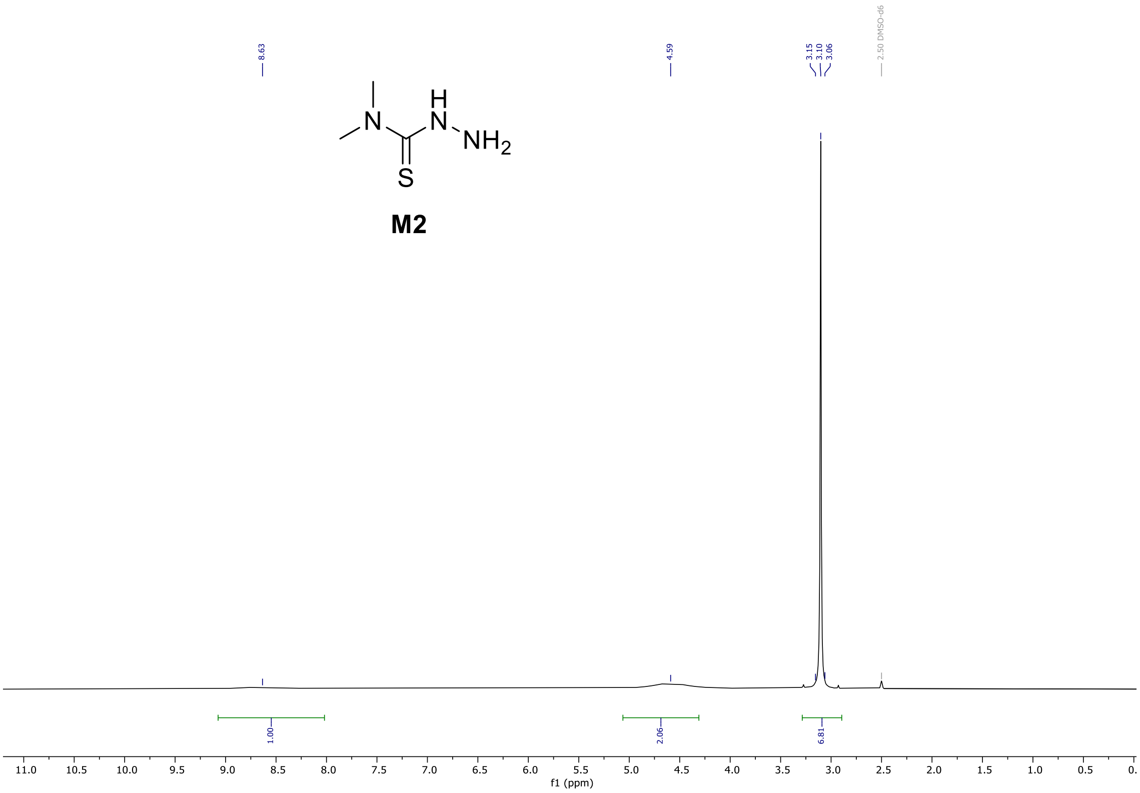

Figure S19: ^{1}H NMR Spectrum of **M2**

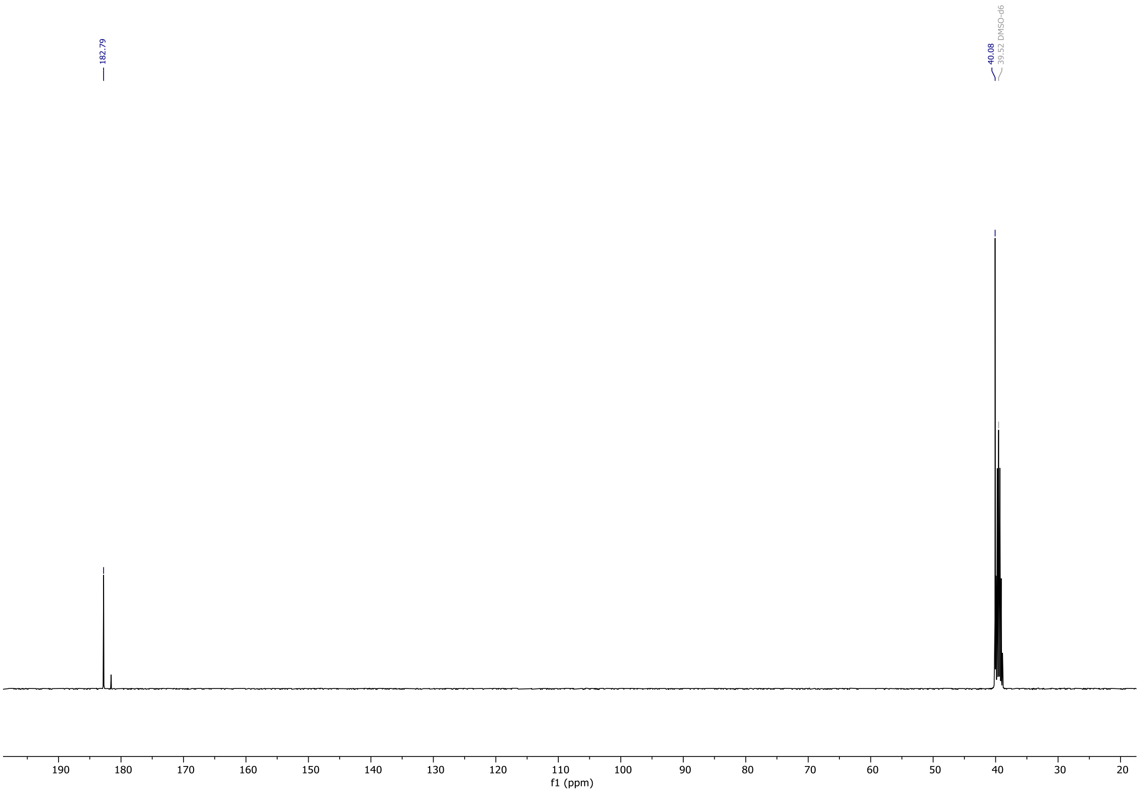

Figure S20: ^{13}C NMR Spectrum of **M2.**

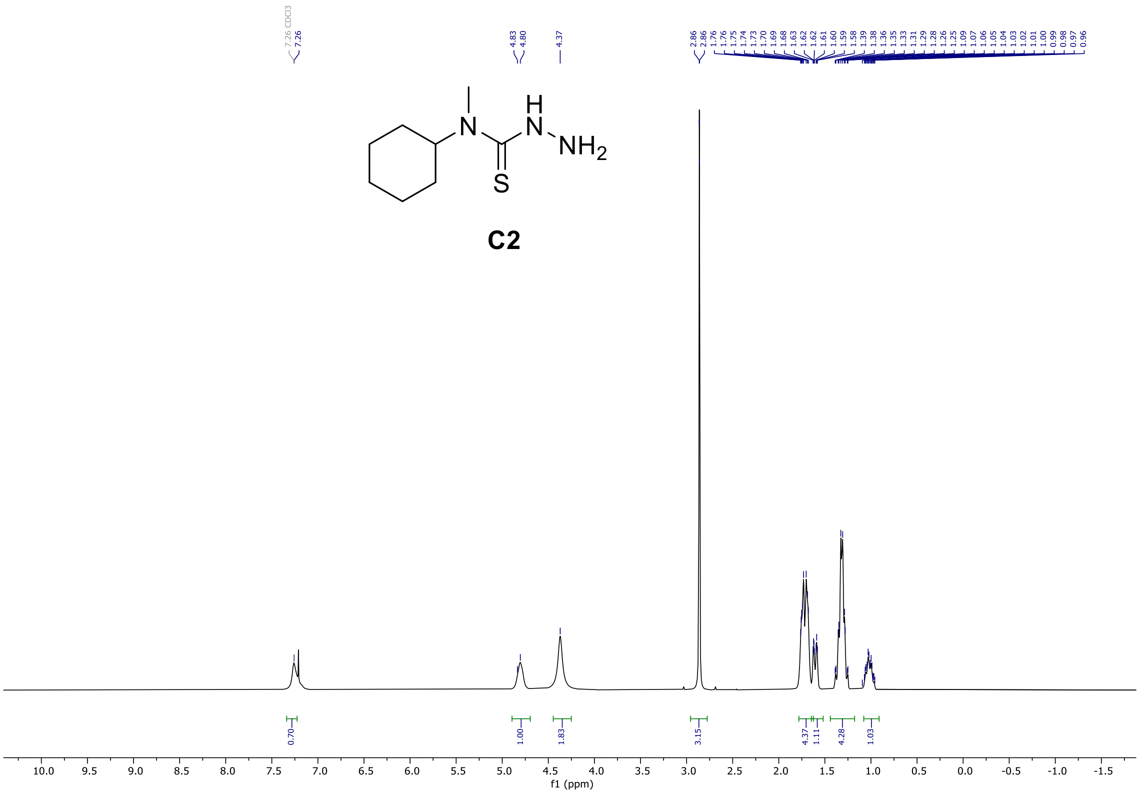

Figure S21: ^{1}H NMR Spectrum of **C2**

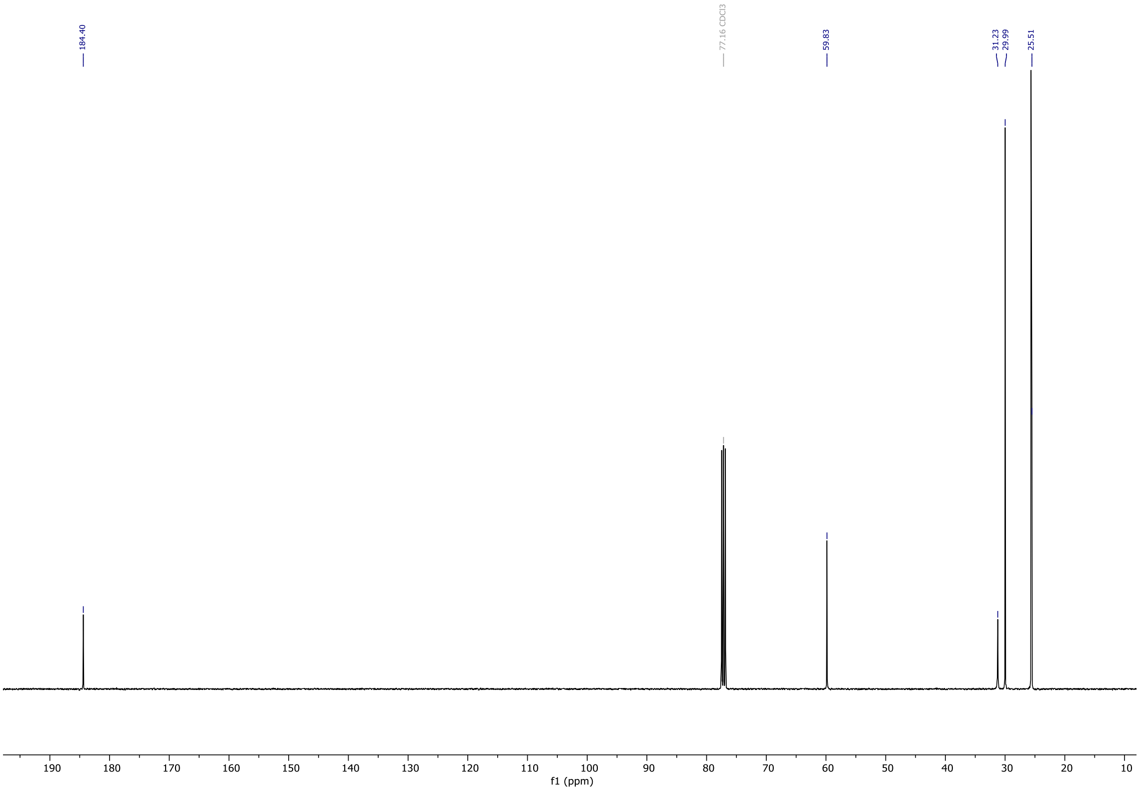

Figure S22: ^{13}C NMR Spectrum of **C2.**

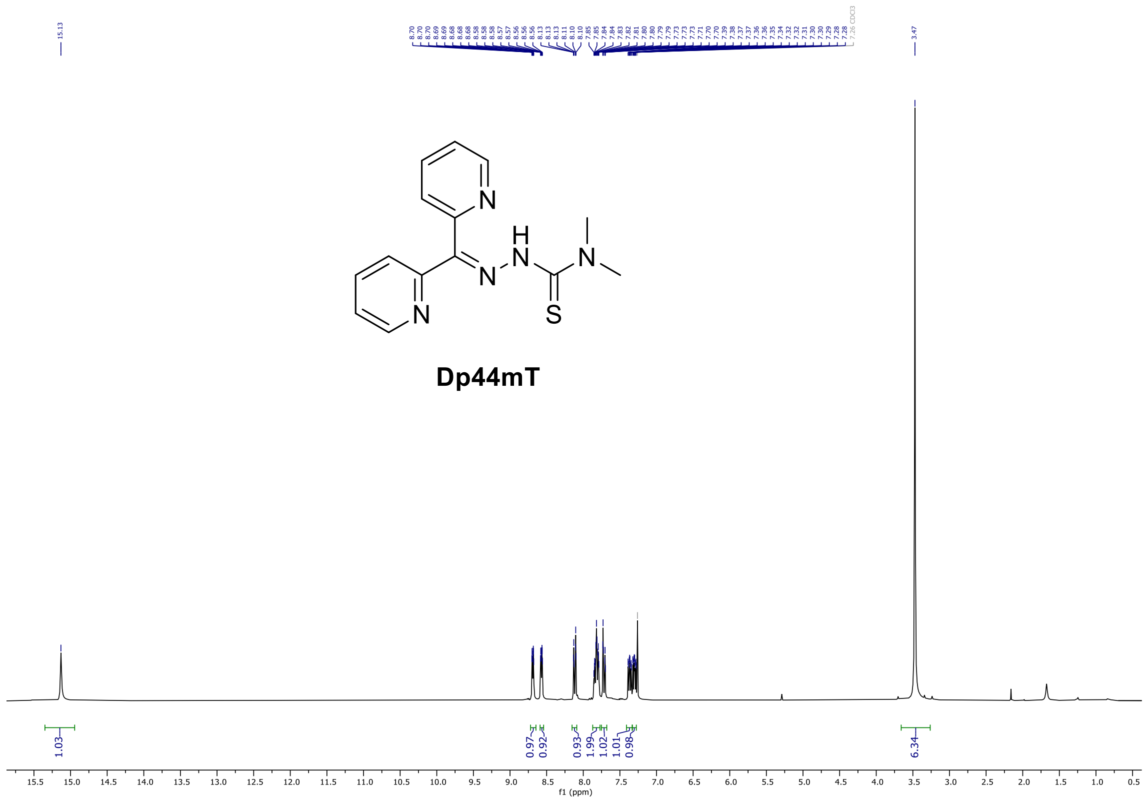

Figure S23: ^{1}H NMR Spectrum of **Dp44mT.**

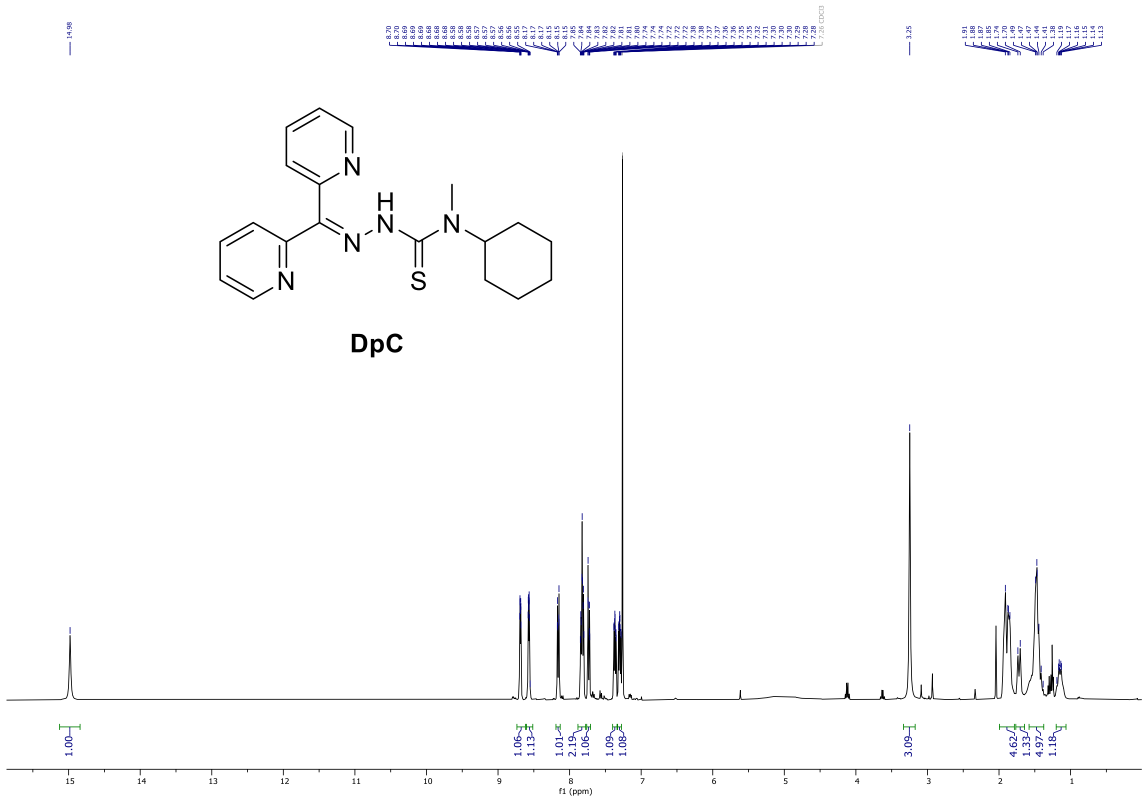

Figure S24: ^{1}H NMR Spectrum of **DpC.**

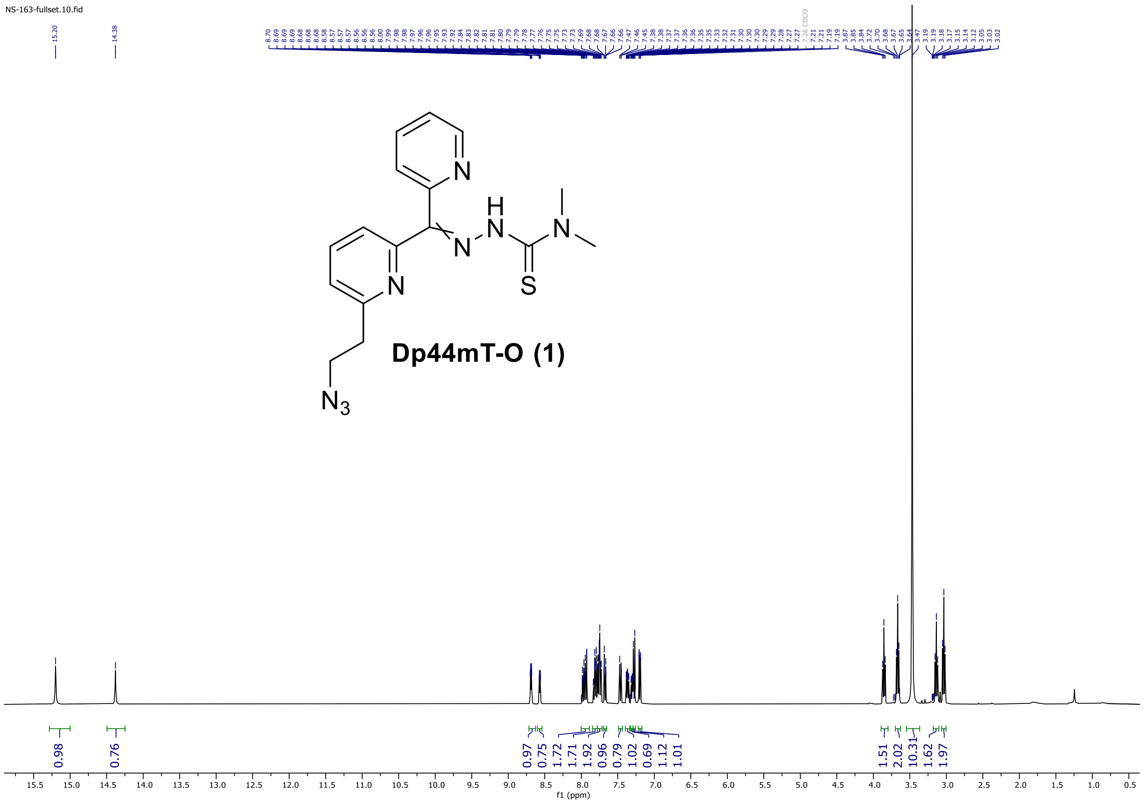

Figure S25: ^{1}H NMR Spectrum of **1.**

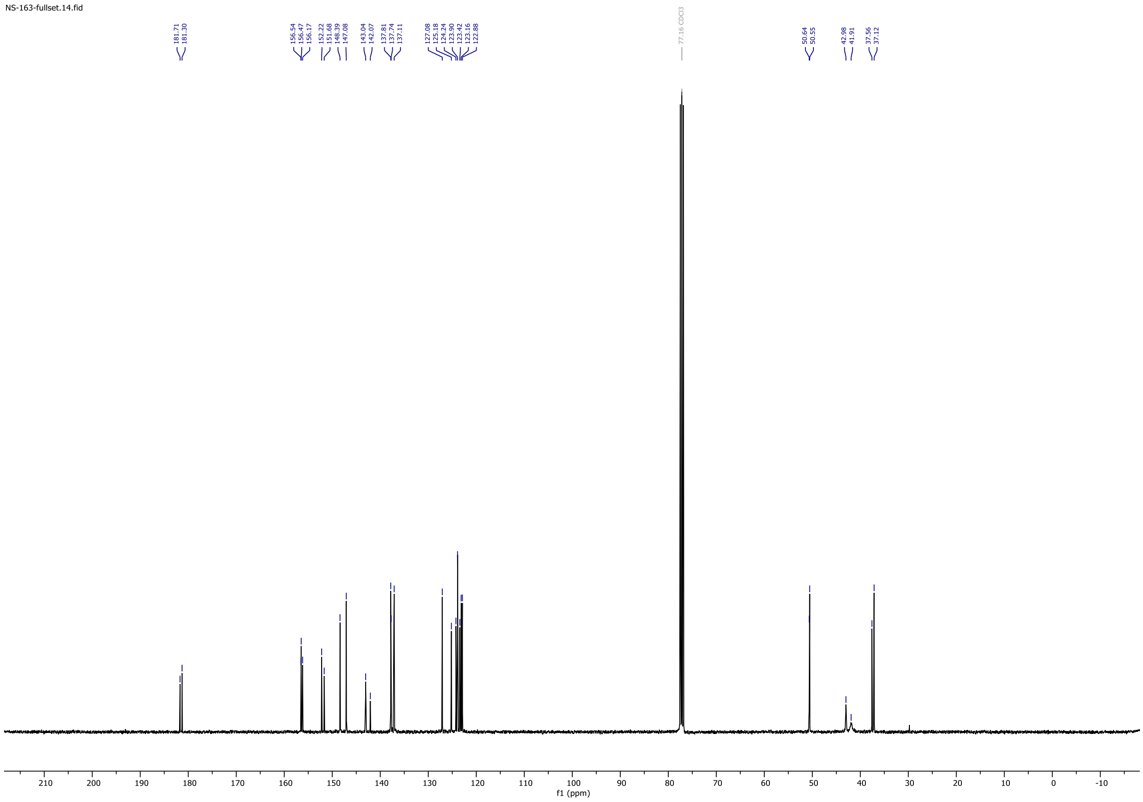

Figure S26: ^{13}C NMR Spectrum of **1.**

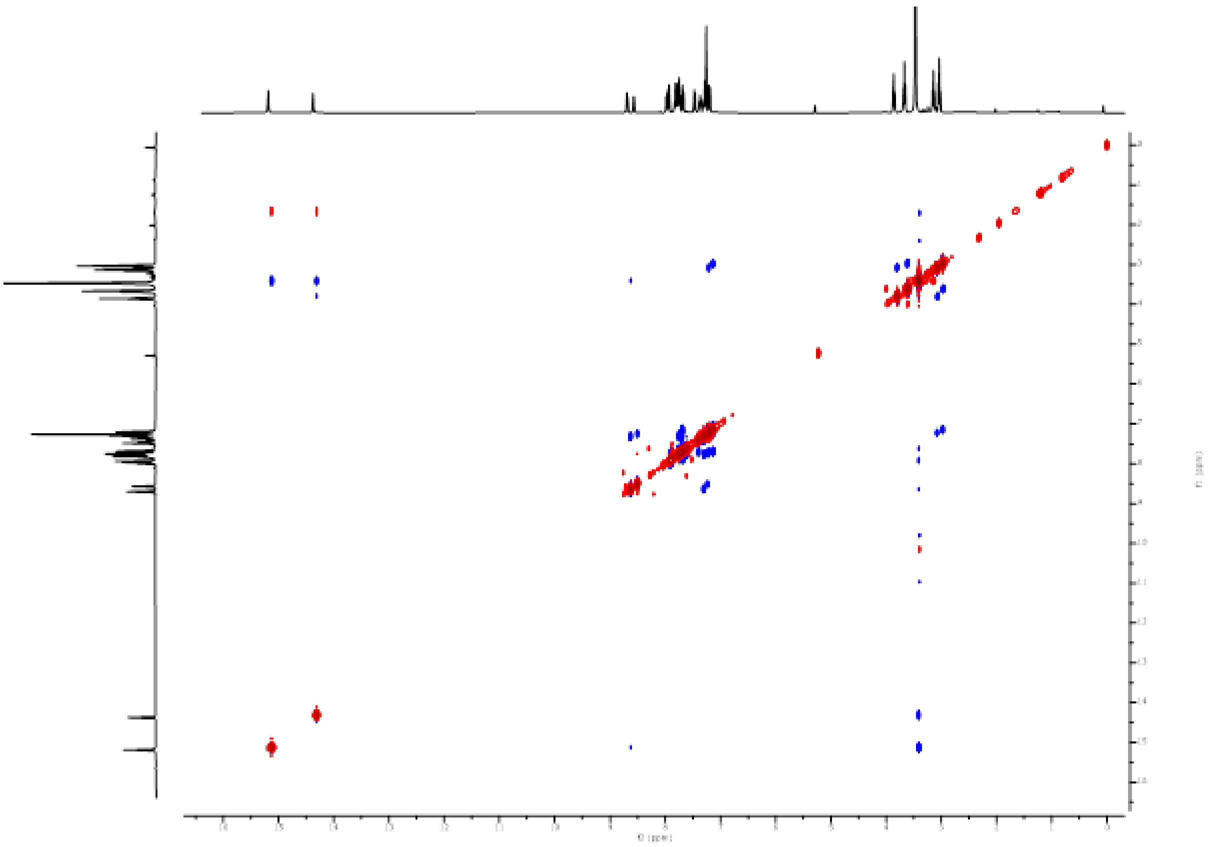

Figure S27: HH NOESY Spectrum of **1.**

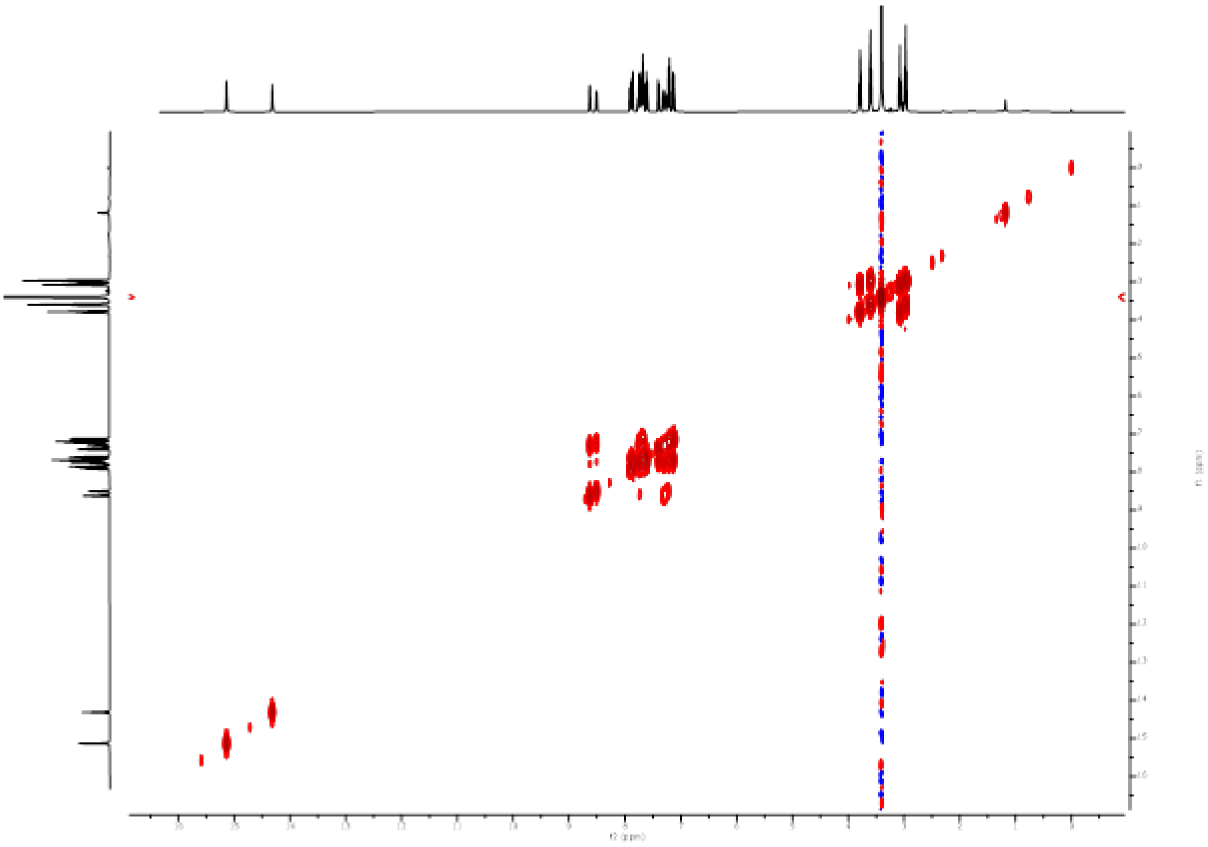

Figure S28: COSY Spectrum of **1.**

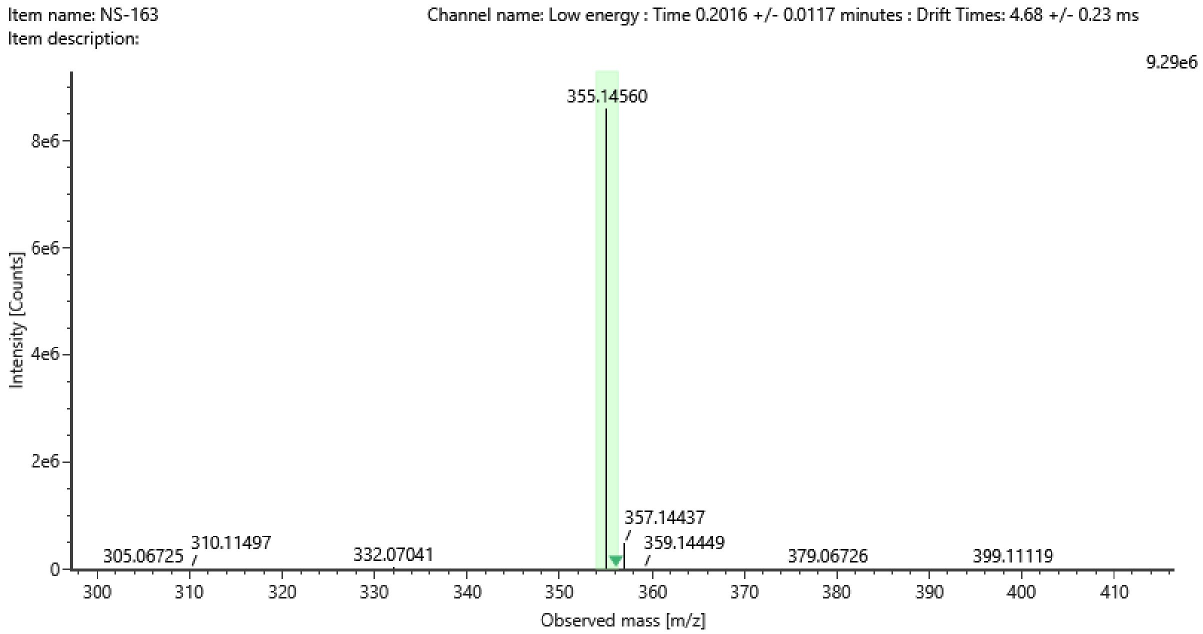

Figure S29: HRMS of **1.**

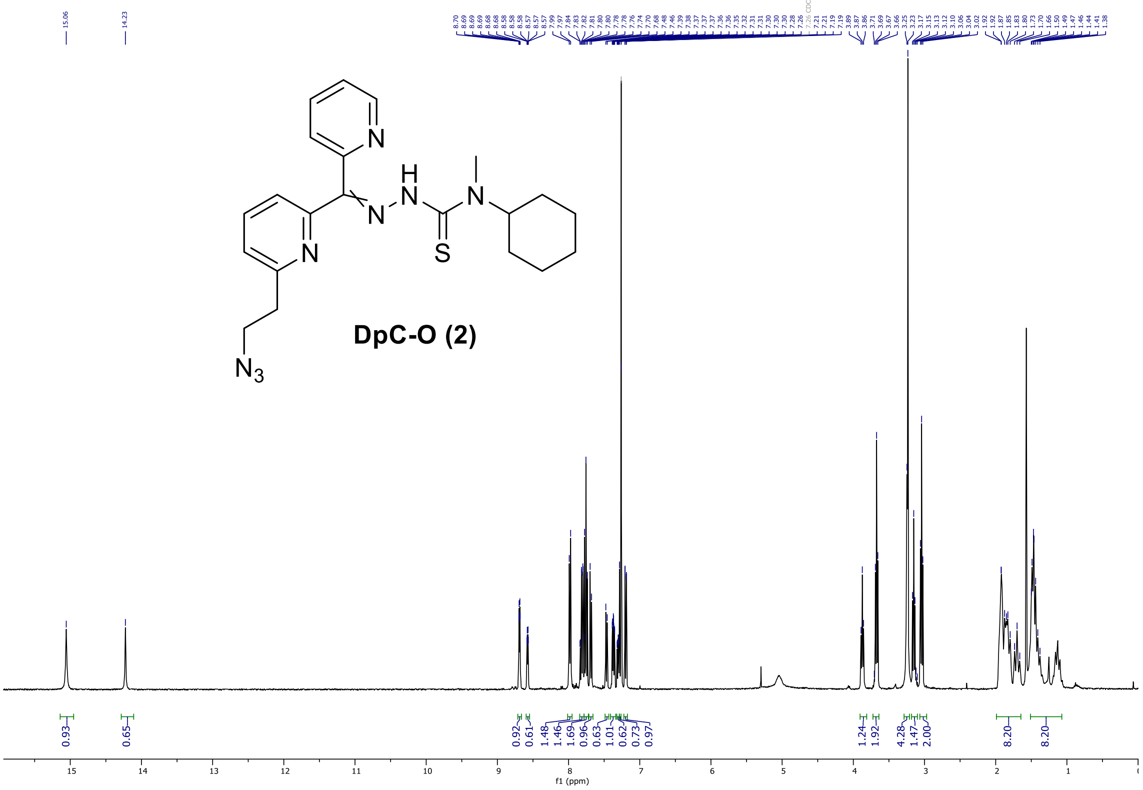

Figure S30: ^{1}H NMR Spectrum of **2.**

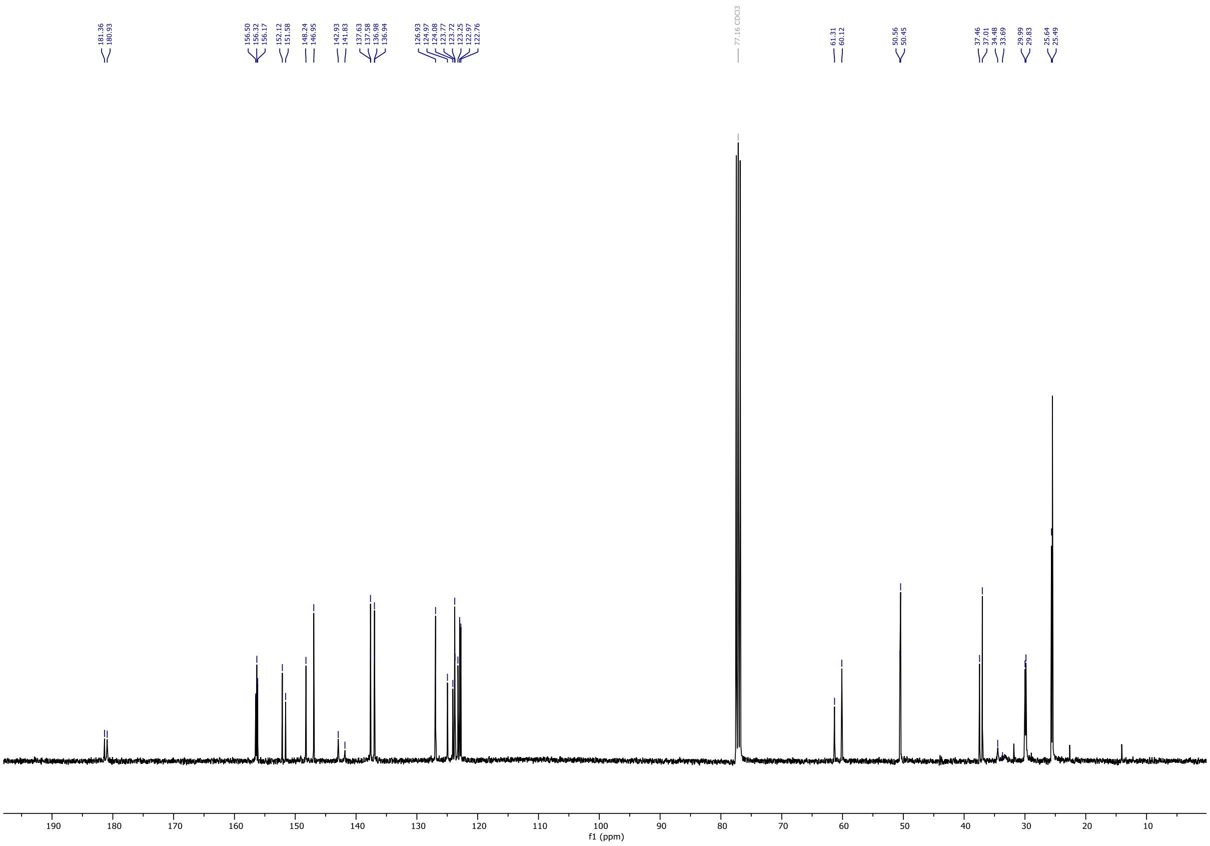

Figure S31: ^{13}C NMR Spectrum of **2.**

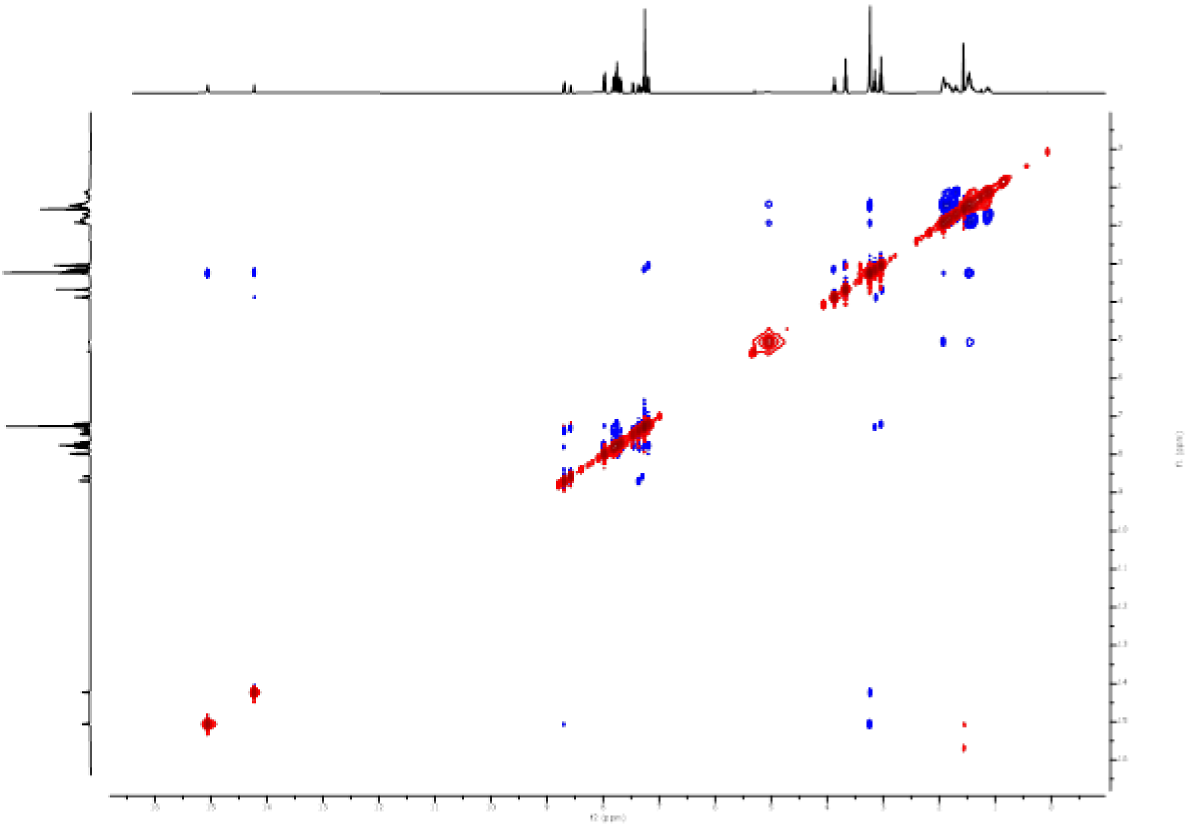

Figure S32: HH NOESY Spectrum of **2.**

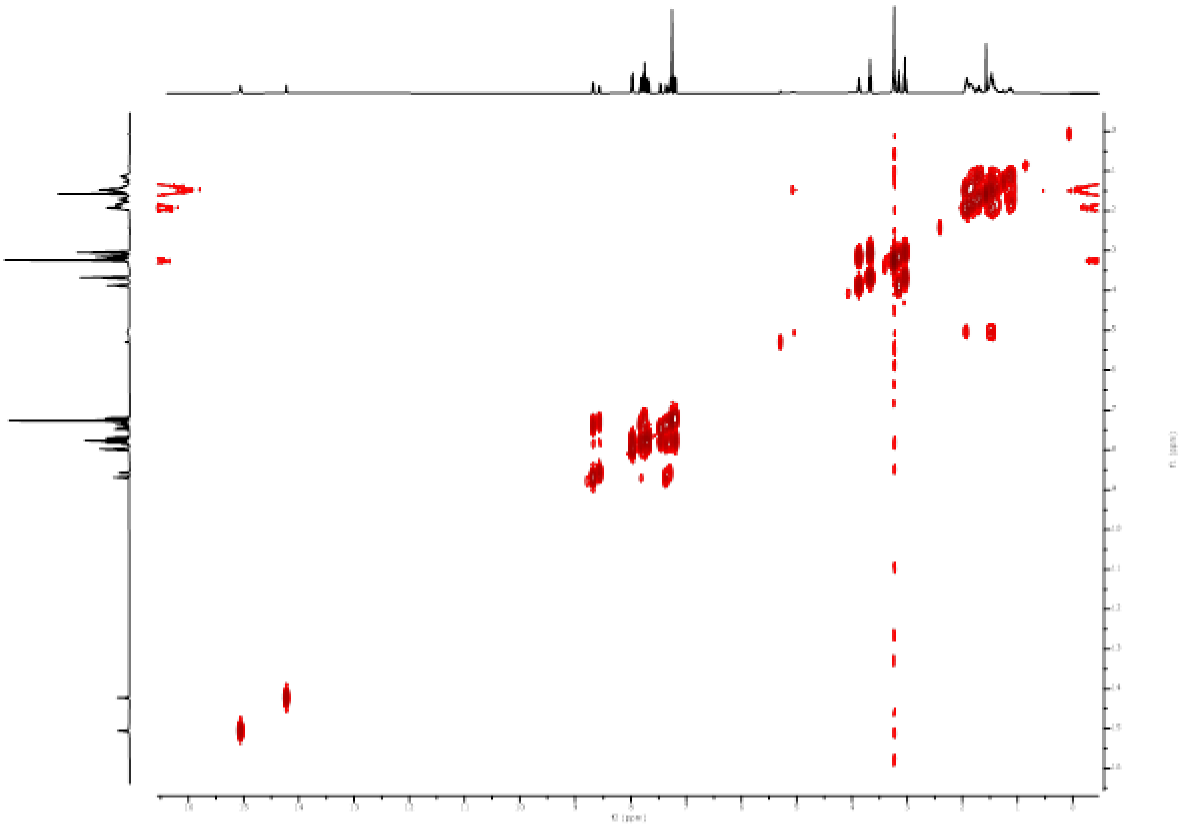

Figure S33: COSY Spectrum of **2.**

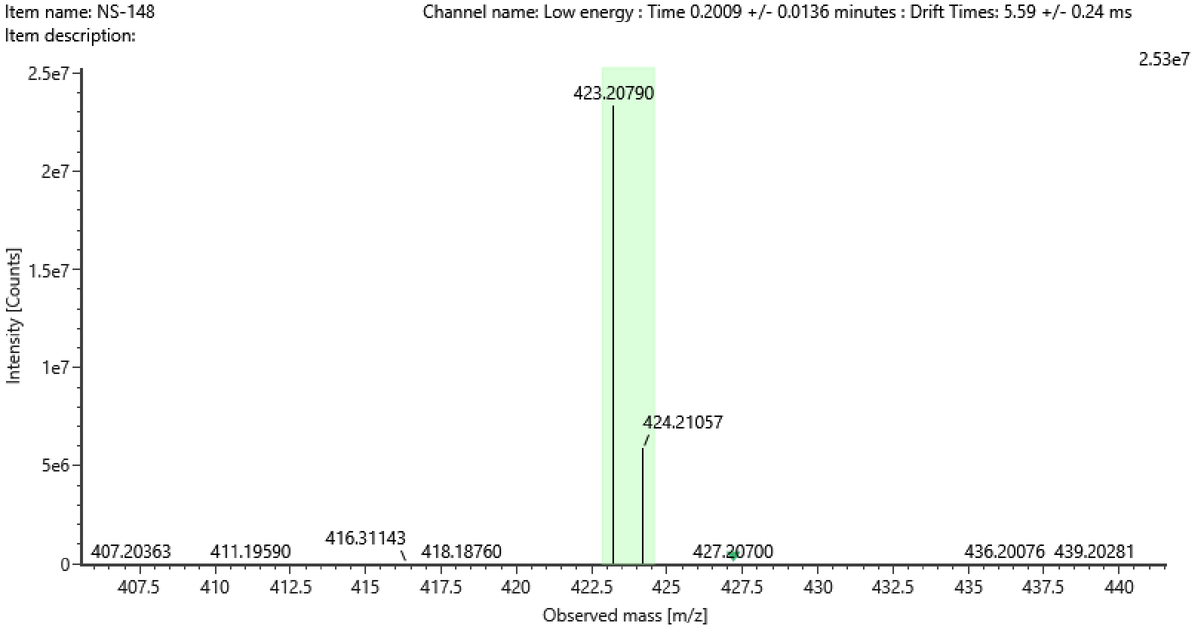

Figure S34: HRMS of **2.**

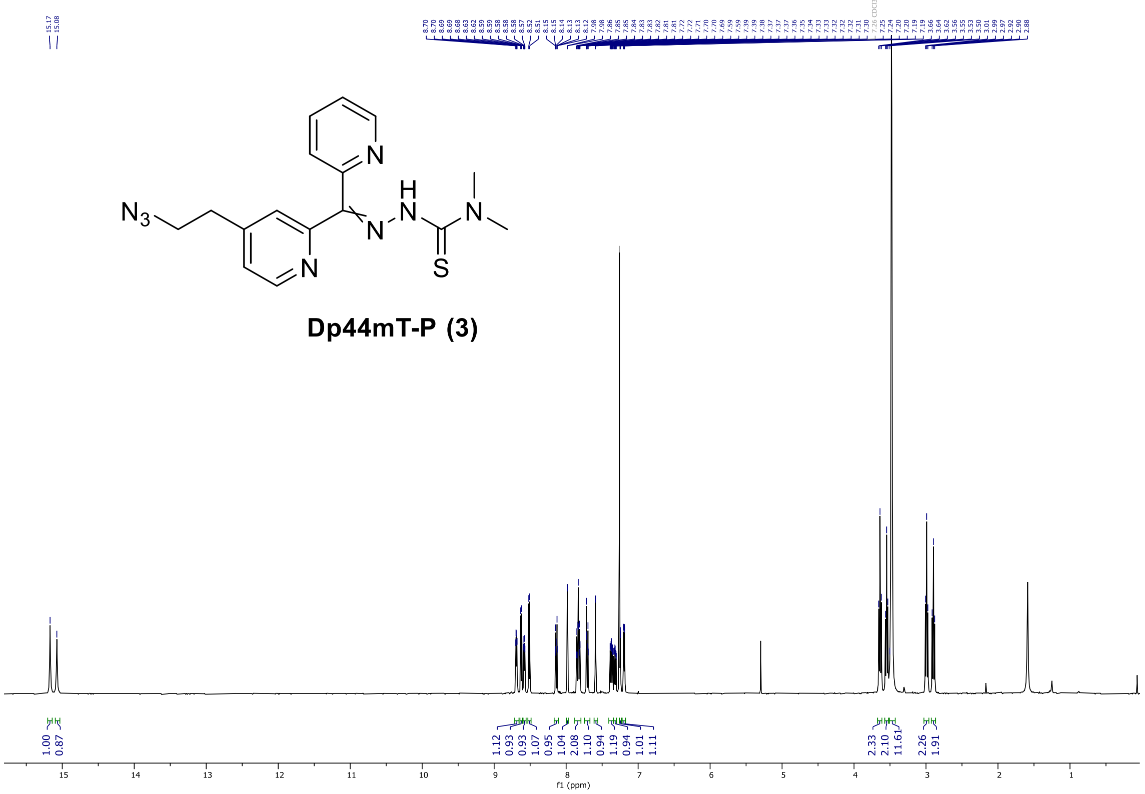

Figure S35: ^{1}H NMR Spectrum of **3.**

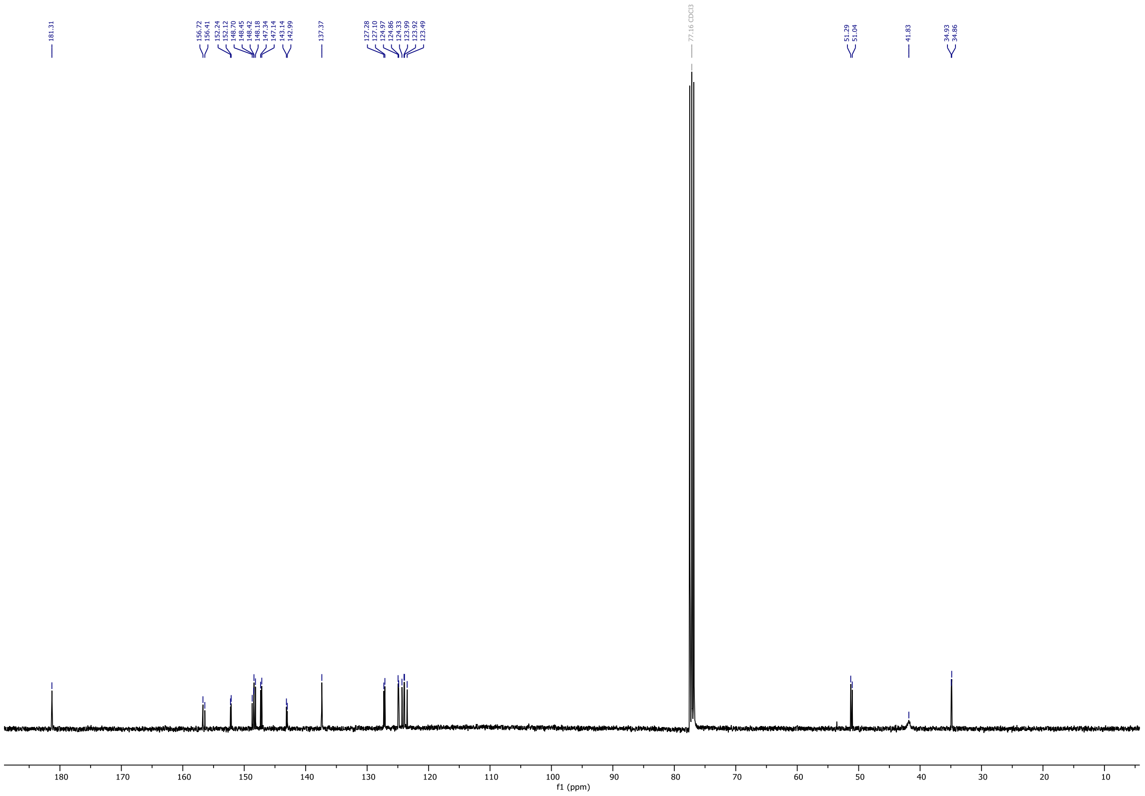

Figure S36: ^{13}C NMR Spectrum of **3.**

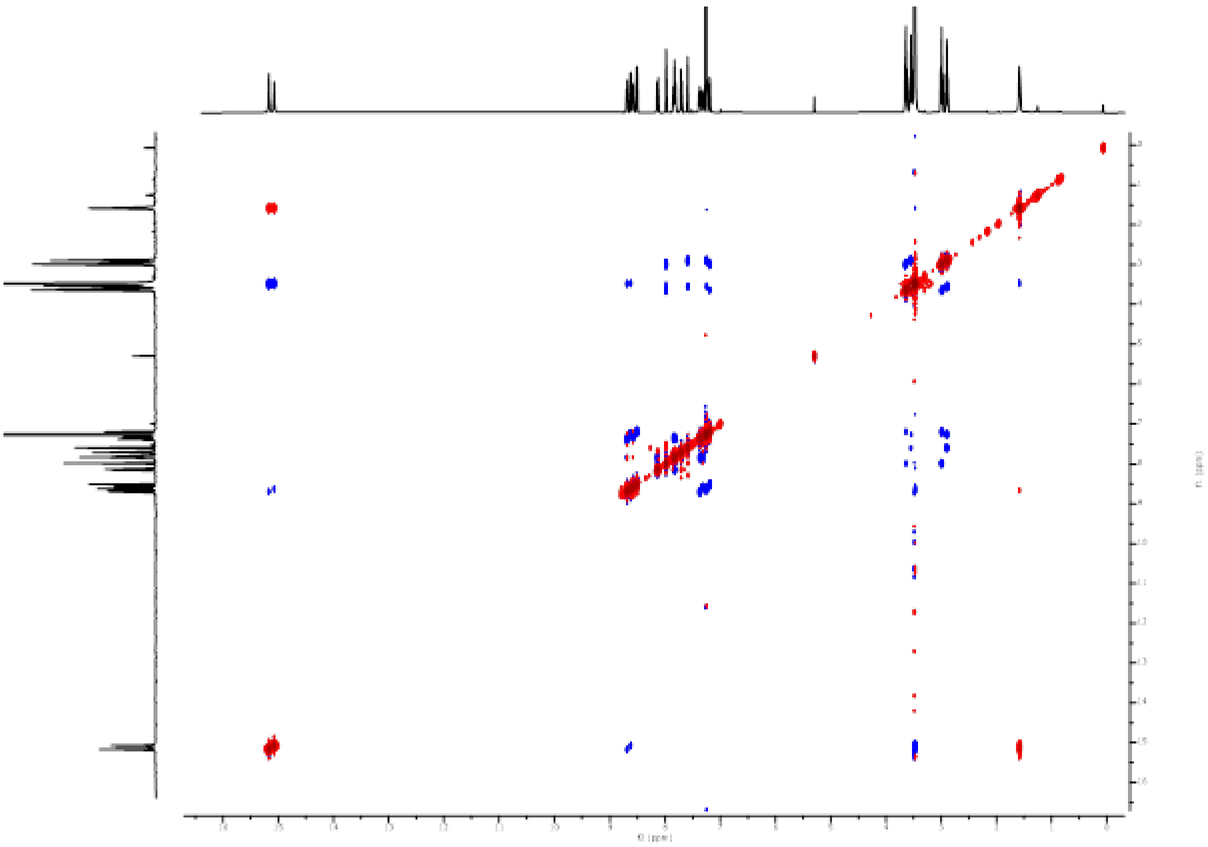

Figure S37: HH NOESY Spectrum of **3.**

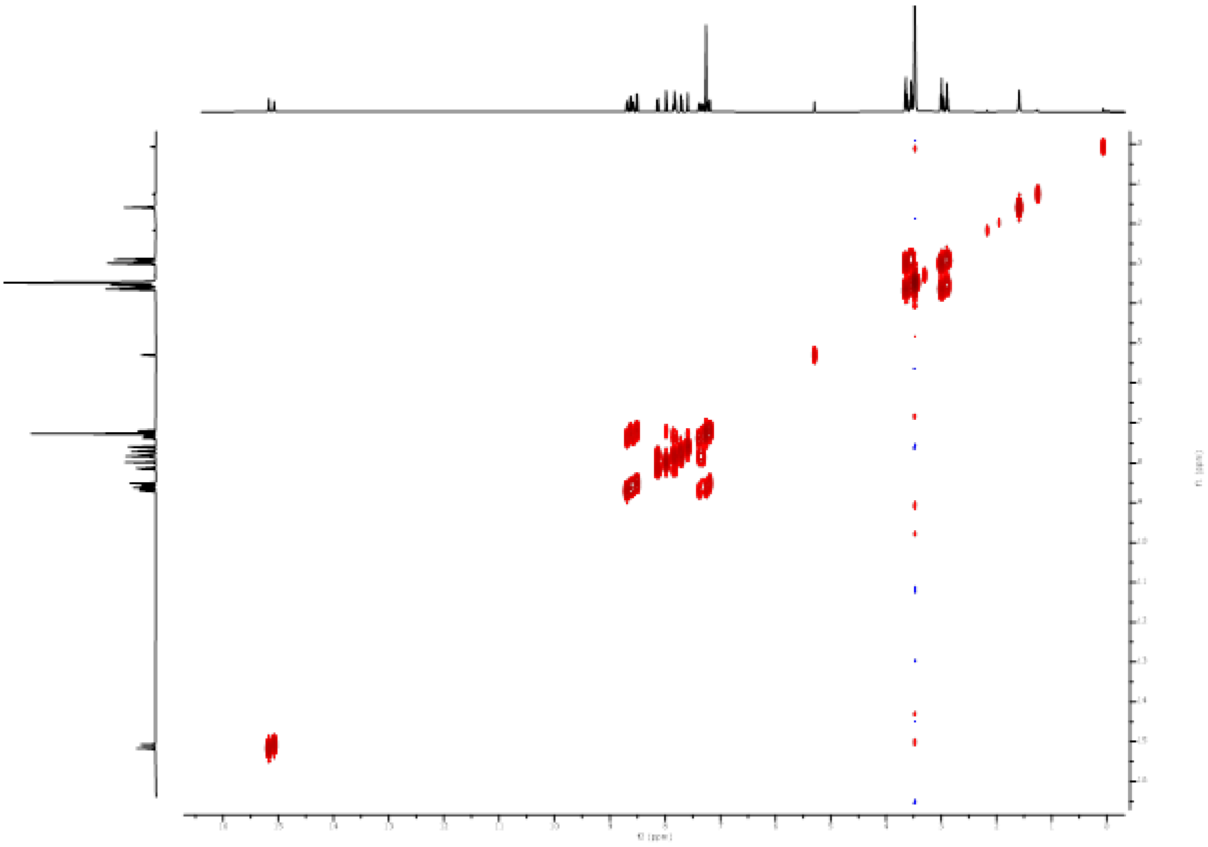

Figure S38: COSY Spectrum of **3.**

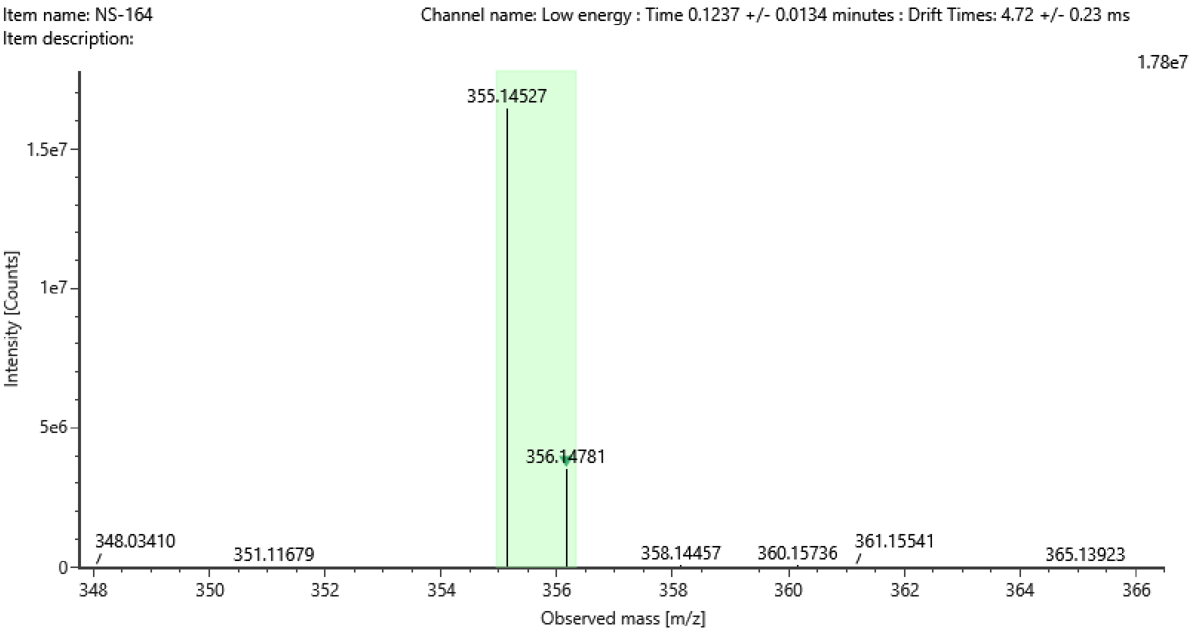

Figure S39: HRMS of **3.**

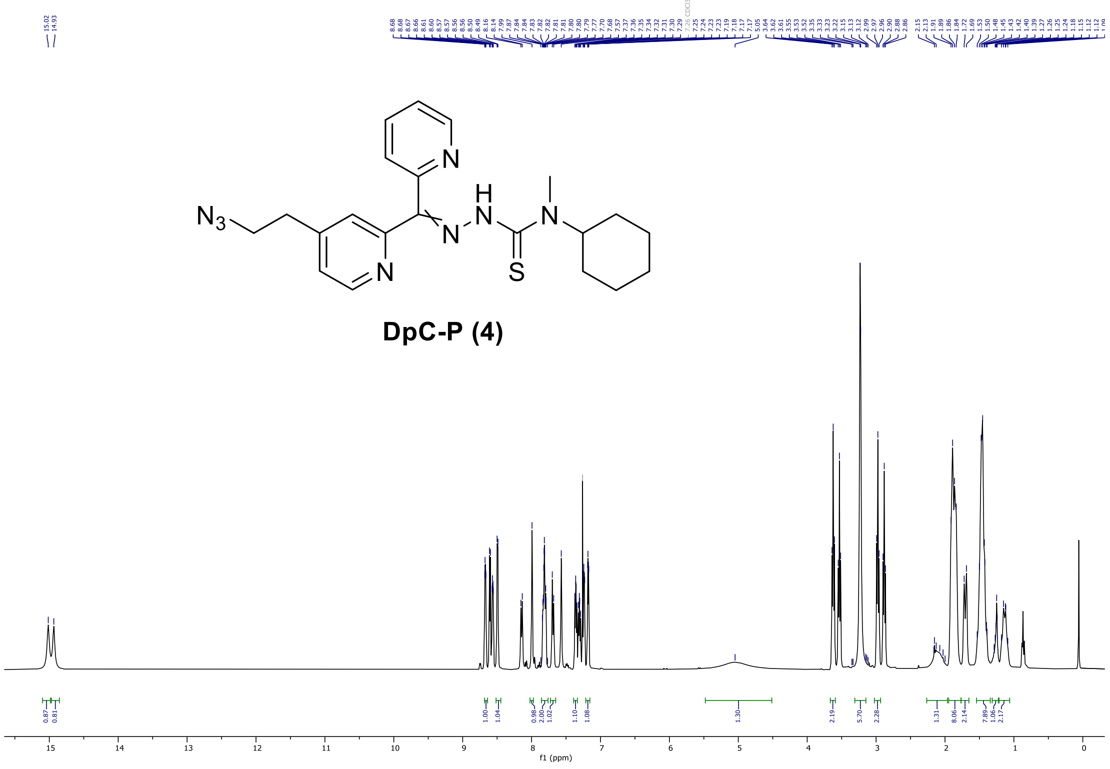

Figure S40: ^{1}H NMR Spectrum of **4.**

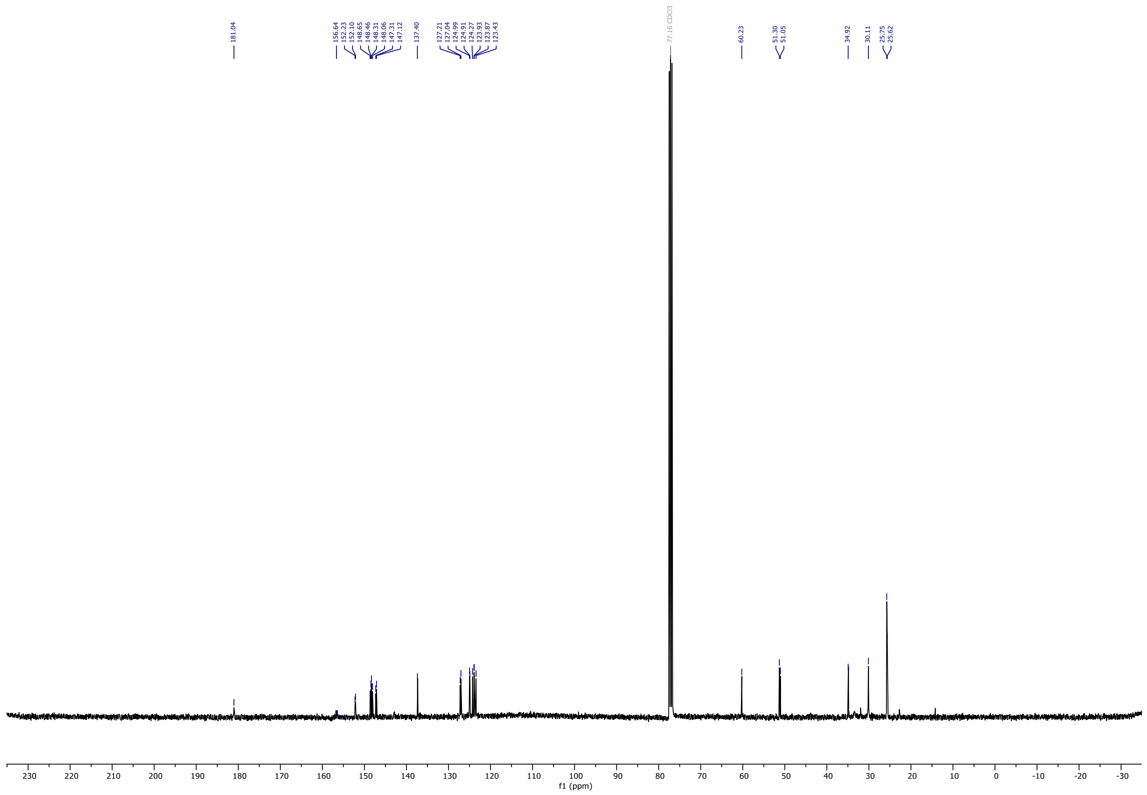

Figure S41: ^{13}C NMR Spectrum of **4.**

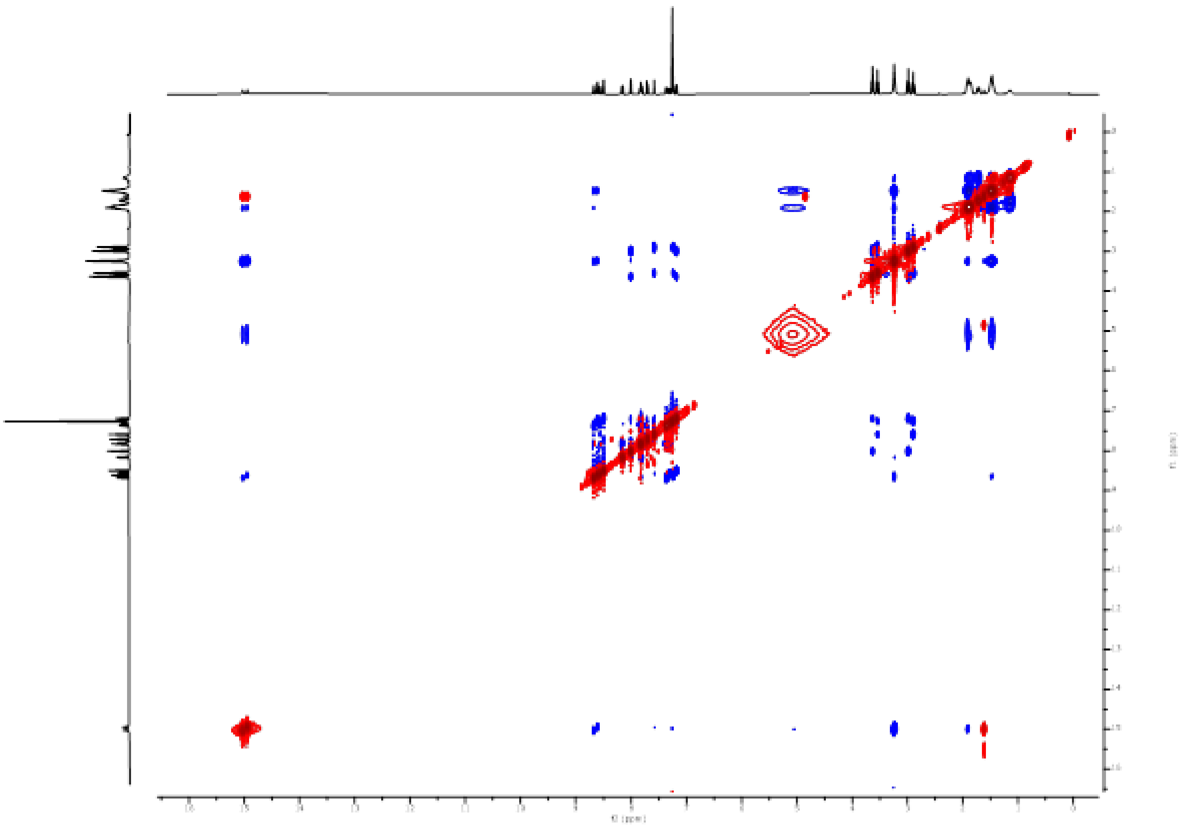

Figure S42: HH NOESY Spectrum of **4.**

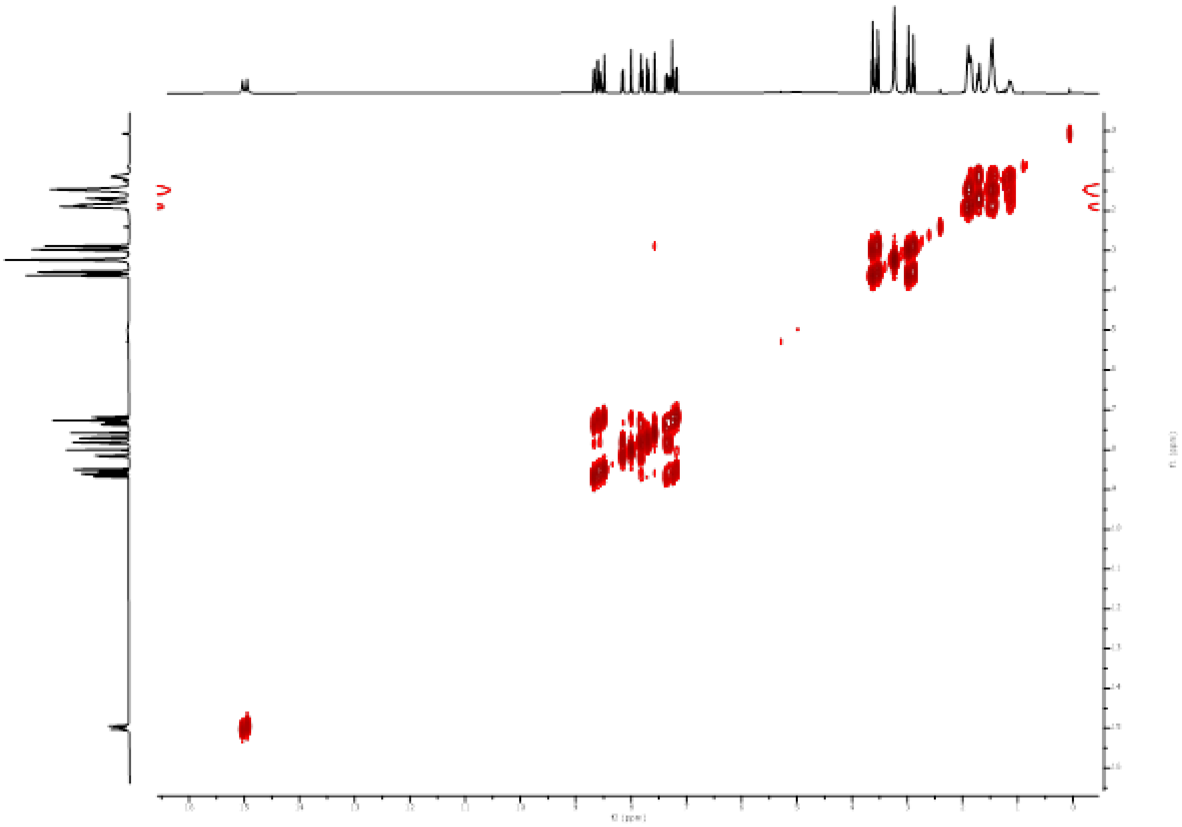

Figure S43: COSY Spectrum of **4.**

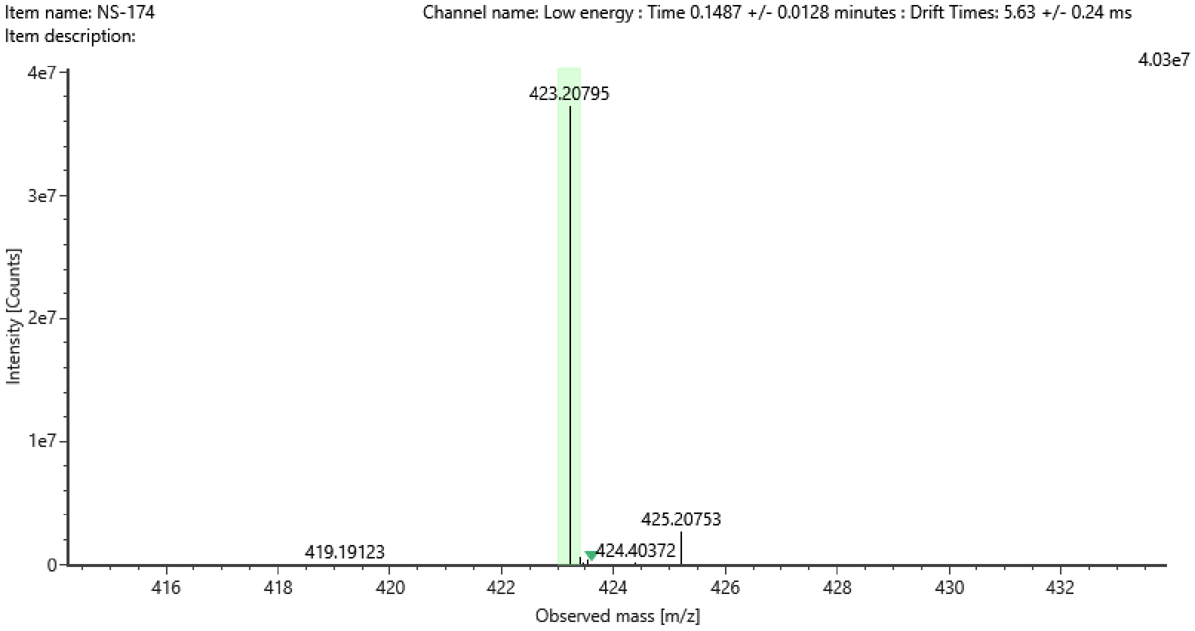

Figure S44: HRMS of **4.**

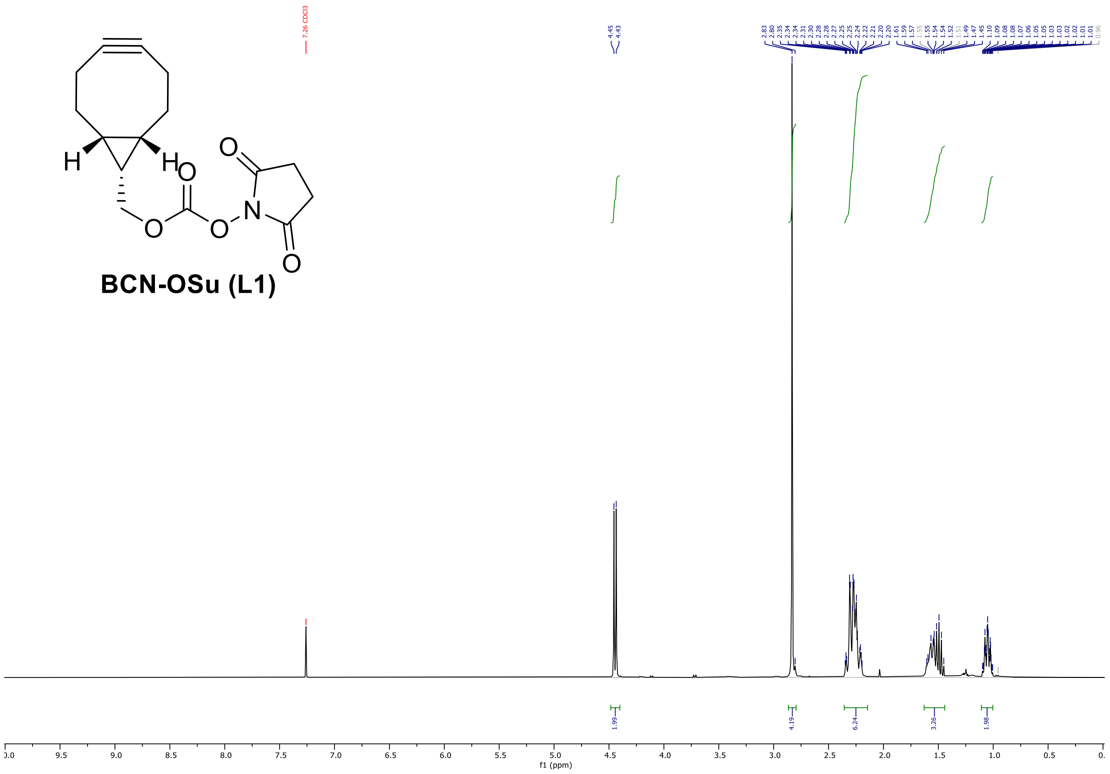

Figure S45: ^{1}H NMR spectrum of **L1.**

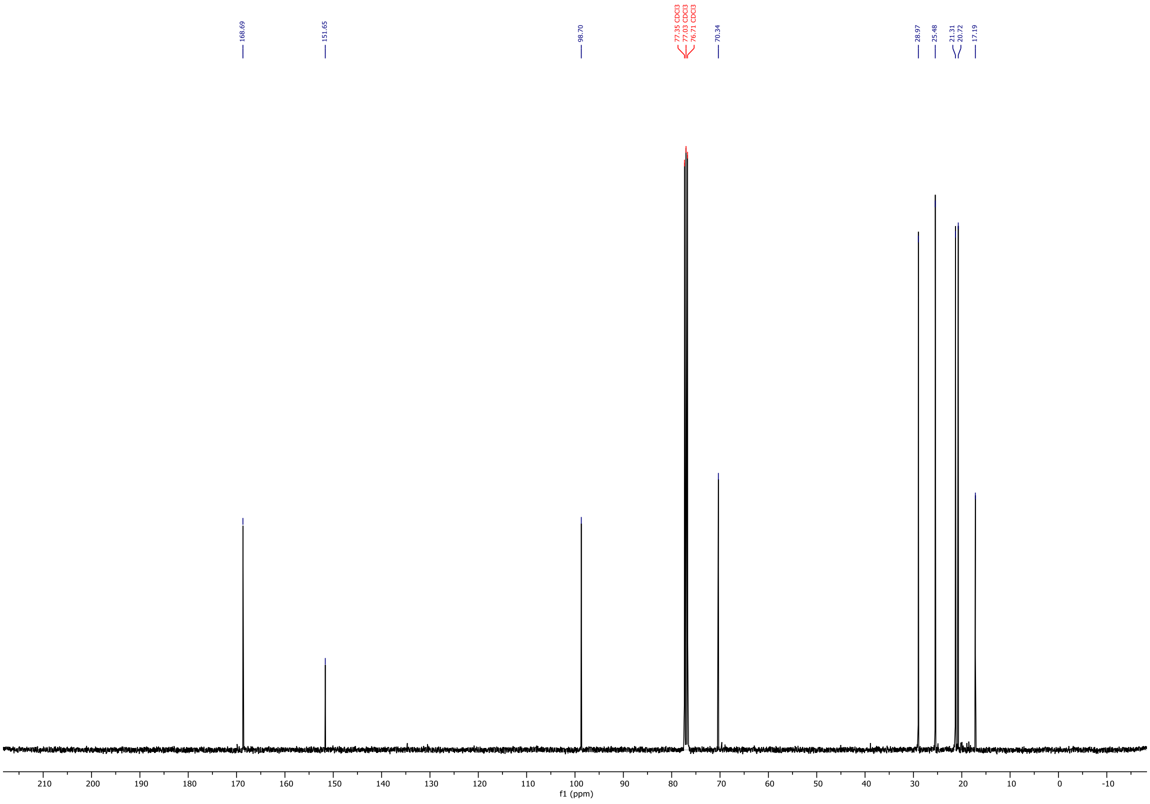

Figure S46: ^{13}C NMR spectrum of **L1.**

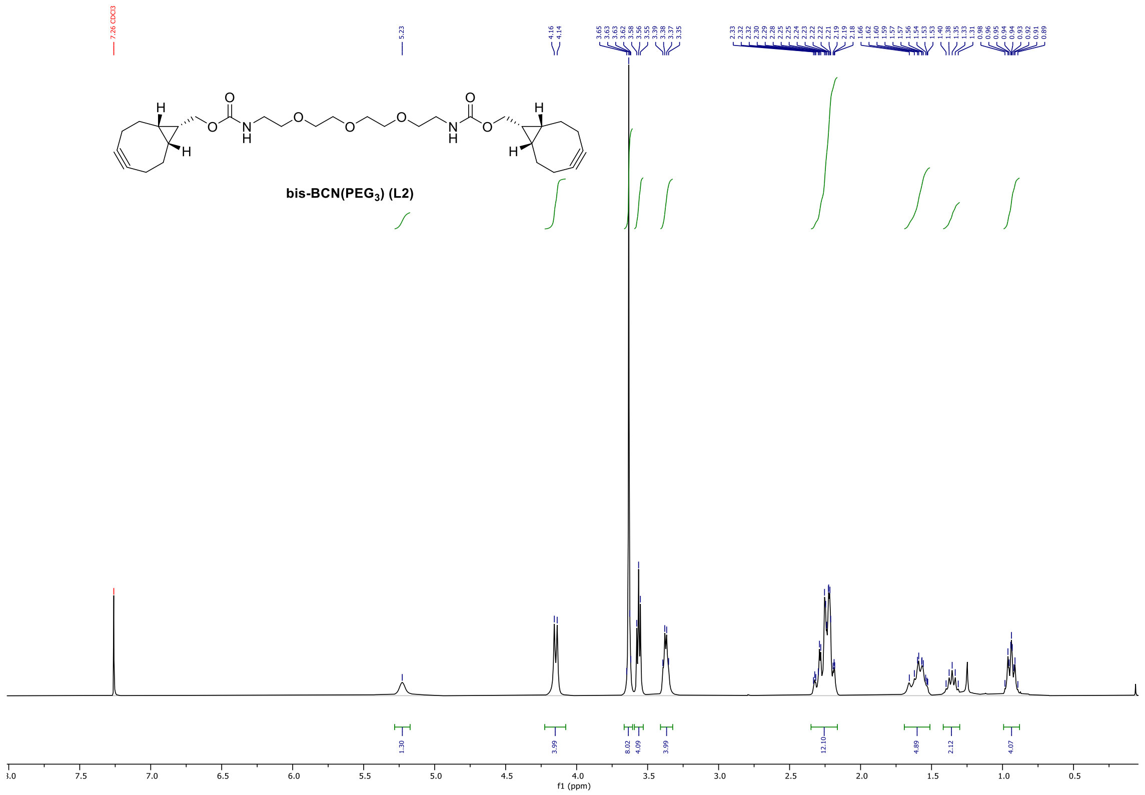

Figure S47: ^{1}H NMR spectrum of **L2.**

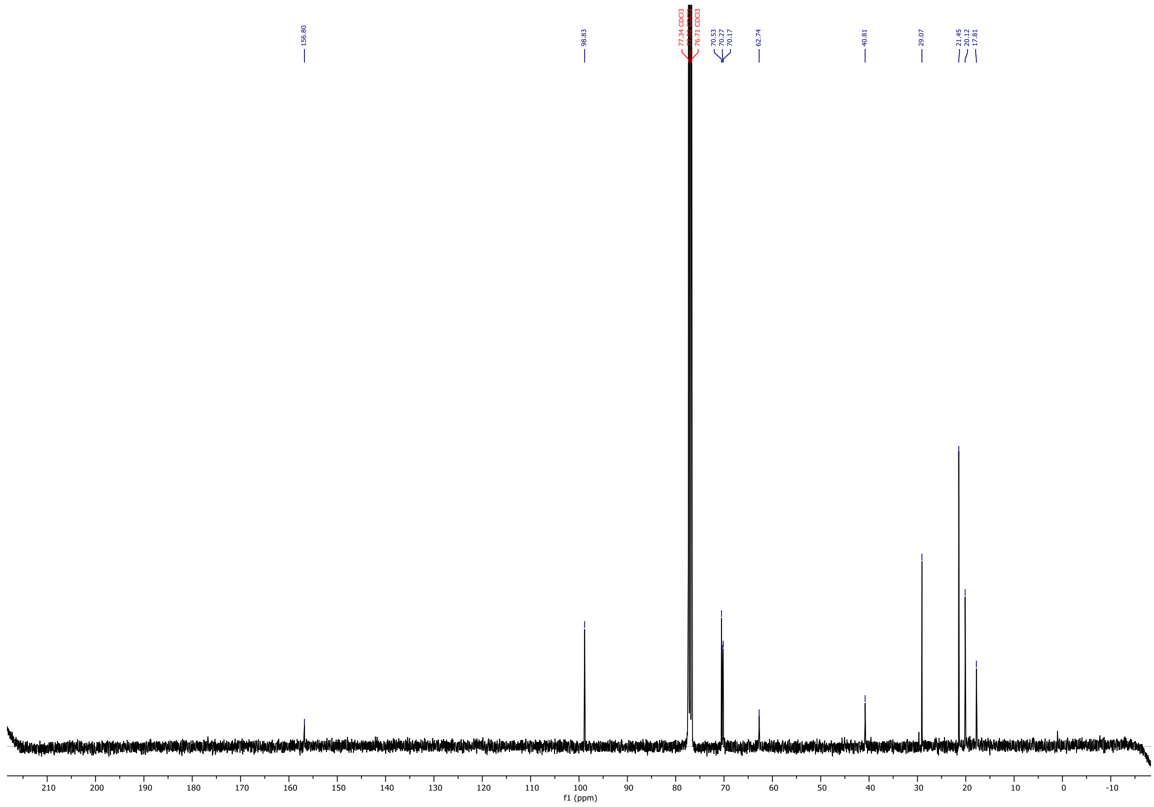

Figure S48: ^{13}C NMR spectrum of **L2.**

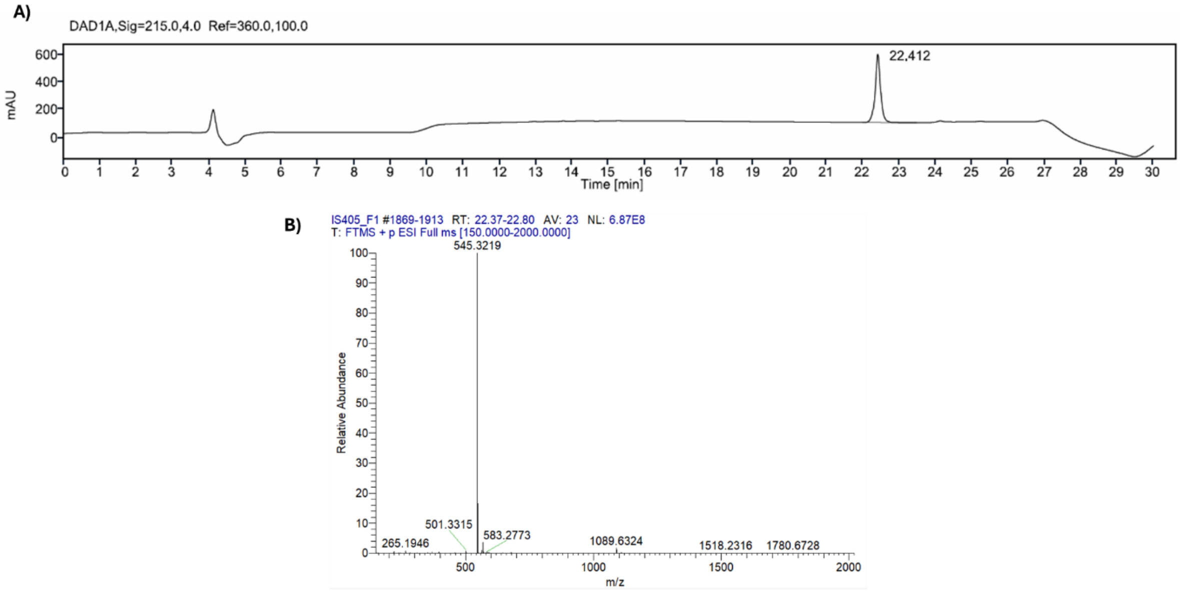

Figure S49: A) HPLC-UV trace and B) MS trace of bis-BCN(PEG_3).

5. Metal binding profiles

UV/Vis absorption spectra were recorded on a Jasco V-670 spectrophotometer operated at ambient temperature in single-use UV-cuvettes (1 cm; BRAND). Appropriate blank measurements of the respective solvents were recorded and subtracted from the resulting spectra.

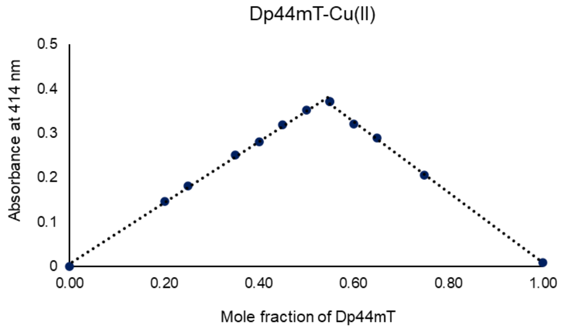

Figure S50: Job's plots of the absorbance at 422 nm and 427 nm of Dp44mT and DpC, respectively, with $CuSO_4$. A maximum in the absorption intensity is observed at mole fraction = 0.5, indicating that a 1:1 binding of DpT with Cu(II) is preferred.

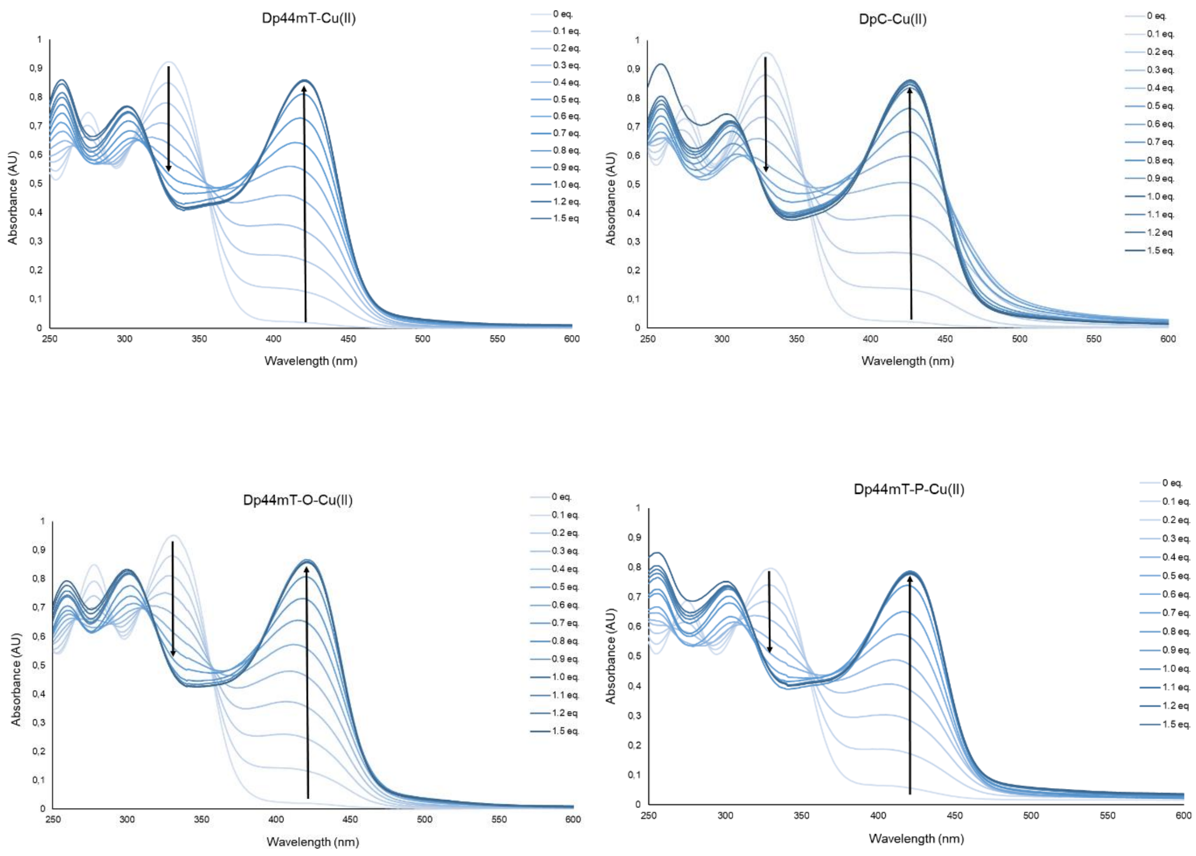

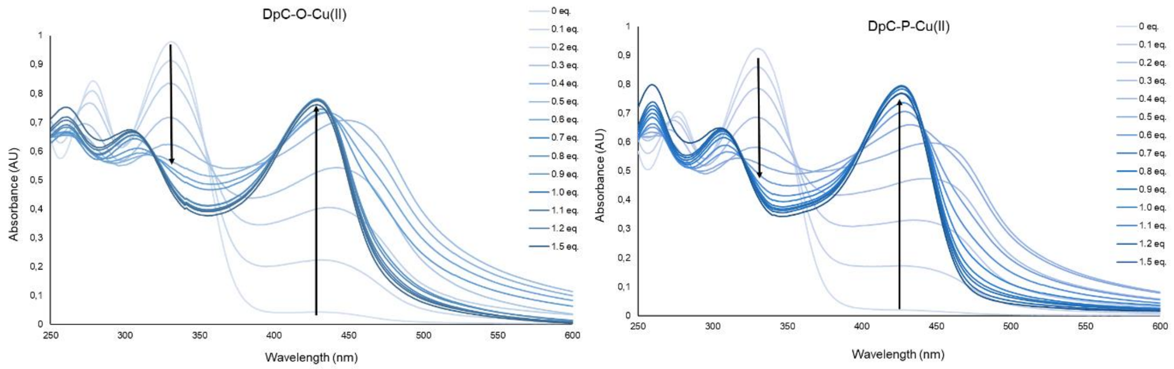

Figure S51: UV/Vis absorption profiles acquired by titrating $CuSO_4$ to 50 µM DpTs in 75 mM Tris-HCl (H_2O), pH 7.4.

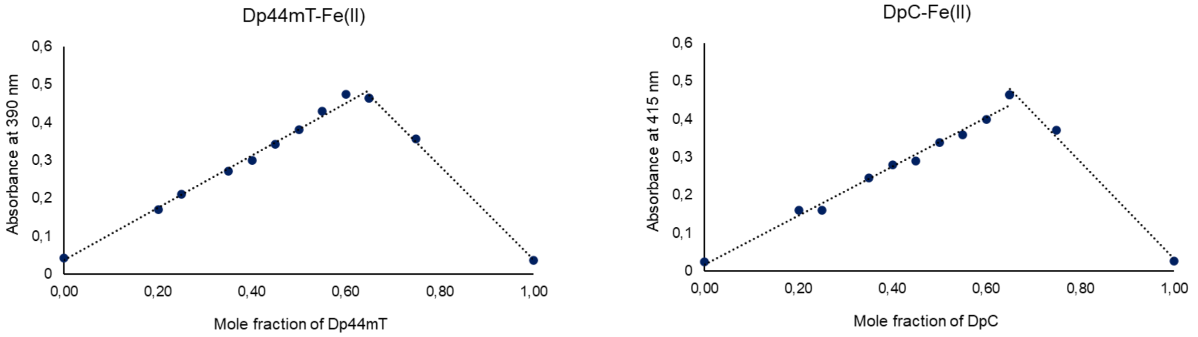

Figure S52: Job's plots of the absorbance at 400 nm and 425 nm of Dp44mT and DpC, respectively, with $FeCl_2$. A maximum in the absorption intensity is observed at mole fraction = 0.65, indicating that a 2:1 binding of DpT with Fe(II) is preferred.

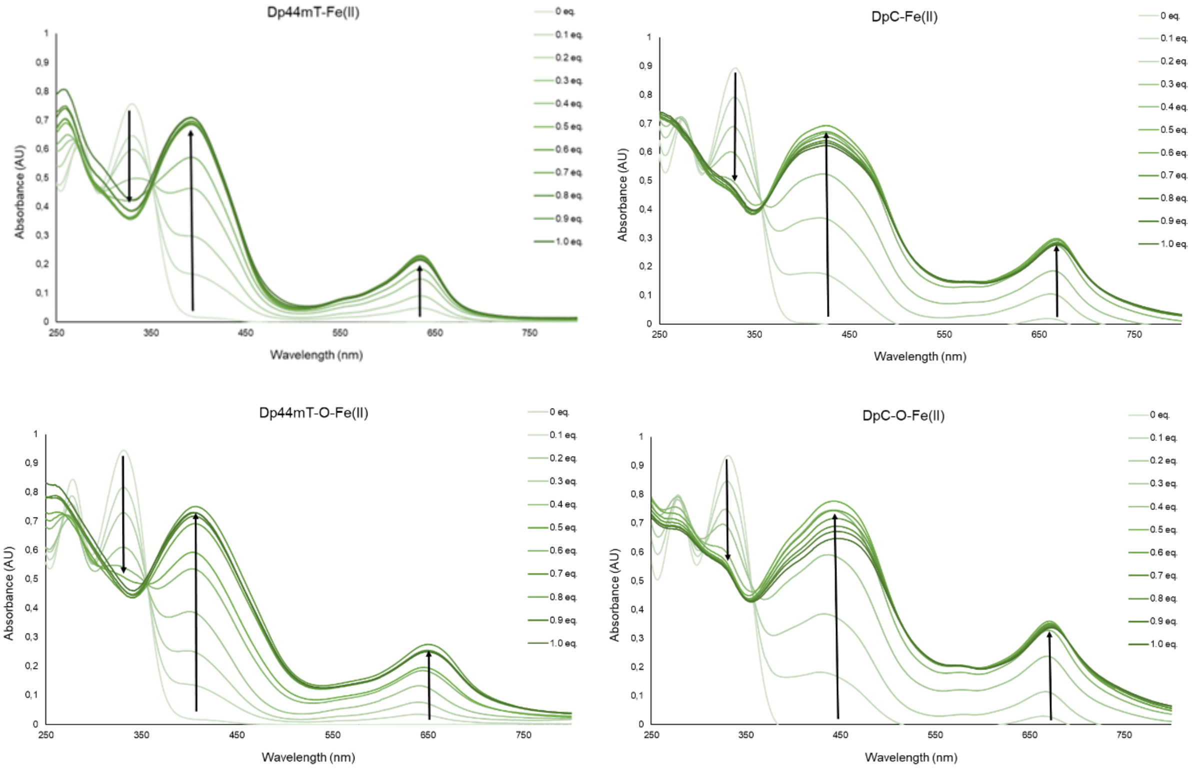

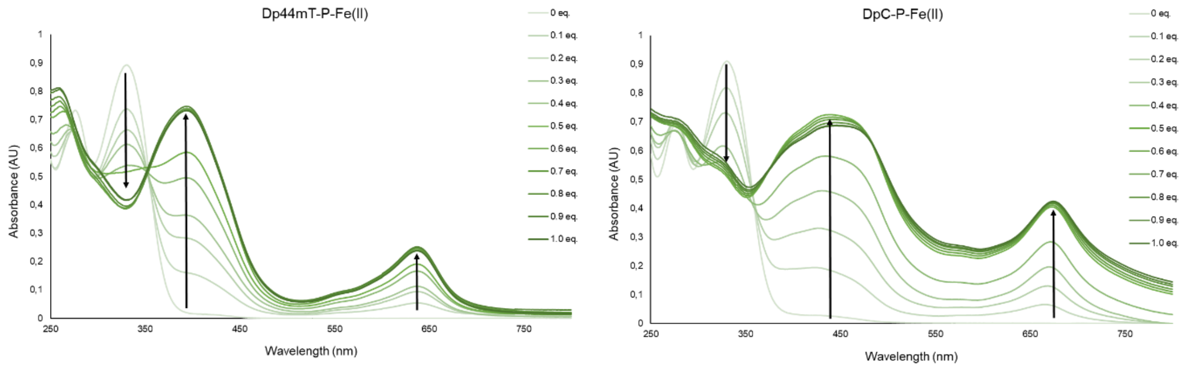

Figure S53: UV/Vis absorption profiles acquired by titrating $FeCl_2$ to 50 µM DpTs in 75 mM Tris-HCl (H_2O), pH 7.4.

6. Antibody modifications and bioconjugation

Enzymatic deglycosylation of trastuzumab

Trastuzumab (20.00 mg, 38.40 mg/mL in PBS pH 7.4) was incubated with PNGase F (24.8 µL, 12500 units) at 37 °C. After overnight incubation, the antibody was dialyzed (thrice to PBS pH 5.5) and concentrated to 56.61 mg/mL. Mass spectrometric analysis of a sample after FabRICATOR treatment showed the mass corresponding to Fc portion of the expected product (observed mass 23,788 Da, Fig. S54D).

Cetuximab (3.00 mg, 29.77 mg/mL in PBS pH 7.4) was incubated with PNGase F (7.5 µL, 3750 units) at 37 °C. After overnight incubation, the antibody was dialyzed (thrice to PBS pH 5.5) and concentrated to 37.31 mg/mL. Mass spectrometric analysis of a sample after FabRICATOR treatment showed the mass corresponding to Fc portion of the expected product (observed mass 23,787 and 23915 Da, Fig. S54G). The mass at 27,787 Da corresponds to the Fc part after lysine clipping, a common phenomenon observed during mAb production.[5]

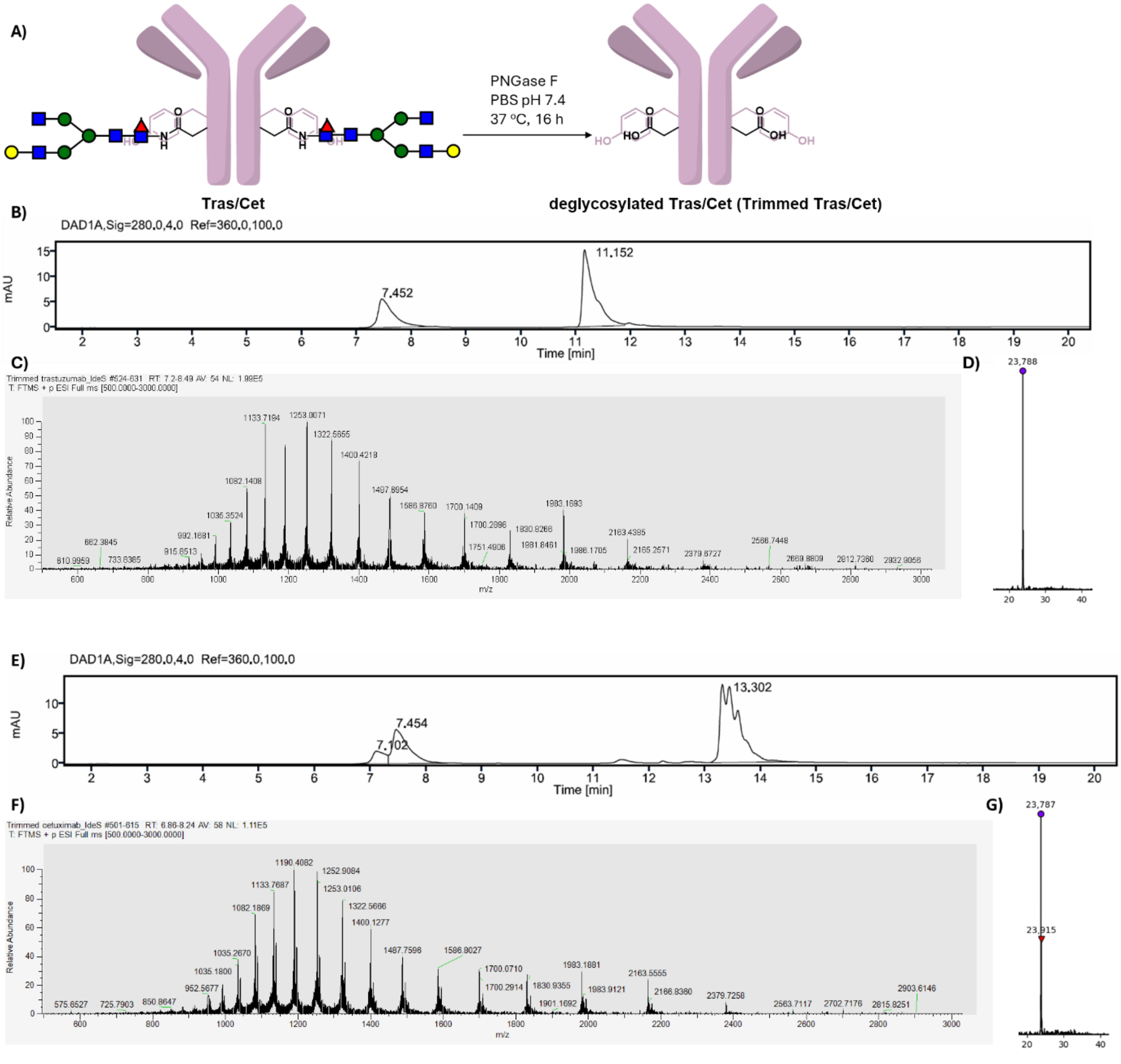

Figure S54: A) Schematic representation of deglycosylation of Tras/Cet using PNGase F to produce **Trimmed Tras/Cet**. B) HPLC trace of FabRICATOR digested **Trimmed Tras**, with Fc at t_R 7.4 min and F(ab')2 at t_R 11 min. C) Mass spectrum of the Fc of **Trimmed Tras**. D) Deconvoluted mass spectrum of Fc

portion (the peak eluting at t_R 7.4 min in panel B). E) HPLC trace of FabRICATOR digested **Trimmed Cet**, with Fc at t_R 7.1 min and 7.5 min and F(ab')2 at t_R 13.3 min. F) Mass spectrum of the Fc of **Trimmed Cet**. G) Deconvoluted mass spectrum of Fc portion (the peak eluting at t_R 7.0−8 min in panel E).

Conjugation of Trimmed Tras with bis-BCN(PEG$_3$)

Deglycosylated trastuzumab (**Trimmed Tras**) (141.3 µL, 56.61 mg/mL, 8.00 mg in PBS pH 5.5) was incubated with bis-BCN(PEG$_3$) (**L2**, 30.0 µL, 10.00 mg/mL in DMSO, 10.0 eq.) and mushroom tyrosinase (705.4 µL, 10.00 mg/mL in phosphate buffer pH 6.0, 1.0 eq.) at 4 °C. After overnight incubation, the product (**T1**) was purified using protein A purification and buffer exchanged to PBS pH 5.5. RP-LC-MS analysis of the FabRICATOR digested product was performed as described above, indicated clean conversion, and showed one major Fc region of the product (observed mass 24,346 Da) corresponding to the expected product.

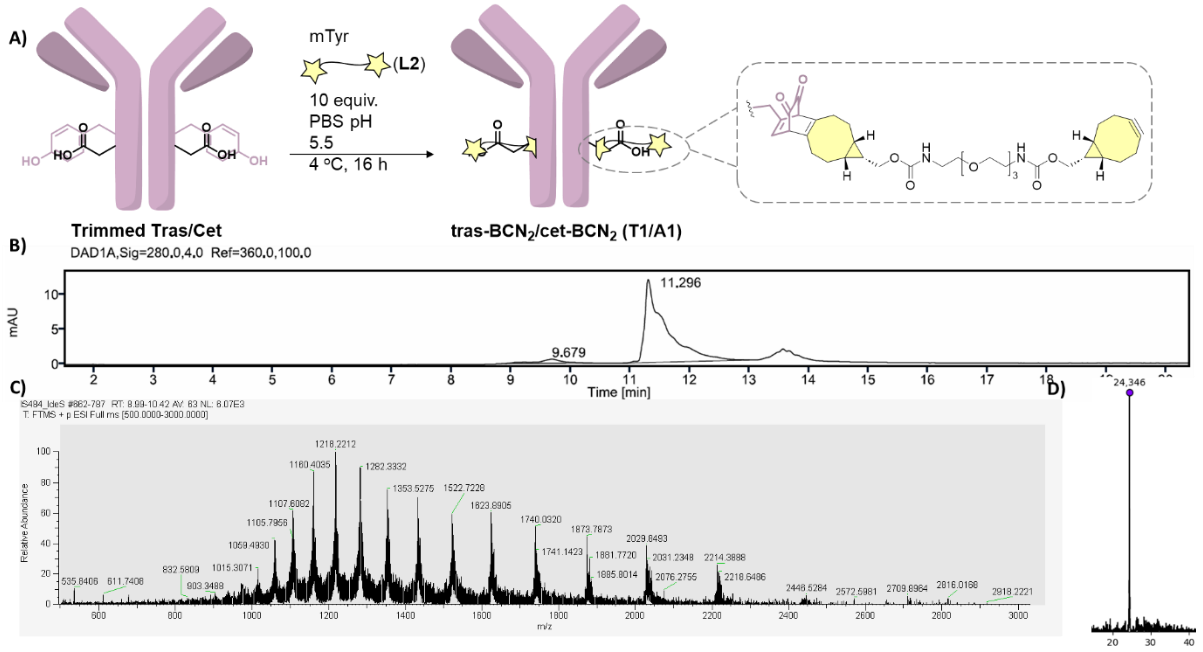

Figure S55: A) Schematic representation of generation of BCN-functionalized trastuzumab (tras-BCN$_2$ **T1**) via SPOCQ reaction. B) HPLC trace of FabRICATOR digested tras-BCN$_2$, with Fc at t_R 9.7 min and F(ab')2 at t_R 11.3 min. C) Mass spectrum of the Fc of tras-BCN$_2$ (**T1**). D) Deconvoluted mass spectrum of Fc portion (the peak eluting at t_R 9.7 min in panel B).

Conjugation of Trimmed Cet with bis-BCN(PEG$_3$)

Deglycosylated cetuximab (**Trimmed Cet**) (82.9 µL, 42.22 mg/mL, 3.50 mg in PBS pH 5.5) was incubated with bis-BCN(PEG$_3$) (**L2**, 12.7 µL, 10.00 mg/mL in DMSO, 10.0 eq.) and mushroom tyrosinase (299.2 µL, 10.00 mg/mL in phosphate buffer pH 6.0, 1.00 eq.) at 4 °C. After overnight incubation, the product (**A1**) was purified using protein A purification and buffer exchanged to PBS pH 5.5. RP-LC-MS analysis of the FabRICATOR digested product was performed as described above, indicated clean conversion, and showed one major Fc region of the product (observed mass 24,345 Da) corresponding to the expected product.

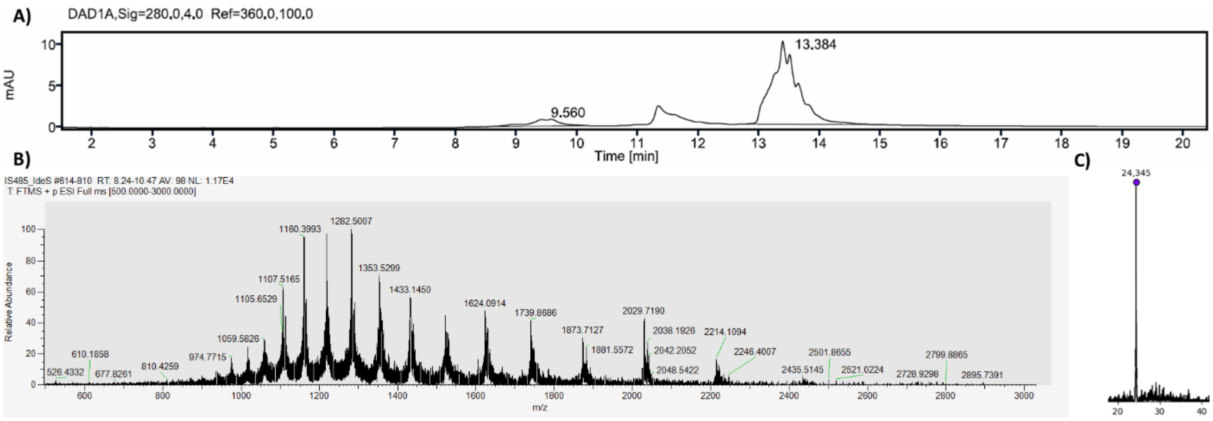

Figure S56: A) HPLC trace of FabRICATOR digested cet-BCN$_2$ (**A1**), with Fc at t_R 9.6 min and F(ab')2 at t_R 13.4 min. C) Mass spectrum of the Fc of cet-BCN$_2$ (**A1**). D) Deconvoluted mass spectrum of Fc portion (the peak eluting at t_R 9.6 min in panel A).

Generation of trastuzumab-based DAR 2 ADC constructs

Tras-BCN$_2$ **T1** (232.3 µL, 10.33 mg/mL, 2.40 mg in PBS pH 5.5) was incubated with azido-DpT (**1**, **2,** **3** or **4**) (25.00 mg/mL in DMSO, 6.0 eq.) at 4 °C. After overnight incubation, the product (**5**, **6**, **7** or **8**) was spin filtered through Amicon spin filter 50 kD MWCO to PBS pH 5.5. RP-LC-MS analysis of the FabRICATOR digested product indicated clean conversion and showed one major Fc region of the product (observed mass 24,700 Da for **5** and **7**, and 24,770 Da for **6** and **8**) corresponding to the expected product.

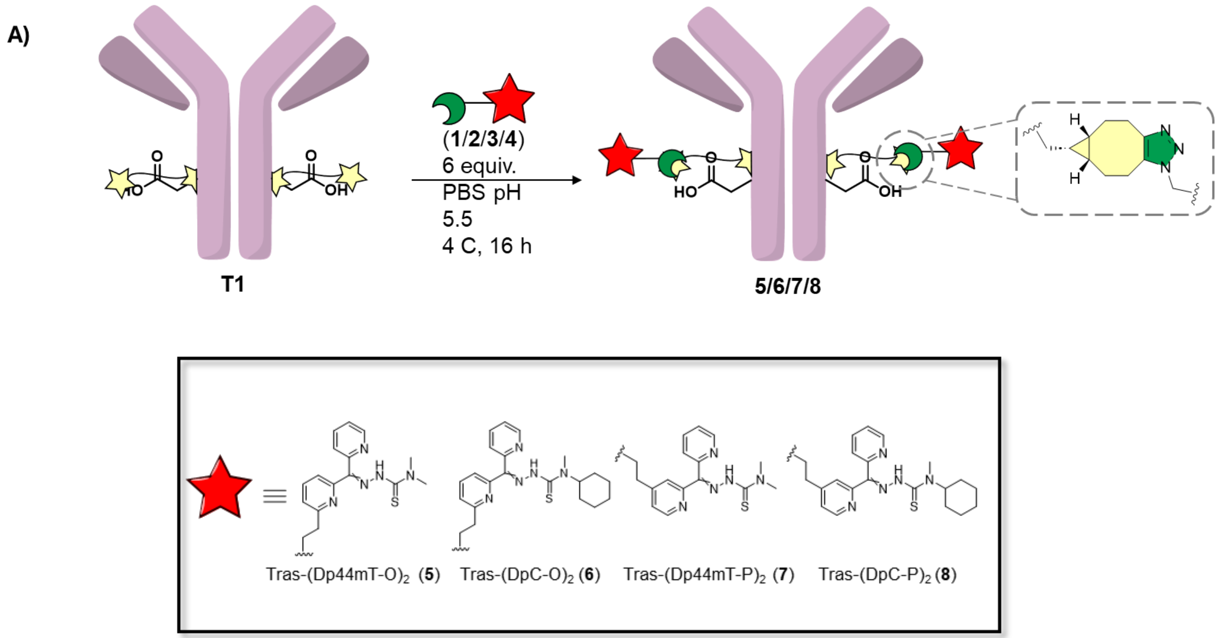

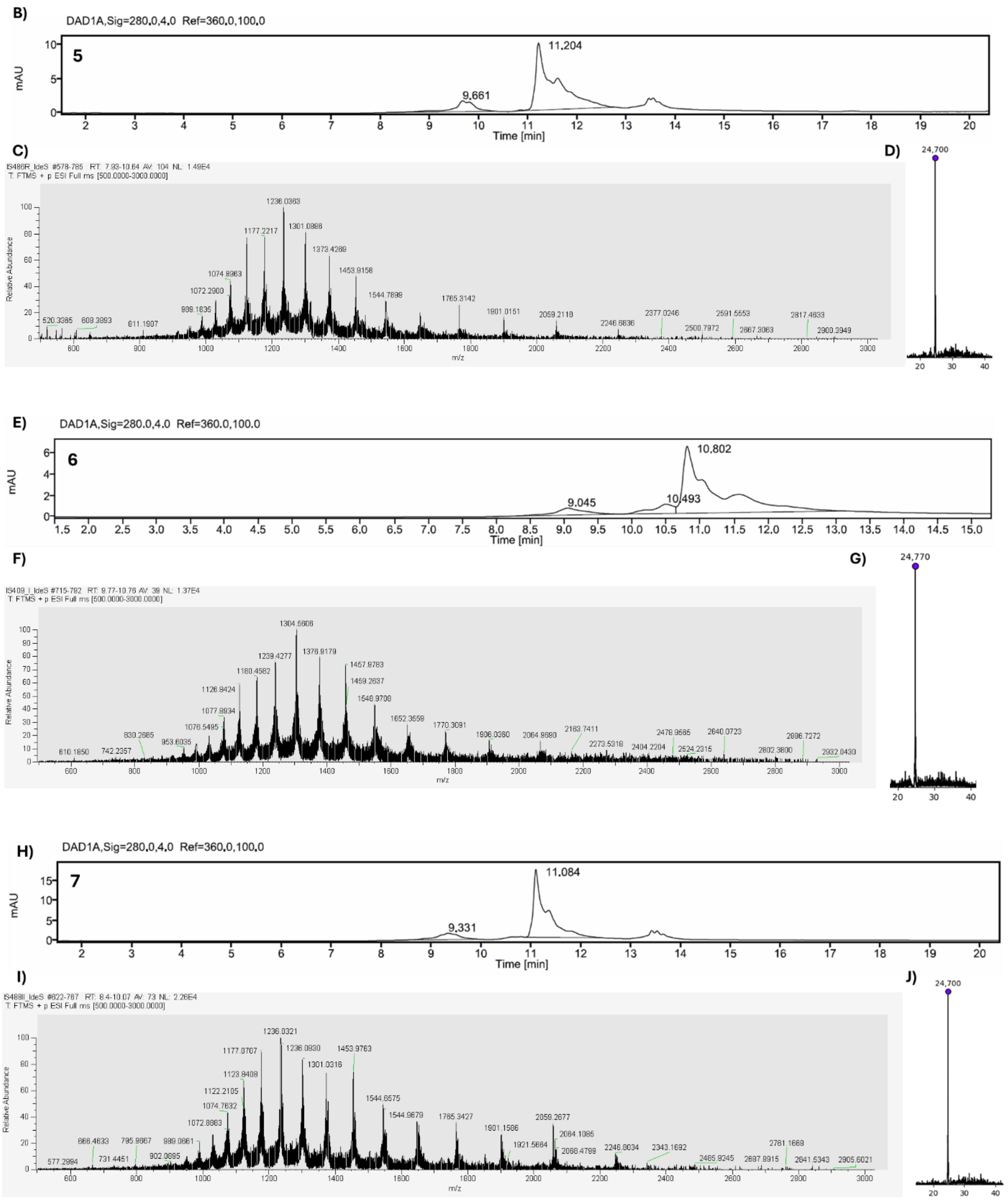

B)
DAD1A,Sig=280,0,4,0 Ref=360,0,100,0
5
11,204
9,661
mAU
Time [min]
C)
D)
24,700
1236.0363
1177.2217
1301.0888
1373.4289
1453.9158
1074.8963
1072.2900
1544.7899
1765.3142
Relative Abundance
m/z
E)
DAD1A,Sig=280,0,4.0 Ref=360.0,100.0
6
10,802
9,045
10,493
mAU
Time [min]
F)
G)
24,770
1304.5606
1376.9179
1239.4277
1457.9783
1180.4582
1126.8424
1548.9708
1077.8934
1652.3559
1770.3091
Relative Abundance
m/z
H)
DAD1A,Sig=280,0,4,0 Ref=360,0,100,0
7
11,084
9,331
mAU
Time [min]
I)
J)
24,700
1236.0321
1177.0707
1453.9763
1301.0316
1123.8408
1122.2105
1544.6575
1074.7632
1072.8863
1765.3427
2059.2677
Relative Abundance
m/z

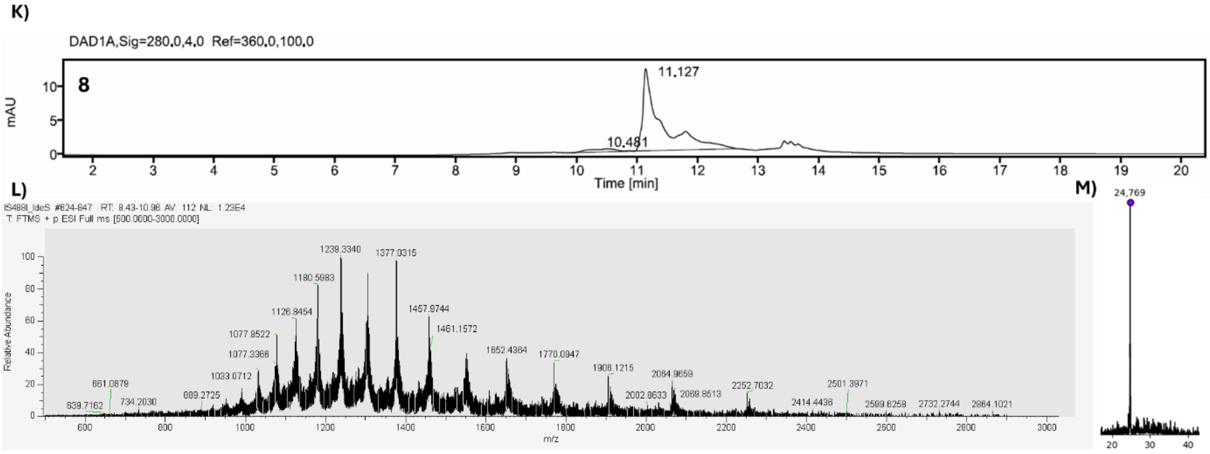

Figure S57: A) Schematic representation of generation of DAR2 ADC via SPAAC reaction and structures of the payloads. B) HPLC trace of FabRICATOR digested Tras-(Dp44mT-O)$_2$ (**5**), with Fc at t_R 9.7 min and F(ab')2 at t_R 11.2 min. C) Mass spectrum of the Fc of **5**. D) Deconvoluted mass spectrum of Fc portion (the peak eluting at t_R 9.7 min in panel B). E) HPLC trace of FabRICATOR digested Tras-(DpC-O)$_2$ (**6**), with Fc at t_R 10.5 min and F(ab')2 at t_R 10.8 min. F) Mass spectrum of the Fc of **6**. G) Deconvoluted mass spectrum of Fc portion (the peak eluting at t_R 10.5 min in panel E). H) HPLC trace of FabRICATOR digested Tras-(Dp44mT-P)$_2$ (**7**), with Fc at t_R 9.3 min and F(ab')2 at t_R 11.1 min. I) Mass spectrum of the Fc of **7**. J) Deconvoluted mass spectrum of Fc portion (the peak eluting at t_R 9.3 min in panel H). K) HPLC trace of FabRICATOR digested Tras-(DpC-P)$_2$ (**8**), with Fc at t_R 10.5 min and F(ab')2 at t_R 11.1 min. L) Mass spectrum of the **8**. M) Deconvoluted mass spectrum of Fc portion (the peak eluting at t_R 10.5 min in panel K).

Note: tras-(DpC-*ortho*)$_2$ was measured on a different MAbPac column. Hence, the difference in the retention time of F(ab')$_2$.

Generation of trastuzumab-(TAMRA)$_2$

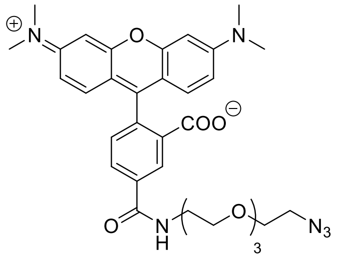

TAMRA-PEG$_3$-azide

Tras-BCN$_2$ **T1** (39.5 µL, 37.99 mg/mL, 1.50 mg in PBS pH 5.5) was incubated with TAMRA-PEG$_3$-azide (10.00 mg/mL in DMSO, 6.0 eq.) at 4 °C. After overnight incubation, the product **9** was spin-filtered through an Amicon spin filter 50 kD MWCO to PBS pH 5.5. RP-LC-MS analysis of the FabRICATOR digested product showed one mass corresponding to the TAMRA-labeled Fc region of the expected product **9** (observed mass 24,977 Da).

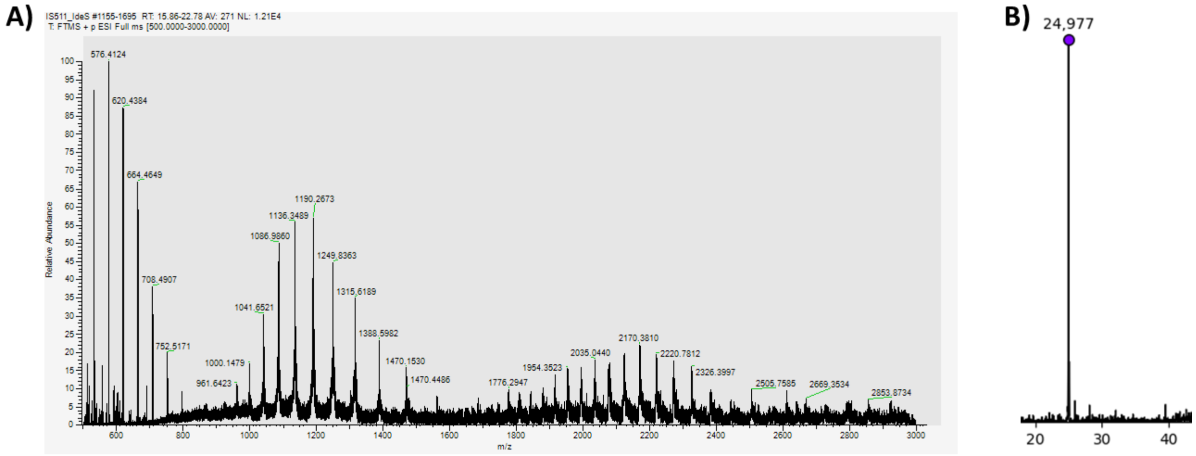

Figure S58: A) Mass spectrum of FabRICATOR digested tras-(TAMRA)$_2$ (**9**). The m/z range from 800-1600 belongs to the Fc regions whilst m/z from 1800 to 3000 belongs to the F(ab)'2. * Both the Fc and F(ab)'2 regions coeluted at the same retention time. B) Deconvoluted mass spectrum of Fc portion (m/z 800−1600).

Generation of cetuximab-based DAR 2 ADC construct

Cet-BCN$_2$ **A1** (400 µL, 3.951 mg/mL, 1.58 mg in PBS pH 5.5) was incubated with azido-thiosemicarbazone (**a**) (25.00 mg/mL in DMSO, 6 eq.) at 4 °C. After overnight incubation, the product (**10**) was spin filtered through Amicon spin filter 50 kD MWCO to PBS pH 5.5. RP-LC-MS analysis of the FabRICATOR digested product indicated clean conversion and showed one major Fc region of the product (observed mass 24,701 Da) corresponding to the expected product **10**.

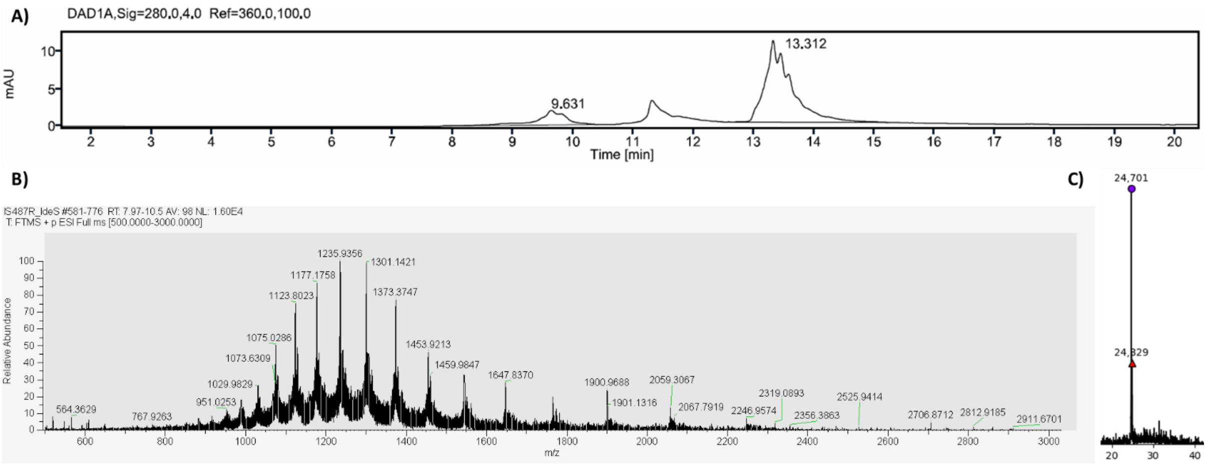

Figure S59: A) HPLC trace of FabRICATOR digested Cet-(Dp44mT-O)$_2$ (**10**), with Fc at t_R 9.6 min and F(ab')2 at t_R 13.3 min. B) Mass spectrum of the Fc of **9**. C) Deconvoluted mass spectru1m of Fc portion (the peak eluting at t_R 9.6 min in panel A).

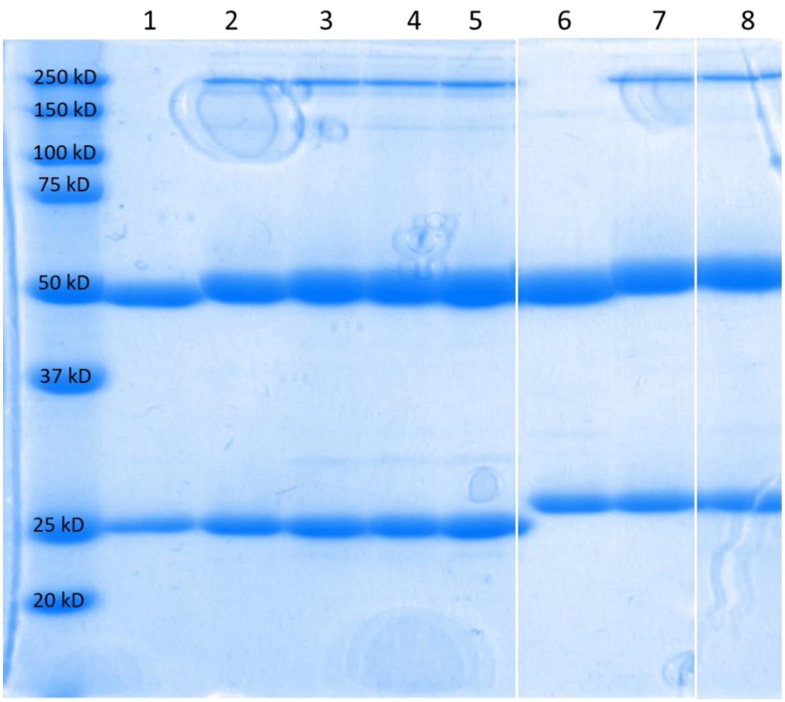

1. Deglycosylated trastuzumab
2. Tras-BCN_2
3. Tras-(DpC-*p*)$_2$
4. Tras-(Dp44mT-*o*)$_2$
5. Tras-(Dp44mT-*p*)$_2$
6. Deglycosylated cetuximab
7. Cet-BCN_2
8. Cet-(Dp44mT-*o*)$_2$

Figure S60: Reducing 12% SDS-PAGE of different constructs.

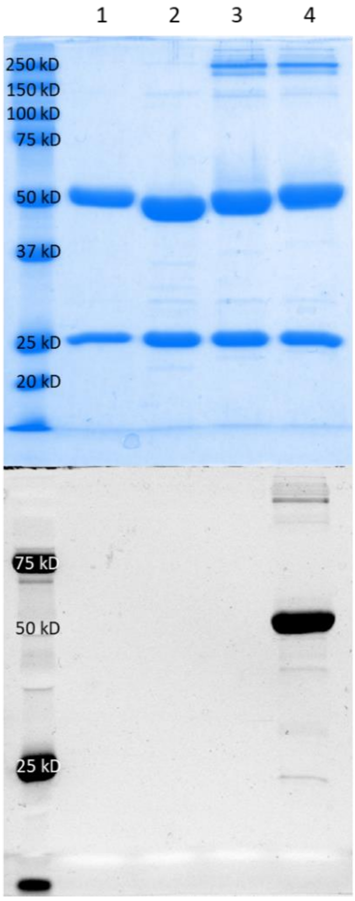

1. Trastuzumab
2. Deglycosylated trastuzumab
3. Tras-BCN_2
4. Tras-$TAMRA_2$

Figure S61: Reducing SDS-PAGE of TAMRA-labeled trastuzumab

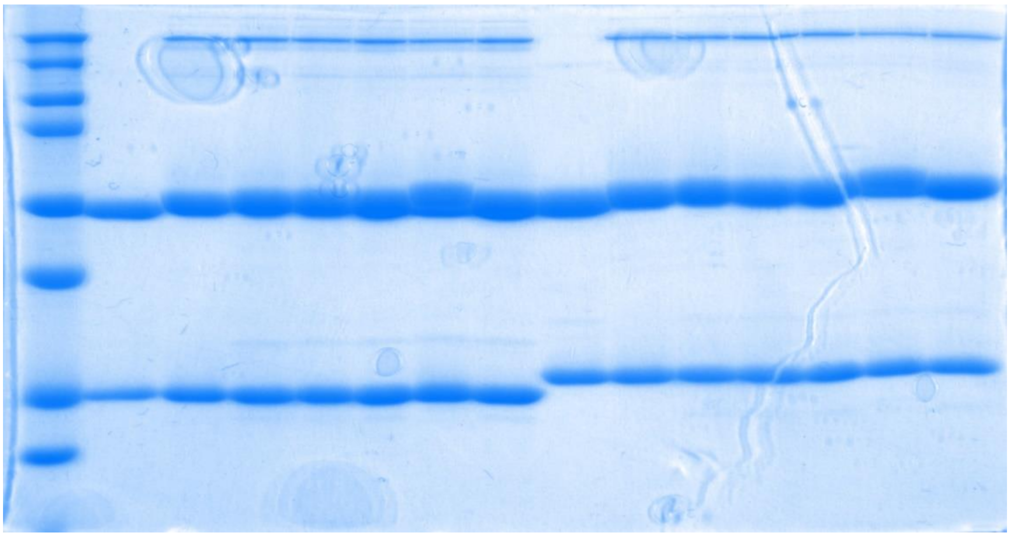

Figure S62: Full SDS-PAGE gel image of Figure S60

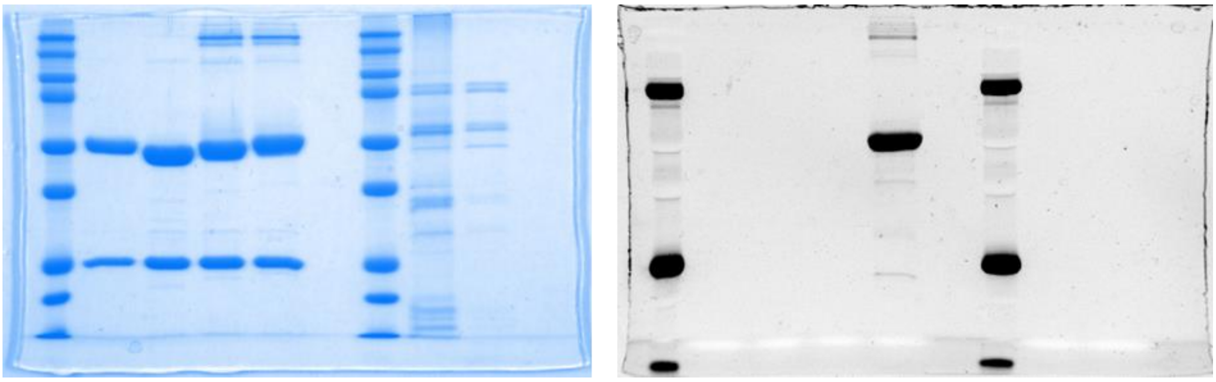

Figure S63: Full SDS-PAGE gel image of Figure S61

7. Cell biology studies

Solubility

The studied DpT-azides showed excellent solubility in pure DMSO and low solubility in culture media. All biological experiments were thus conducted using 5 mM stock solutions in DMSO that were diluted with culture medium to produce a final concentration of 0.5% DMSO for all experiments unless specified otherwise.

The ADCs were dissolved in PBS buffer to prepare 0.1 mM stock solutions and were diluted with culture medium to produce a final concentration of 0.5% PBS for all experiments unless specified otherwise.

Cell viability experiments

MCF-7 and SK-BR-3 cells were harvested, pelleted by centrifugation, and resuspended in cell culture medium. The MTT assays were carried out in 96 well plates at a starting density of 1500 cells per well in 100 µL of culture medium. The next day, a dilution series of 7 different concentrations was prepared for each evaluated compound. After 24 h of incubation, the cells were treated with another 100 µL of culture medium containing the prepared concentrations of the respective compounds to reach final DMSO/PBS concentrations of 0.5% and a final volume of 200 µL in each well. As a negative control, one series of cells was treated with the vehicle only (0.5% DMSO/PBS). The cells were then incubated with the compound for 72 hours or 120 hours, as indicated, followed by an addition of 50 µL PBS containing MTT dye (2.5 mg/mL). After a further incubation of 2 h, the medium was aspired off, and 200 µL DMSO was added to solubilize the precipitated formazan dye. The formazan absorption was measured on a BMG LABTECH CLARIOstar Plus plate reader at 550 nm, using a reference wavelength of 620 nm. Using the mean absorbance of each set of identically treated wells, a dose-response curve was produced and normalized to the wells containing untreated cells to allow for plate-to-plate comparison. The dose-response curves were then subjected to non-linear regression analysis performed in Microsoft Excel to determine IC_{50} values. Data is shown as the mean of three replicate experiments, and error bars represent the standard deviation.

Proliferation profiles

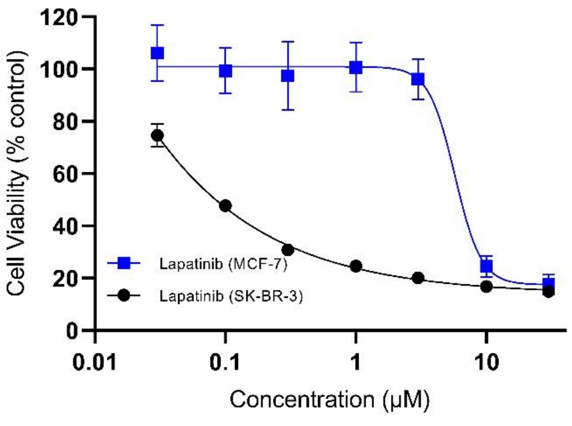

Figure S64: Proliferation profiles MCF7 and SK-BR-3 cells after 72 h incubation with lapatinib.

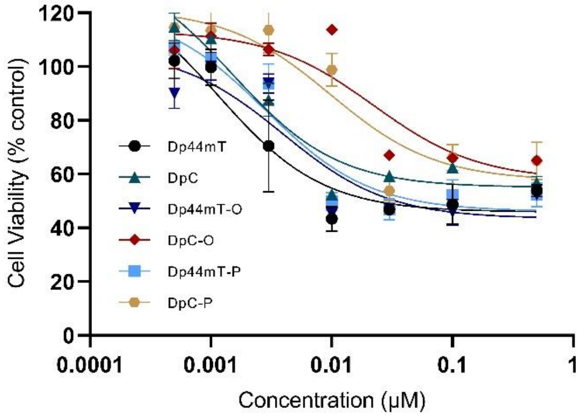

Figure S65: Proliferation profiles SK-BR-3 cells after 72 h incubation with DpT azides; Dp44mT and DpC were included as positive control.

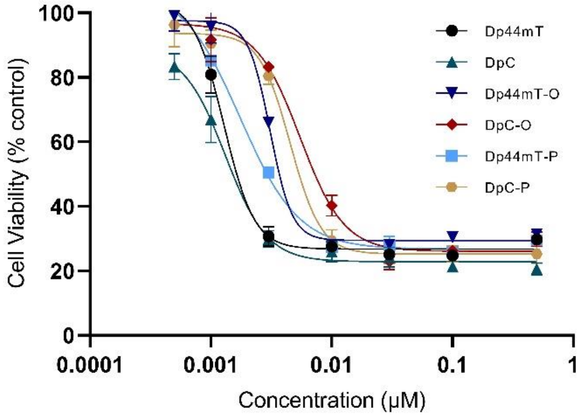

Figure S66: Proliferation profiles MCF-7 cells after 72 h incubation with DpT azides; Dp44mT and DpC were included as positive control.

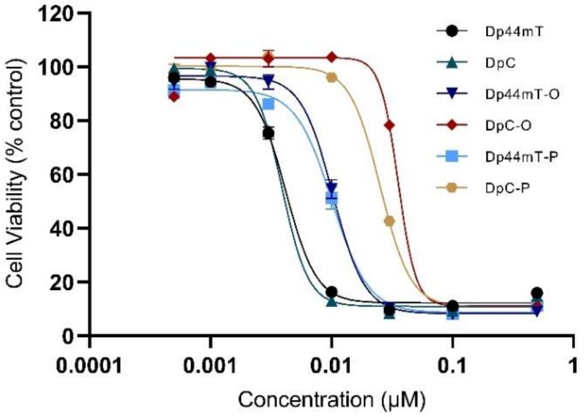

Figure S67: Proliferation profiles SK-BR-3 cells after 120 h incubation with DpT azides; Dp44mT and DpC were included as positive control.

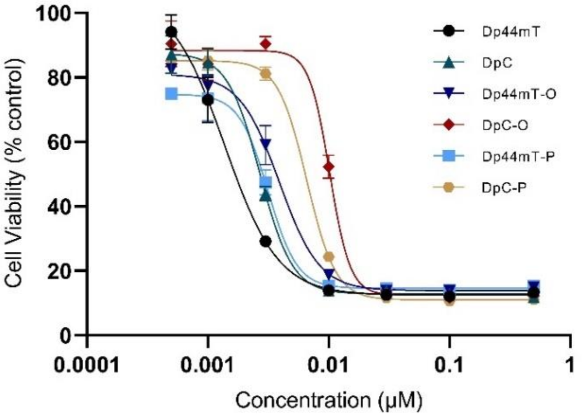

Figure S68: Proliferation profiles MCF-7 cells after 120 h incubation with DpT azides; Dp44mT and DpC were included as positive control.

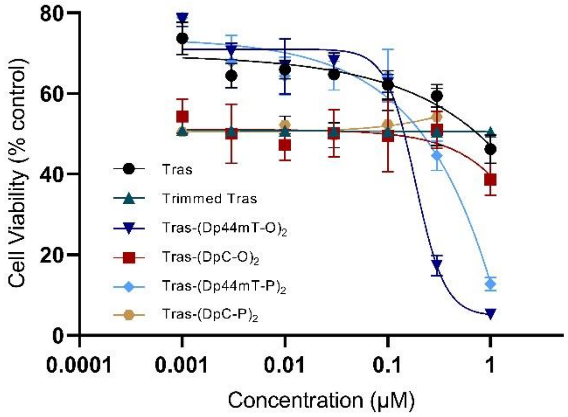

Figure S69: Proliferation profiles SK-BR-3 cells after 120 h incubation with Tras-DpT conjugates; Trastuzumab and Trimmed Tras were used as controls.

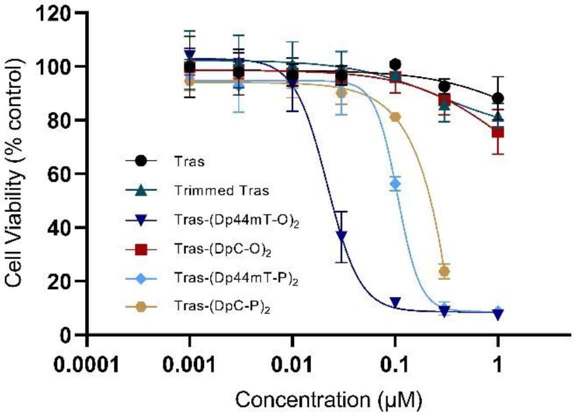

Figure S70: Proliferation profiles MCF-7 cells after 120 h incubation with Tras-DpT conjugates. Trastuzumab and Trimmed Tras were used as controls.

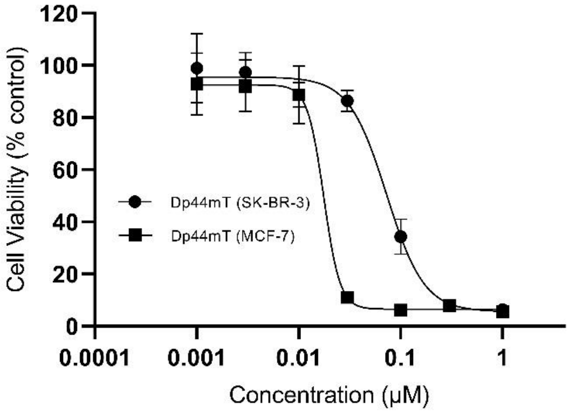

Figure S71: Proliferation profiles MCF7 and SK-BR-3 cells after 2 h incubation with Dp44mT, followed by media replacement and incubation for a further 118 h.

IC_{50} (nM)		
**Compound**	**SK-BR-3**	**MCF-7**
**Dp44mT**	4.4 ± 0.2	1.9 ± 0.4
**DpC**	4.1 ± 0.3	2.7 ± 0.3
**1**	12.9 ± 4.1	3.9 ± 1.2
**2**	31.8 ± 0.8	10.6 ± 0.5
**3**	10.1 ± 0.3	2.9 ± 0.1
**4**	29.6 ± 0.1	6.4 ± 0.1
**5**	145.5 ± 11.9	25.7 ± 5.5
**6**	>1000	>1000
**7**	228.3 ± 60.1	103.5 ± 1.4
**8**	>1000	113.8 ± 2.0
**9**	xx	93.5 ± 7.5

Table S1: Consolidated IC_{50} values.

Western Blot

Cells were grown on a 10 cm culture dish until 90% confluency was reached. The media was aspired off, and the cells were washed three times with PBS. Radioimmunoprecipitation assay (RIPA) lysis buffer with protease and phosphatase inhibitors was added, and the cells were scraped from the dish and collected in a 1.5 mL Eppendorf tube. The RIPA mixture lyses the cells, prevents proteolytic degradation, and keeps the phosphorylation state of the sample the same during protein extraction. Cells were stored on ice for 20 min, resuspended by vortexing, and then ultrasonic lysis was used in pulse mode (three sets of 10 sec each) on the cell suspension to guarantee that proteins of the membrane were released. The cell suspension was centrifuged for ten minutes at 4°C at 13200 rpm (Eppendorf® Microcentrifuge 5415R,

Hamburg, Germany), and the supernatant, which contains the proteins, was transferred to new Eppendorf tubes. Next, the protein concentration was measured against a BSA calibration curve in a Bradford protein concentration assay using a Nanodrop 2000c spectrophotometer (VWR Life Science, Erlangen, Germany). After that, the lysates were diluted with the lysis buffer to reach the same relative concentration in all the samples. 24 µl of each cell lysate were then loaded with 5 µl of the prestained marker into the gel pockets of a 10% SDS-PAGE gel. The separated proteins were transferred from the gel onto the nitrocellulose membranes using the BioRad Tans-Blot Semi-Dry transfer cell (BioRad Laboratories, Hercules, California) at a constant current of 300 mA for 30 min. Before the antibody incubation, the membranes are blocked with a 5% milk in TBS-T solution for 1 h. The membranes are incubated with diluted antibodies against p/tAKT (60 kDa) and p/tERK 1/2 (44,42 kDa) separately, after the blocking, overnight at 4°C. Then, the membranes were washed with TBS-T (three times for 10 min each) and next, incubated with the secondary horseradish peroxidase-conjugated antibodies to anti-rabbit (dilution 1:5000) in 5% milk TBS-T for one hour at 4°C. In the last step, the membranes were rewashed with TBS-T (two times for 10 min each) and kept in TBS until the detection. To detect the proteins by chemiluminescence, a ChemiDocMP (BioRad Laboratories, Hercules, California) was used for 10 min in signal accumulation mode. Therefore, each membrane was covered in 1 ml of the SuperSignal West Pico Chemiluminescence Substrate (ThermoFisher) and placed in the detector.[6]

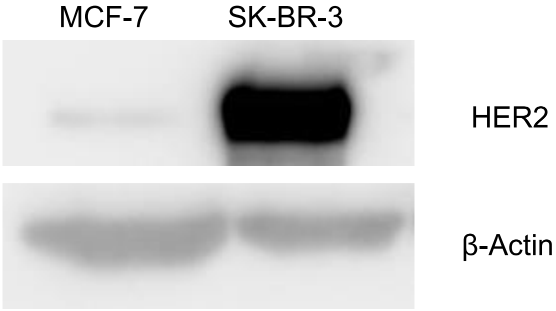

Figure S72: The Western Blot displays the difference in HER2 expression in MCF-7 and SK-BR-3 cells.

Uptake Studies

Live-cell imaging was carried out in 8-well cell culture chamber slides from ibidi at a starting density of 20000 cells per well in 200 µL of culture medium. The cells were incubated for 24 h at 37 °C under a 10% CO_2 atmosphere. Then, the existing media was replaced with 200 µL media containing compound **9** at 2 µM concentration. As controls, untreated SK-BR-3 and MCF-7 cells were prepared under the same conditions but without the active compound. After 2 h or 24 h of incubation, as appropriate, the media was removed, and the cells were washed twice with DMEM medium without phenol red (clear medium) and then covered with 200 µL clear medium for the duration of microscopic investigations. The imaging was carried out at 37 °C using a Leica TCS-SP8 confocal laser scanning microscope equipped with a pulsed white light laser (WLL) (470 nm – 670 nm) using hybrid single molecule detectors (SMD). Compound **9** was excited at 550 nm using the WLL laser at a laser power of 20, and the emission was collected between 570 and 600 nm at a detector gain of 225. Images were

acquired at 2048 x 2048 pixels and processed using LASX office program. The brightness of the images acquired after 24 h was then measured using Fiji (version 1.54f).[7]

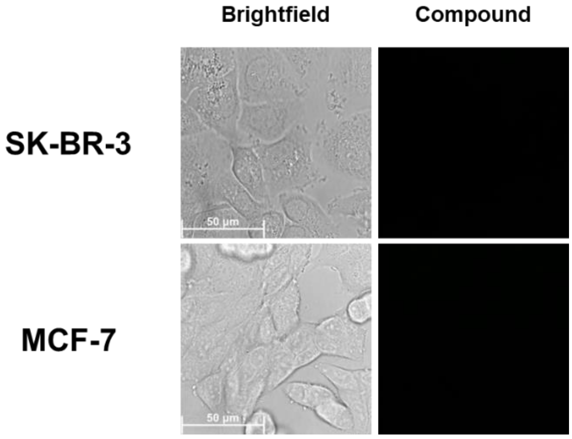

Figure S73: Control images from live-cell confocal microscopy. This includes cells treated with DMSO-containing media as the negative control. The images were acquired using the aforementioned settings.

BLI studies

Measurement of the binding of the antibody constructs for HER2 by biolayer interferometry[8]

The binding of the antibody constructs was measured by biolayer interferometry (BLI). All samples (trastuzumab, tras-$TAMRA_2$, tras-$(Dp44mT\text{-}ortho)_2$ and cetuximab) were prepared at 2.5 µg mL^{-1} in PBS with 0.1% BSA and 0.02% Tween 20. Biotinylated HER2 (Her2/ERBB2 Protein, Human, Recombinant (His & AVI Tag) was prepared at 5 µg mL^{-1} in PBS with 0.1% BSA and 0.02% Tween 20.

Streptavidin (SAX) Biosensors (ForteBio) were used to immobilize the biotinylated HER2. The assay was carried out with the following steps: incubation in PBS with 0.1% BSA and 0.02% Tween 20 for 1 min (baseline 1), incubation in solution of biotinylated HER2 for binding to the biosensors for 1 min (loading), incubation of the biosensors in PBS with 0.1% BSA and 0.02% Tween 20 for 1 min (baseline 2), incubation of the biosensors with solutions containing the antibody constructs for 300 s (association), incubation of the biosensors in PBS with 0.1% BSA and 0.02% Tween 20 for 300 s (dissociation).

8. References

1 M. T. Marty, A. J. Baldwin, E. G. Marklund, G. K. A. Hochberg, J. L. P. Benesch and C. V. Robinson, *Anal. Chem.*, 2015, **87**, 4370–4376.

2 K. Tanaka, W. R. Ewing and J.-Q. Yu, *J. Am. Chem. Soc.*, 2019, **141**, 15494–15497.

3 J. E. Parks, B. E. Wagner and R. H. Holm, *J. Organomet. Chem.*, 1973, **56**, 53–66.

4 P. Reimerová, J. Stariat, H. Bavlovič Piskáčková, H. Jansová, J. Roh, D. S. Kalinowski, M. Macháček, T. Šimůnek, D. R. Richardson and P. Štěrbová-Kovaříková, *Anal. Bioanal. Chem.*, 2019, **411**, 2383–2394.

5 V. Faid, Y. Leblanc, M. Berger, A. Seifert, N. Bihoreau and G. Chevreux, *Eur. J. Pharm. Sci.*, 2021, **159**, 105730.

6 I. Schuler, M. Schuler, T. Frick, D. Jimenez, A. Maghnouj, S. Hahn, R. Zewail, K. Gerwert and S. F. El-Mashtoly, *The Analyst*, 2024, **149**, 2004–2015.

7 J. Schindelin, I. Arganda-Carreras, E. Frise, V. Kaynig, M. Longair, T. Pietzsch, S. Preibisch, C. Rueden, S. Saalfeld, B. Schmid, J.-Y. Tinevez, D. J. White, V. Hartenstein, K. Eliceiri, P. Tomancak and A. Cardona, *Nat. Methods*, 2012, **9**, 676–682.

8 C. T. Orozco, Bersellini ,Manuela, Irving ,Lorraine M., Howard ,Wesley W., Hargreaves ,David, Devine ,Paul W. A., Siouve ,Elise, Browne ,Gareth J., Bond ,Nicholas J., Phillips ,Jonathan J., Ravn ,Peter and S. E. and Jackson, *mAbs*, 2022, **14**, 2095701.
